# A Blockchain Platform for Vaccine Beneficiary Registration, Side Effects, Cold Supply Chain, and Personnel Training and Employment Management

**DOI:** 10.3390/s26206524

**Published:** 2026-10-14

**Authors:** Alia Al Sadawi, Malick M. Ndiaye, Israa Al Khaffaf, Omar Abdulkarim

**Affiliations:** Department of Industrial Engineering, College of Engineering, American University of Sharjah, Sharjah P.O. Box 26666, United Arab Emirates; mndiaye@aus.edu (M.M.N.); g00016294@aus.edu (I.A.K.); b00096470@alumni.aus.edu (O.A.)

**Keywords:** blockchain, cold supply chain, IoT, side effects, security, smart contract, transparency, traceability, trust

## Abstract

The world has faced pandemics throughout its history. The most recent, COVID-19, took lives and disrupted healthcare and economies. COVID-19 vaccine programs were launched worldwide; however, the record time in which vaccines were developed raises questions about side effects and their severity. Also, as vaccines are delicate medical products, their cold supply chain must be continuously tracked and monitored to ensure their efficacy. Moreover, vaccine supply chain management is complex and requires trained staff to handle its different activities. As such, it is important to connect beneficiaries’ symptoms and registration systems with the vaccine supply chain to enable healthcare authorities to investigate whether observed side effects may be linked to cold chain handling conditions or production factors. Consequently, the need arises for a system that manages trained personnel certificate generation, which could also be used as a verification tool for employing the right experts for the right job. Therefore, our study developed a blockchain platform that integrates individuals’ registration and side effect data with vaccine cold supply chain information. The developed platform also includes a certification system for cold supply chain personnel training to support authenticated certificate generation and role assignment management. Blockchain was selected due to its features of security, decentralization, traceability, and trust. The key contributions of this work lie in providing a digital collaborative management and data tracing platform for vaccine side effects that are linked to beneficiaries’ registration and cold supply chain data to enable healthcare authorities to investigate whether any observed side effects may be linked to cold chain handling conditions or production factors while managing the issuance of certificates for trained cold supply chain personnel and controlling their employment in the correct chain(s). Our platform creates training certificate IDs without a central entity to regulate certificate generation and uses the created certificate information to control the employment of personnel with the appropriate training in the right chain to perform the right role. Our platform identified personnel employed in any chain where violations were recorded and side effects were subsequently reported, and presents their training and employment data, providing the interconnected data record necessary for accountability. The platform was tested successfully, producing the desired outcome of secure, traceable, and transparent vaccine data sharing, linking, and verification.

## 1. Introduction

**Figure 1:**
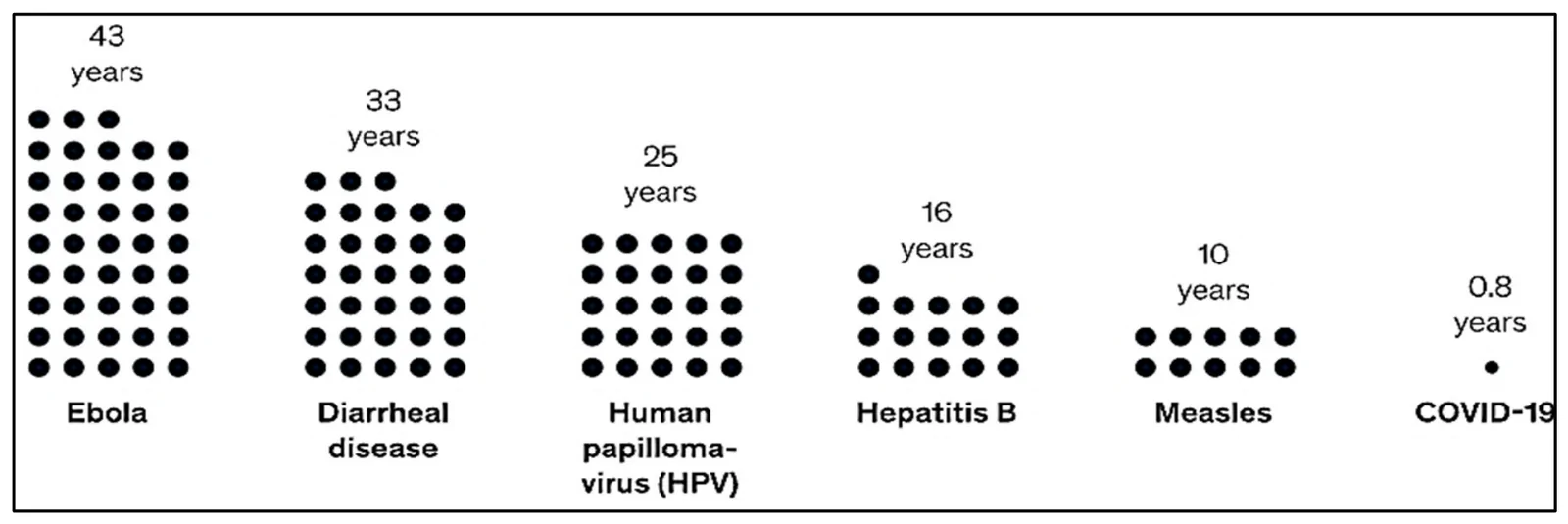
The time from infectious agent identification to vaccine license in the U.S.A. [3].

Throughout history, infectious diseases and pandemics have frequently spread. Some of the worst pandemics have impacted humanity, taking lives and causing economic recessions. Major pandemics include cholera, the plague, and severe acute respiratory syndrome coronavirus (SARS-CoV). COVID-19 (novel coronavirus disease) is the latest pandemic that affected the entire planet and led to millions of fatalities. The World Health Organization (WHO) announced COVID-19 as a global pandemic [1]. To date, over 704 million COVID-19 cases have been reported, including 7 million fatalities [2]. Economically, among all worldwide recessions since 1990, the COVID-19 recession was characterized by the fastest and deepest declines in consensus growth expectations. During the peak of COVID-19, healthcare systems faced great burdens. Also, the lack of vaccination or treatment made it difficult to prevent disease outbreaks. As vaccination is the sole means to limit the spread of COVID-19, it became a top priority for public health authorities to create vaccines that are both safe and effective. The minimum recorded time for vaccine development, which took four years from virus sample to approval, was in the case of developing the mumps vaccine [3]. After the SARS-CoV-2 virus was determined as the outbreak’s primary cause, with the aid of 21st-century technology, the mRNA1273 COVID-19 vaccine broke a record by entering clinical trials (NCT04283461) in only 69 days [4]. Refer to Figure 1 for a comparison.

Several stages of vaccine development were condensed or eliminated in order to produce the vaccinations quickly and clinical trials were accelerated [5].

The development of vaccines in record time raised concerns about possible side effects, especially for older adults, children, and people with chronic diseases or special needs. Therefore, it is vital to gather data on possible side effects. In order to research the adverse effects, this data needs to be linked to a secure, intelligent system for registering beneficiaries as well as the vaccine cold supply chain. It is extremely important to gather structured, verifiable data on observed side effects and investigate whether they are associated with particular patient populations. Additionally, it is critical to enable healthcare authorities and clinical investigators to assess and investigate whether any observed side effect may be linked to production factors or logistical mistakes brought on by violations of vaccine safety and temperature regulations. As most vaccines are sensitive to temperature, they must be stored and transported within a specific temperature range to maintain effectiveness. Deviations from recommended temperature ranges can lead to loss of vaccine potency, failure to provide adequate protection, and potentially harm the person who receives the vaccine, resulting in negative side effects. Therefore, healthcare providers, vaccine manufacturers, and regulatory authorities need to remain vigilant and ensure that all aspects of the vaccine supply chain are properly monitored and controlled. Table 1 presents the recommended storage temperatures for some of the COVID-19 vaccines.

Potential side effects shall be investigated through health authorities’ follow-ups with patients, especially children, older adults, and people with chronic diseases or special needs.

Furthermore, the fact that vaccines are sensitive to temperature and light raises the need for professional handling of their complicated supply chain, from manufacturer facilities to medical centers. As the cold supply chain of vaccines has a decisive effect on their efficacy, well-trained personnel are an extremely important factor in successful cold supply chain management. Therefore, it is necessary to have a trusted system to certify and verify the qualifications of people taking roles in the cold supply chain who handle temperature-sensitive vaccines. From surveying the literature, we found that a specialized training certification system for cold supply chain personnel has not been proposed before. The lack of such a system creates a gap in supply chain management practices and is a source of risk to the safety of the transferred medicines.

The goal of this work is to use technology to support the healthcare system and ensure the vaccine’s safety. Blockchain technology was selected due to its ability to support secure, verified, and transparent storage and transfer of digital assets [8]. Also, blockchain supports smart contracts, which are designed to be implemented automatically when certain conditions are met [9], increasing efficiency and eliminating the need for trusted third parties, which can streamline the process of conducting business [10,11]. Blockchain has proven its reliability and robustness when applied in diverse fields, including supply chain management [12], voting systems [13], identity verification, digital trading [14], and carbon emissions trading [15]. However, research about blockchain applications in the field of vaccination is considered to be in its infancy. This is because the spread of COVID-19, which served as a wake-up call, came as a surprise. Therefore, there is room for innovative studies in this area. Vaccine safety and stakeholder trust are two main aspects that could be addressed through blockchain features of visibility, traceability, and immutability [16].

This research presents a blockchain platform for vaccine program safety and management. Our blockchain platform collects, stores, connects, and manages cold supply chain data, including vaccine lots’ location, temperature, and light exposure, beneficiaries’ registration and side effects information, and cold supply chain personnel training certification and employment information in a secure, traceable, and transparent manner.

The key contributions of our work lie in providing a digital collaborative management and data tracing platform for vaccines’ probable side effects that are linked to beneficiaries’ registration and cold supply chain data to enable healthcare authorities and clinical investigators to assess and investigate whether any observed side effects may be linked to cold supply chain mishandling or production issues. The platform additionally introduces a side effect amendment model that reconciles blockchain immutability with medical record governance requirements. Our system extends its operation to manage the issuance of certificates for trained cold supply chain personnel and to control their employment at the correct chain(s). Our proposed platform enables the creation of training certificate IDs without the need for a central entity to regulate the training certificate generation process or enforce its rules. It also automatically uses the generated certificate information to control the employment of employees with the appropriate training in the right chain to perform the right role. This system expansion facilitates identifying the personnel employed in any chain where cold chain violations were recorded and side effects subsequently reported, and displays personnel training, certification, and employment information, providing the interconnected accountability data necessary for investigation. The smart contract acts as an automated compliance gate for personnel assignment, making the blockchain record simultaneously a violation log, an employment record, and a qualification verification in one immutable, tamper-resistant entry.

Our research is particularly relevant for certain segments of society, such as older adults, children, and people with chronic diseases or special needs. Furthermore, the continued emergence of COVID-19 variants underscores the need to sustain and improve vaccination programs. This is in addition to the emergence of other diseases and pandemics, such as monkeypox. Our blockchain-based research offers the following contributions to the literature and scientific field:

- Provides a secure, transparent, and traceable digital system design that connects vaccine side effects with beneficiaries’ health records and vaccines’ cold supply chain using blockchain and smart contracts, introducing forensic investigation support through bidirectional traceability and enabling a class of demographic-clinical-supply chain analysis.
- Develops a trusted, verified, and transparent blockchain-based cold supply chain personnel certificate issuance and verification and role assignment system, where the smart contract acts as an automated compliance gate. The system supports identifying the personnel employed in any chain who witness violations and demonstrates related information about personnel training, certification, and employment,
- Develops a smart contract for vaccine beneficiaries’ registration process characterized by authenticity, transparency, security, and immutability,
- Develops a smart contract-based cold supply chain system that supports transparent and secure health and safety conditions violations management while ensuring the awareness of all stakeholders,
- Develops a smart contract that serves as a trusted, traceable, and transparent vaccine side effect information database to study the aftermath of the vaccine campaign, evaluate the effectiveness and safety of each vaccine type, and deal with any undesirable consequences. It also enables healthcare authorities and clinical investigators to assess and investigate whether the noticed side effect may be linked to a problem with the cold supply chain or the production process,
- Designs and develops a smart contract that practically implements and automates the cold supply chain personnel certification issuance and role assignment processes.

Our designed system, using blockchain technology known for providing a single trusted source of truth, is a necessary enabler and facilitator of medical data science.

The remainder of the article is structured as follows: Section 2 surveys related research in the literature. Section 3 details the vaccine cold supply chain journey. Section 4 demonstrates the designed blockchain-based vaccine beneficiaries’ registration and side effects system and the cold supply chain management system. Section 5 proposes the vaccine beneficiaries’ registration and side effects system and the cold supply chain management system implementation and testing processes. Section 6 demonstrates the proposed blockchain-based personnel certification and employment system. Section 7 details the personnel certification and employment system implementation and smart contracts testing results. The comprehensive blockchain platform analysis is presented in Section 8, while the conclusion is presented in Section 9.

## 2. Literature Review

This section surveys the literature for related research. It comprises the following subsections.

### 2.1. Blockchain and Supply Chain

Blockchain implementation in the SCM is currently going through rapid development [17]. A review of the literature showed that blockchain has tackled many SCM industries, such as food and agriculture [18], pharmaceuticals [19], and automotive [20], among others. Utilizing blockchain to improve traceability in SCM was addressed by many studies, such as [21]. Blockchain traceability and visibility throughout the whole supply chain result in the optimization of information flow that reduces costs. In this regard, Ref. [22] developed an intriguing architecture built on blockchains to support peer-to-peer shipment-tracking data for suppliers and customers to enhance the supply chain’s visibility of physical distribution. A similar study, Ref. [23], presents a blockchain distribution model for the COVID-19 vaccination that authenticates each operation and records modifications. Also, study [24] looked into the benefits and costs of using blockchain technology in a supply chain in order to increase consumer awareness of traceability. Study [25] established the StaTwig platform, which connects vaccination manufacturers and consumers using blockchain technology to improve traceability and boost distribution efficiency. Furthermore, Ref. [26] laid the theoretical framework for potential future applications of blockchain technology by exploring the difficulties in using it in sustainable supply chains. Study [27] proposed CrossChain-VaxTrace, a blockchain architecture integrating cross-chain interoperability using proof-of-authority consensus, federated graph neural networks for privacy-preserving demand forecasting and route optimization, and edge AI nodes for real-time cold chain monitoring across multi-organizational vaccine supply chain networks. Study [28] proposed a consortium blockchain platform integrating the fuzzy analytic hierarchy process (FAHP) for evaluating and selecting third-party logistics providers in vaccine cold supply chains, using smart contracts and IoT sensors to automate compliance monitoring, temperature tracking, and logistics provider performance evaluation across four key stakeholders. Ref. [29] presented ColdNet, an architecture integrating IoT device attestation with Ethereum smart contracts for immutable monitoring of vaccine temperature, humidity, and luminosity during transport and storage, validated through a real-world case study. Furthermore, Ref. [30] proposed an integrated framework combining blockchain, IoT, and machine learning for sustainable vaccine cold chain management, supporting real-time temperature monitoring, end-to-end traceability, vaccine demand forecasting using the Informer model, and stakeholder trust assessment through BERT-based sentiment analysis. Moreover, Ref. [31] introduced a federated blockchain framework integrating Hyperledger Fabric, smart contracts, federated learning, and edge computing for decentralized vaccine supply chain management, with experimental evaluation demonstrating improvements in temperature anomaly detection speed, transaction throughput, and breach detection accuracy. Also, Ref. [32] proposed a Hyperledger Fabric-based permissioned blockchain framework integrated with IoT sensors for cold chain logistics, enabling role-based access control among producer, carrier, and retailer organizations with immutable environmental data recording, validated through systematic performance testing using Hyperledger Caliper.

### 2.2. Blockchain in Healthcare

The number of research articles and papers exploring the implementation of blockchain in healthcare is now expanding dramatically [33]. Several studies in the literature reviewed the possible uses of blockchain in healthcare, including [34,35,36]. Blockchain has the ability to reshape healthcare by improving data management, enhancing patient privacy, and increasing medical supply chain efficiency. Multiple studies were carried out on authorizing the use of personalized health data, including data preservation, such as [37]. Study [38] tackled the sharing of medical records, while [39] discussed medical insurance and tracking and monitoring of health status. Promoting the management of social and public health is another significant topic that is addressed by blockchain. It includes practices such as medication administration, which is studied by [40], and pandemic prevention. Some articles also examined enhancing the credibility of medical research and teaching biomedical research, clinical research, and medical education, such as [41].

Additionally, the literature revealed that a good number of studies anticipated using blockchain in electronic health records (EHRs), such as [42,43]. These studies addressed utilizing blockchain to provide capabilities for data monitoring and exchange between clinicians and researchers within the healthcare team. This assists in managing and analyzing healthcare data and building trust, interconnection, and enhanced security [44]. Other studies, such as [45,46], suggested using blockchain to create a system for personal health records that might connect institution-specific EHRs for patients. Furthermore, publications [47,48] discussed m-health and patient-controlled sensor data collection, storage, and exchange. Also, articles [49,50] examined the application of blockchain in clinical trial systems. Blood donation is an important aspect of healthcare that can be enhanced by blockchain. According to [51], it is possible to automate blood donation administration in a way that is private, safe, reliable, and auditable. Managing and tracking personal protective equipment (PPE) products in the supply chain is also made possible by blockchain technology. Ref. [52] described a blockchain-based strategy for reshaping PPE supply chain processes using smart contracts. A blockchain-based eHealth system was developed by [53], which enables patients to audit health information and spot data misuse. Moreover, they employed attribute-based proxy re-encryption to ensure precise control for medical data access. Also, Ref. [54] created a system for managing consent for clinical trials called SCoDES. To avoid the need for an ad hoc intermediary, blockchain stores all permit files with trust assurances. Furthermore, blockchain could be utilized to improve the integrity and transparency of the drug supply chain. Study [55] explored the challenges facing the pharmaceutical supply chain, such as drug fraud, and the potential of solving them by employing digital technologies.

### 2.3. Blockchain and COVID-19 Pandemic

The COVID-19 pandemic had a profound impact on healthcare, which initiated the deployment of advanced digital systems. A literature review found relatively little research on blockchain and the COVID-19 pandemic. Three main blockchain applications related to COVID-19 were highlighted in the literature, including the issuance of vaccine immunization passports, traceability of contacts, and pandemic management and monitoring. For example, study [56] developed a blockchain-based solution utilizing smart contracts and tokens to support movement passes and promote social distancing. Also, study [57] proposed a contact tracking initiative built on the blockchain called P2B Trace, which protects user privacy. In a separate study, Ref. [58] introduces a blockchain system for tracking contact while maintaining privacy called BeepTrace, in which blockchain and hash functions are utilized to create a link between users and authorized solvers to enable checking users’ ID and location data. Furthermore, Ref. [59] suggested a blockchain solution that combines testing and vaccination to develop an instant tamper-proof authentication of COVID-19 test results that could be used as “Test Certificates”. Study [60] proposed a blockchain system for issuing and verifying COVID-19 tests and vaccine certificates called NovidChain. The platform is built to fulfill top-level privacy and security needs for personal information and complies with GDPR and KYC requirements as well as guaranteeing the user’s independence.

Moreover, blockchain in combination with IoT was suggested to manage and control vital provisions of supply chains that require precise monitoring, especially during the COVID-19 pandemic. For instance, Ref. [61] created end-to-end tracking and visibility for the delivery of the COVID-19 vaccination using IoT sensor devices integrated with self-enforcing smart contracts. The study included side effect follow-up information files saved on-chain; however, the study suggested that the self-reporting of side effects is not considered practical or adequate. Also, the research did not include the government’s role in the system design. Study [62] used blockchain smart contracts to handle the forward supply chain and waste management of COVID-19-associated medical equipment to ensure transparency and efficiency. Nevertheless, the suggested solution did not take into account beneficiaries’ administration registration and vaccines’ possible side effects data collection processes.

Furthermore, study [63] proposed the Predictive BlockVax Distribution Network (PBDN), a framework that integrates blockchain using Hyperledger Besu with machine learning for vaccine demand forecasting and distribution transparency. The framework addressed inefficiencies in vaccine distribution and lack of transparency in healthcare supply chains during pandemics. Study [64] integrated blockchain with self-sovereign identity (SSI) for privacy-preserving vaccine supply chain management, enabling decentralized identity verification for supply chain stakeholders while anchoring cryptographic proofs and audit logs on-chain. Also, study [65] developed a blockchain-enabled multi-channel COVID-19 vaccine supply chain network model integrating multi-stage stochastic programming to handle supply uncertainty and fuzzy programming for uncertain vaccine price and quality, with blockchain incorporated as a transparency-maximizing objective in the mathematical optimization model.

Ref. [66] proposed a blockchain-based cyber-physical ecosystem using Hyperledger Fabric, integrating GS1-standard serialization with IoT temperature sensors to track vaccine custody across the supply chain, with automated purchase-order abortion when cold chain temperature tolerances are breached; it was fully deployed on AWS using Docker Swarm with a working Angular frontend.

### 2.4. Applications of Blockchain in Certificates

The development of certificate authentication solutions using blockchain technology was discussed by many researchers. For example, Ref. [67] emphasized the potential of blockchain in verifying certificates and addressing the limitations of paper certificates. Also, Ref. [68] proposed EduCTX, a global higher education credit platform based on blockchain technology. Additionally, study [69] presented a blockchain education platform for issuing, validating, and sharing certificates. Study [70] presented a hybrid blockchain-based Academic Credential Verification System (B-ACVS) that combines the benefits of public and private blockchains to ensure a secure and efficient verification process. The private blockchain was utilized for storing sensitive academic data, and a public blockchain was used for verification and transparency purposes. The B-ACVS allowed educational institutions to issue digital certificates and record them on the private blockchain. These certificates could then be easily verified by relying on the public blockchain’s decentralized and transparent nature. Furthermore, Ref. [71] proposed a blockchain solution to enhance the security of X.509 public key infrastructures (PKIs). It suggested using certificate transparency (CT) and revocation transparency (RT) to address the potential compromise of certification authorities (CAs). The system enables web servers to publish CA-signed certificates and revocation information on a global certificate blockchain. Browsers can then verify the authenticity of certificates by comparing them with blockchain records.

It was noticed that different blockchain platforms were proposed for issuing, managing, and verifying educational certificates, such as the Bitcoin blockchain [72], Ethereum [73], dual platforms of Bitcoin and Ethereum [74], and Hyperledger Fabric [75]. For instance, study [76] developed a decentralized application using Ethereum blockchain to prevent certificate counterfeiting. Also, study [77] utilized the Hyperledger Fabric blockchain platform to ensure that certificates are issued with the domain owner’s approval. Moreover, Ref. [78] implemented a consortium blockchain system with a secret sharing scheme to validate digital certificates and protect user information. However, these studies faced challenges such as the need for additional scanning apps, delayed certificate validation, scalability issues, and limited transaction capacity.

The research in the field of blockchain-based employment and skill certification is still in its early stages. Study [79] proposed a blockchain system to protect the rights of temporary employees, ensuring fair and legal payouts based on work performance. This system also facilitates automatic contract management through smart contracts. Moreover, Ref. [80] introduced a blockchain-powered skill verification system that addresses challenges in verifying skills. The system allows individuals to store and validate their skills on the blockchain, providing increased trust and efficiency in the verification process. Another article by [81] proposed a two-stage blockchain system called E2C-Chain to address the challenges of background screening for employment, education, and skill verification. The system aimed to improve the efficiency and accuracy of the verification process by leveraging blockchain technology and employing a Vickrey–Clarke–Groves (VCG) incentive mechanism. Theoretical proofs and simulations were provided to demonstrate the effectiveness of the proposed system in minimizing social costs and enhancing reliability. Furthermore, Ref. [82] proposed a four-layer theoretical blockchain framework for human resource management, mapping distributed ledgers, consensus protocols, and smart contracts onto HR functions, including recruitment, credential verification, onboarding, payroll, and talent mobility, comparing blockchain-driven HR systems against traditional centralized approaches.

It is concluded that, while existing research allows educational institutions to generate diplomas and store data on the blockchain, these approaches lack mechanisms for monitoring students’ training progress.

### 2.5. Blockchain and Healthcare Certificates

Healthcare certifications are essential in ensuring the skills and capabilities of healthcare practitioners, interpreters, and other stakeholders in the healthcare ecosystem. The literature includes some recent studies that cover different aspects of certification in healthcare. These studies emphasized the importance of certification to ensure competence, credibility, and improved patient outcomes in various healthcare domains. For instance, Ref. [83] performed a systematic literature review to investigate the connection between clinical patient outcomes and nursing certification. The study explored the relationship between nurse certification and improved outcomes for patients. Study [84] proposed a blockchain-based method for the generation and verification of medical certificates in IoT-based healthcare systems. The proposed solution is utilized in healthcare applications to improve data privacy and avoid certificate forging. Furthermore, Ref. [85] introduced a blockchain framework for allowing data sharing amongst training providers using the Internet of Things (IoT). The study concentrated on showing the advantage of using a combination of blockchain and IoT in sharing training data, which could contribute to the advancement of scientific programming. In addition, study [86] introduced “CertificateChain,” a decentralized healthcare training certificate management system that utilizes blockchain and smart contracts. The study focused on exploiting the tamper-proof and transparent properties of blockchain to improve the management and verification of healthcare training certificates. The security and integrity of healthcare credentials could be enhanced by this technology, ensuring that practitioners have access to current and accurate training. However, the study lacks specific details regarding the target recipients of the training. It does not clearly indicate whether the training is intended for doctors, nurses, or other healthcare professionals. It also lacks detailed technical information regarding the design and architecture of the system. Information on the specific blockchain platform used, the data structure of certificate records, and the smart contract functionalities would be valuable for understanding the feasibility and scalability of the proposed system.

Furthermore, Ref. [87] proposed a blockchain-based system for healthcare professional credentialing, arguing that physician licenses, transcripts, work history, and liability coverage could be recorded once on a blockchain and verified by any employer via smart contracts without repeated independent verification, offering significant time and cost savings and addressing healthcare workforce shortages.

This survey of the literature shows that existing blockchain-based vaccine and healthcare systems address individual dimensions of the problem in isolation, with the majority of publications discussing theoretical concepts or recommending design structures rather than offering practical or technical implementations. Notably, no existing work addresses the certification of specialized training for medical products cold supply chain personnel. The proposed platform brings these dimensions together into a single implemented system. The integration of independently established capabilities into a coherent architecture that enables new functionality is widely recognized as a substantive form of innovation. The smartphone combined telephony, internet browsing, photography, navigation, and music playback, none of which its designers invented, yet the integration created capabilities and a user experience that none of the components could provide individually. The novelty of the proposed platform is of this kind, and extends beyond integration to architectural properties that, to our knowledge and from our survey of the literature, have not been simultaneously achieved in any existing system.

Firstly, the platform introduces forensic investigation support as its central contribution, enabling bidirectional forensic traceability that tracks forward from production to patient and backward from a reported adverse effect through the complete cold chain, including the specific dose administered, the vaccine lot it came from, the temperature and light violation history of that lot across all supply chain stages, and the certified personnel assigned to each stage where a violation occurred. No existing system provides the interconnected data record needed to investigate whether an observed adverse effect may be linked to cold chain handling conditions or production factors.

Secondly, the platform enables a class of demographic–clinical–supply chain analysis that structurally cannot exist without integration. By storing beneficiary demographic data, including age, gender, chronic disease history, and special needs, alongside adverse effect records and cold chain violation histories, the platform allows health authorities to identify whether specific patient populations, such as older individuals, patients with chronic diseases, or those with special needs, are disproportionately affected by lots that experienced cold chain violations. This kind of analysis is not possible in any existing system because the data is always in separate silos.

Thirdly, the smart contract itself acts as an automated compliance gate for cold chain personnel assignment. An administrator cannot, even accidentally or corruptly, assign an unqualified person to a supply chain stage, as the smart contract rejects invalid assignments automatically with no exceptions and no human override, removing human discretion from a safety-critical decision rather than relying on audit or policy enforcement after the fact. When a violation occurs, the personnel on record are permanently and irrevocably on-chain, and no manager can reassign blame after the fact.

Fourthly, the blockchain record is simultaneously a violation log, an employment record, and a qualification verification in one immutable entry. It shows who was assigned to that stage, what their certification covered, when they were certified, and that their assignment was guaranteed valid at the time. No audit, inspection, or investigation is needed to reconstruct accountability, as the complete evidence is permanently and tamper-resistant recorded on-chain, and a court, regulator, or health authority can verify everything from the chain without depending on any organization to produce documents.

Fifthly, the platform introduces the amendment side effect record model that reconciles the tension between blockchain immutability and medical record governance requirements. Healthcare regulations require records to be correctable when errors occur, yet blockchain immutability prevents deletion. The proposed system resolves this by supporting the addition of correction records that reference the original rather than overwriting it. The original is preserved, the amendment is transparent, and the complete correction history is permanently and auditable recorded. This is demonstrably stronger than centralized systems where records can be silently modified without trace.

Table 2 presents a comparative analysis of the related work identified in the literature survey, evaluated against 11 dimensions: blockchain platform, IoT monitoring, beneficiary registration, adverse effects recording, cold chain traceability, personnel certification, employment verification, implementation, security analysis, and performance and scalability analysis. The table shows that no existing study addresses all 11 dimensions simultaneously.

## 3. Vaccine Cold Supply Chain

A large-scale global vaccine campaign is essential to combat the COVID-19 pandemic; however, the logistical implementation would be extremely complicated in terms of transportation and storage. It takes far more effort to distribute vaccination doses than just packing vials in boxes and loading them onto trucks. A vaccine requires perfect and extremely specialized storage conditions from the moment it departs the manufacturer until it is inoculated to a beneficiary. Vaccines must be stored and transported safely at approved temperatures, which is why the success of massive immunization campaigns depends on an efficient cold chain [88]. A cold chain is a supply chain that is temperature-controlled and uses all the necessary tools and techniques for handling vaccines. The cold chain starts with the refrigerated compartments at the production facility, continues with the distribution and transportation of the vaccine, includes proper storage at the provider medical centers and clinics, and concludes with the inoculation of the vaccine to the recipients [89]. Clearly, a key component of a successful cold chain is a temperature monitoring and tracking system. In this regard, distributors, public health workers, healthcare professionals, and manufacturers all have responsibility for managing and sustaining the cold supply chain. A breach in the cold chain due to vaccines being transported or stored outside of the specified temperature ranges may result in diminished vaccine effectiveness, providing patients with insufficient protection, patients receiving additional doses, and the public losing trust in vaccinations [89]. Health experts emphasize that vaccines’ excessive exposure to heat or cold can deteriorate their quality. This not only reduces the vaccination’s effectiveness but also wastes vaccine supplies and costs money [88]. Vaccine providers incur large amounts of losses in discarded vaccines [89], and healthcare transporters lose billions of dollars a year as a result of damaged vaccines, replacement prices, and administrative costs associated with cold chain failures [88].

Three crucial components come together to form a streamlined cold supply chain [88]:

- Equipment: Different vaccine storage choices are possible [89]. However, the optimal storage choice is typically a vaccine-specific pharmaceutical-grade unit [88] and a smart freezing container. These facilities and equipment frequently have digital temperature sensors [89].
- Specially trained staff are in charge of managing the logistical operations and vaccine campaign to ensure the proper and safe administration to beneficiaries [88].
- Processes: Vaccine manufacturers and storage and distribution centers must govern vaccine-handling processes with explicit, comprehensive, and updated instructions [88]. Since vaccines are costly, it is essential that they are properly packaged, transported, stored, unpacked, and administered [89].

The above elements work together to maintain a stable temperature throughout the vaccine cold supply chain, which is the key to a safely delivered vaccine that is effective in fighting diseases [88]. Proper management requires that the temperature level should be maintained across all stages, from supply, manufacturing, transportation, distribution, and storage to administration [90]. This necessitates a high degree of cooperation and data collection and exchange among the stakeholders, who are adjuvants and raw materials manufacturers and suppliers, vaccine manufacturers, logistics providers, climate-controlled storage units, hospital-grade freezers, temperature sensor manufacturers and healthcare providers [90]. Furthermore, governments usually play an extremely important role in managing the cold chain and the associated stakeholders [6].

**Figure 2:**
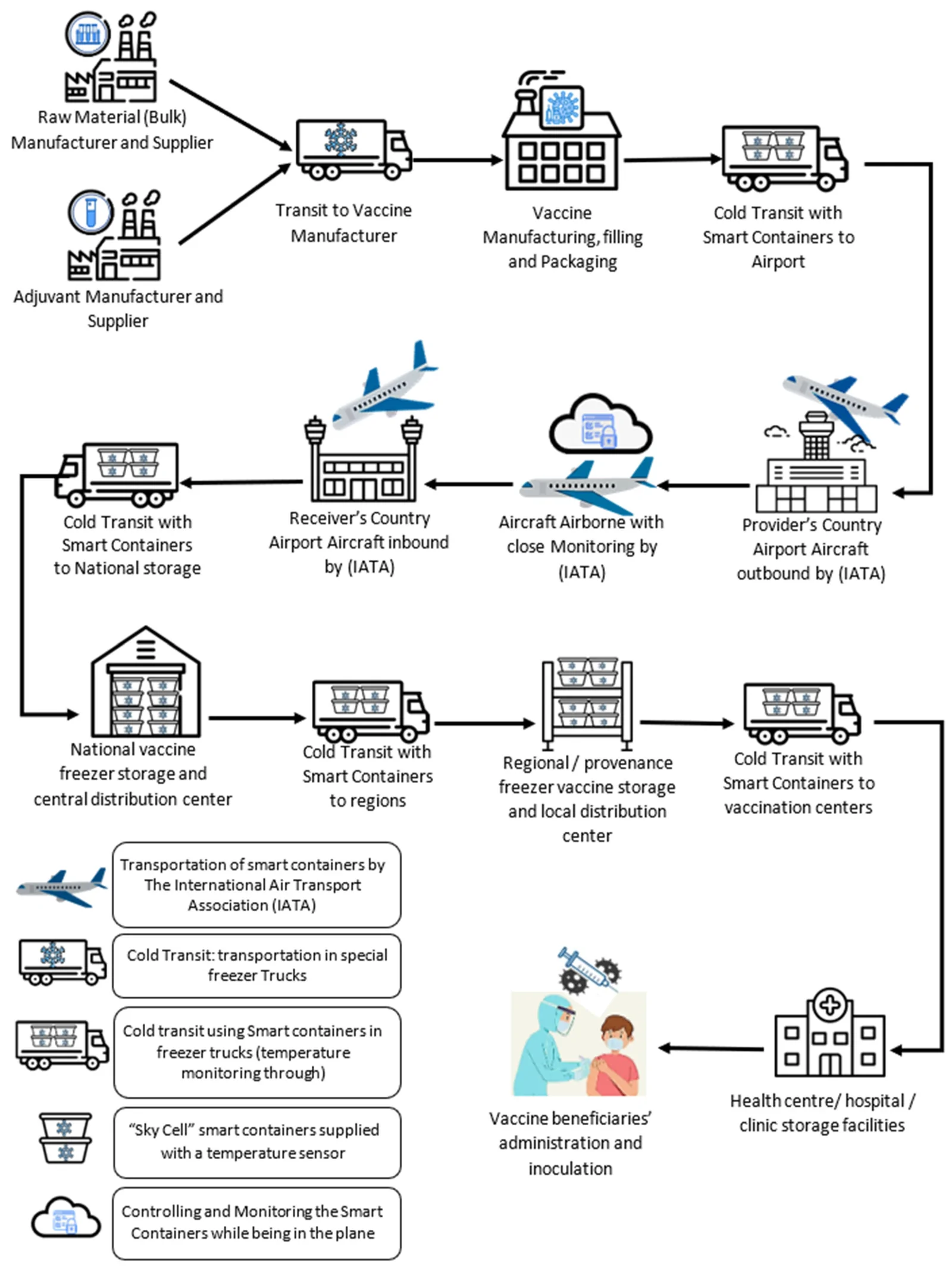
The vaccine cold supply chain.

Figure 2 shows the cold supply chain of the vaccine, which consists of the following:

- The vaccine journey starts by supplying the necessary vaccine ingredients [91], which is a crucial stage in the chain [92]. These ingredients are raw materials and adjuvants, which are components added to vaccines to aid in boosting recipients’ immune systems and improving vaccines’ effectiveness [91]. One needed adjuvant originates in South America from the bark of the Quillaja Saponaria Molina tree. Since it is only picked between November and January, there is not enough for COVID-19 vaccine manufacturing until the following harvest. Another example of an important raw material is that supplied by the pharmaceutical firm “Inovio,” whose plasmid source must match manufacturing capacity, affecting required production targets [93].
- Ingredients are transported using special trucks to vaccine manufacturing and filling to be utilized throughout the formulation, loading, and packing of vaccines. The produced vaccine is now ready to be delivered to patients [91].
- The transportation and distribution stage [91] is long, complicated, and has diverse levels depending on the destination. It includes various storage chains with multiple transit chains in between them. In most cases, vaccines have to be transported across borders to different countries, during which logistics partners play a crucial role. It involves transportation via air, sea, and land networks in pharmaceutical and temperature-controlled units to the destination healthcare centers and medical administration premises. Vaccine packs leave the manufacturing plant packed in smart containers embedded with temperature sensors to monitor vaccines’ temperature during transit stages [91]. Smart containers could also be mounted with light sensors and open/close door sensors for extra tracking and monitoring. The smart containers are then loaded into a refrigerated truck and driven straight to the airport, where they are positioned inside an aircraft. The vaccines remain under observation and control while the aircraft is outbound, airborne, and inbound until reaching their final destination [91]. The International Air Transport Association (IATA) and its members are in charge of the transportation of the vaccination shipments from the country providing them to the airport of destination. Throughout the flight, a number of staff members who have been trained in the technicalities help with the electricity supply to the refrigerators [94]. From there, a regional road transportation service firm will dispatch refrigerated trucks to move the vaccines between the storage facilities and the airport [94]. It is the company’s duty to keep vaccines within acceptable temperature ranges. Regional government representatives assist on-site with the customs clearance of vaccines at the airport [95]. Vaccines in smart containers are then transported using refrigerated trucks to national freezer storage and distribution center(s), which are ultra-cold storage facilities that can hold a large number of doses of the COVID-19 vaccine. Then, the smart containers are put back into a refrigerated vehicle and transported to local and regional storage facilities and from there to each corresponding inoculation medical center. Finally, beneficiaries are given vaccine doses [91].

The described cold supply chain applies to all types of vaccines, but it varies in terms of the temperature limits set during transit and storage chains and the number of days a vaccine can be stored or spent in transit before it expires or is damaged. Vaccines are delicate, expensive products of great importance to national health systems and economies; thus, efficient management of their cold supply chain is essential. In order to handle the vaccine cold chain logistics effectively, activities must be carefully coordinated to guarantee that the efficacy of the vaccines is maintained by monitoring temperature and maintaining updated records of the measured temperature data for traceability [95]. Furthermore, appropriate, beneficial, and successful cold chain management entails not only ensure that the temperatures required to ensure vaccine viability are always maintained, but also that sufficient technologies are used to enable stakeholders at any point in the chain, whether at vaccine storage, transit, or distribution, to confirm temperature stability. Given the recent IBM concerns that cold chain systems have already been targeted by hackers, track-and-trace technologies associated with strong cybersecurity measures are essential [90]. Vaccine manufacturing batches should be labeled and coded to make it possible to track quality problems and identify their root causes. Monitoring of inbound raw materials and outbound shipments has to be linked to a secure, traceable digital control cross-border system that is connected to national coordinators [96]. Stakeholders should assess and put into practice top industrial technologies for cybersecurity and digital health, including the IoT for smart sensors and distributed ledgers for supply chain tracking, monitoring [90], and communication [96]. This shall improve real-time vaccine data flow visibility and regular monitoring, which is crucial for increasing the efficiency of the vaccination supply chain [95].

The vaccine cold supply chain constitutes a complex network of interconnected participants, each occupying a distinct and non-interchangeable role. Manufacturers, together with raw material and adjuvant suppliers, initiate the chain and are responsible for product integrity at the point of origin. Logistics providers, including air, sea, and land transport partners, manage temperature-controlled movement between stages, representing critical transit nodes whose failure can propagate disruption downstream. Storage facilities, from national freezer centers to regional and local units, serve as intermediate holding nodes where temperature violations are particularly consequential given vaccine perishability. Healthcare centers represent terminal delivery nodes where vaccines are administered, and adverse effects are first recorded. Government agencies govern the network as a whole, overseeing customs, regulatory compliance, and coordination across all stages, rather than occupying a single point within the sequential flow. Research on multi-stakeholder supply chain resilience emphasizes that such systems require coordinated strategies, including information sharing, risk management, and technological integration, to withstand and recover from disruptions across their differentiated participants [97]. Complex systems, such as supply chains, are inherently vulnerable to disruptions capable of triggering cascading failures [98], and network science research shows that node influence in such systems is unevenly distributed. A minority of structurally dominant nodes exert disproportionate control over network function, and their targeted disruption causes disproportionate structural damage relative to their number [98,99]. Node-level analysis of supply chain resilience further confirms that different nodes exhibit distinct vulnerability profiles, with storage nodes particularly susceptible to infrastructure-related disruption and transit nodes more commonly affected through operational restrictions than direct physical damage [100]. These structural characteristics directly motivate the design of the proposed system, in which each supply chain stage is represented as a distinct accountability node with assigned and certified personnel, enabling granular traceability and forensic accountability at every point in the network.

## 4. The Developed Blockchain System for Beneficiaries’ Registration, Probable Side Effects and Vaccine Cold Supply Chain

This section demonstrates the rationale, detailed architecture, and operation of the developed blockchain-based system. Our system components are detailed as follows:

1. Ethereum blockchain: A distributed network that stores immutable and traceable records of information about system participants and their access restrictions, logs showing function operations, and events emitted by the smart contract.
2. Smart contracts: Two smart contracts operating together, which are the registration and side effects smart contract and the vaccine cold supply chain smart contract.
3. Interplanetary file system (IPFS): A decentralized storage system used for a large volume of data. A file stored in IPFS is considered secure and tamper-resistant, as the system generates a cryptographic hash that is unique to it, which represents an ideal way to verify the authenticity of data files [101]. Our system utilizes IPFS to save and hash vaccine beneficiaries’ photos and store their hash in the registration smart contract.
4. Vaccine trucks, storage facilities, and smart containers: These are equipped with IoT devices, such as temperature and light sensors. In our system design, we suggest using a smart container for each vaccine lot. This shall enable better traceability of the vaccine shipment throughout its supply chain journey.
5. IoT technology in the form of temperature sensors and light exposure sensors will monitor and trace the status of vaccine lots throughout the supply chain journey. Temperature sensors will send signals to the vaccine cold supply chain smart contract, giving the temperature value of the connected smart container, while light sensors will send signals in case of light exposure of the vaccine lot.
6. Blockchain oracles: Verify the data captured from temperature and light sensors and inject them into the cold supply chain smart contract. The notion of oracles is important in blockchain ecosystems as it is known that once data is added to the tamper- resistant blockchain, it cannot be changed or amended [102,103]. In our system design, a hardware inbound oracle is suggested.

Stakeholders interact with the smart contracts via specially designed front-end applications or websites that are connected to the smart contracts using Application Program Interfaces (APIs).

### 4.1. System Architectural Design Rationale

The design of a blockchain-based system for vaccine registration, side effect reporting, and cold chain monitoring requires a clear understanding of the specific trust problem the technology is intended to solve, particularly given the involvement of a central authority, such as the government, in coordinating stakeholders. This subsection outlines the rationale underlying the proposed architecture and clarifies the properties that distinguish it from a conventional database-driven approach.

Although the system administrator (in this case, the government) determines which stakeholders are admitted to the network, admission control alone does not represent the primary trust problem addressed by this architecture, as this could equally be achieved using a conventional permissioned database. The advantage of the proposed blockchain-based design instead arises from four properties not jointly present in a conventional database:

1. Distributed validation and absence of a single point of control. New records require agreement from multiple independent parties before being committed, rather than approval from a single administrator, so that no one stakeholder, including the government, can unilaterally control or manipulate the data, while a conventional database relies entirely on a single administering entity to approve, commit, and manage every entry, creating a single point of control and a single point of failure.
2. Tamper-evidence and immutability of data. Once a record is committed, it cannot be altered or deleted by any party, including the system administrator, due to cryptographic hash-chaining between blocks, and any attempt at retroactive modification is immediately detectable by all participants, while a conventional database allows the administrator to alter or delete historical records without independent detection, since audit trails in such systems are themselves under the administrator’s control.
3. Independent, trustless auditability. All stakeholders can independently verify the integrity of the shared record from their own systems, without needing to trust the honesty or competence of a central administrator, while a conventional database requires every stakeholder to trust the administrator’s own audit logs, which offer no independent mechanism of verification and are vulnerable to the same administrator who controls the underlying data.
4. Smart contract automation. The proposed system enforces business rules, such as cold chain temperature thresholds, through smart contracts, which combine four properties not jointly available in conventional application code: self-execution, triggering actions automatically once predefined conditions are met, without manual intervention by any party; immutability of logic, meaning the rules themselves, once deployed, cannot be secretly altered by any single stakeholder; transparency, since the same rules are visible to and verifiable by all participants; and determinism, ensuring identical inputs always produce identical outputs, applied consistently across every stakeholder. In contrast, a conventional database depends on administrator-controlled application code, which can be modified selectively or without notice, typically requires manual or intermediary intervention to trigger actions, and offers no guarantee that the same rule is executed consistently or transparently for every stakeholder.
5. End-to-end provenance across organizational boundaries. Every transaction, from beneficiary registration through cold chain temperature logging to side effect reporting, is recorded as an immutable, timestamped entry that can be traced across its full history by any authorized stakeholder, enabling rapid identification of the origin of a fault (e.g., a cold chain breach) without reconciling siloed records across multiple organizations, while a conventional database architecture typically distributes this information across separately administered systems belonging to different organizations, requiring manual reconciliation or trusted intermediaries to reconstruct a complete and reliable history.

The current implementation was developed and evaluated on Ethereum; however, these architectural properties are not specific to this platform and apply equally to permissioned blockchain platforms, such as Hyperledger Fabric, which is included for comparison in Table 3. The selection of a specific underlying platform for production deployment is therefore an implementation decision independent of the system’s core trust model.

### 4.2. Cold Supply Chain System Participants and Roles

The following demonstrates the blockchain system participants and their roles:

1. Government: The main participant that deploys both systems’ smart contracts and adds cold supply chain partners and health administrators.
2. Healthcare Administrators: These are mostly health authorities personnel, physicians, and nurses who are assigned by the government to register vaccine beneficiaries and record any witnessed vaccine side effects.
3. Chains: These form the vaccine cold supply chain, which involves the vaccine manufacturer, the logistics providers, and the government and its resources and facilities, such as the medical sector, airports, and freezer storage facilities. The vaccine cold supply chain consists of a manufacturer, a transportation provider from manufacturing facilities to the airport, the airplane while airborne, a logistics partner for transit to national storage, a national storage and distribution facility, a distributor to regional storage, a regional storage and distribution center, and a logistics partner to provide transportation to healthcare centers, hospitals, and healthcare centers that administer vaccines and follow up with beneficiaries for any witnessed side effects.

Each participant is given a unique Ethereum Address (EA) to interact with the system’s smart contracts. Also, participants are given restricted access to the related smart contract’s functions using modifiers. Also, they will be able to view any information recorded on the chain, including logs, transactions, and light and temperature limitations breaches. However, this is decided according to the participant’s role in the system.

### 4.3. System Processes Flow, Algorithms, and Message Sequencing

**Figure 3:**
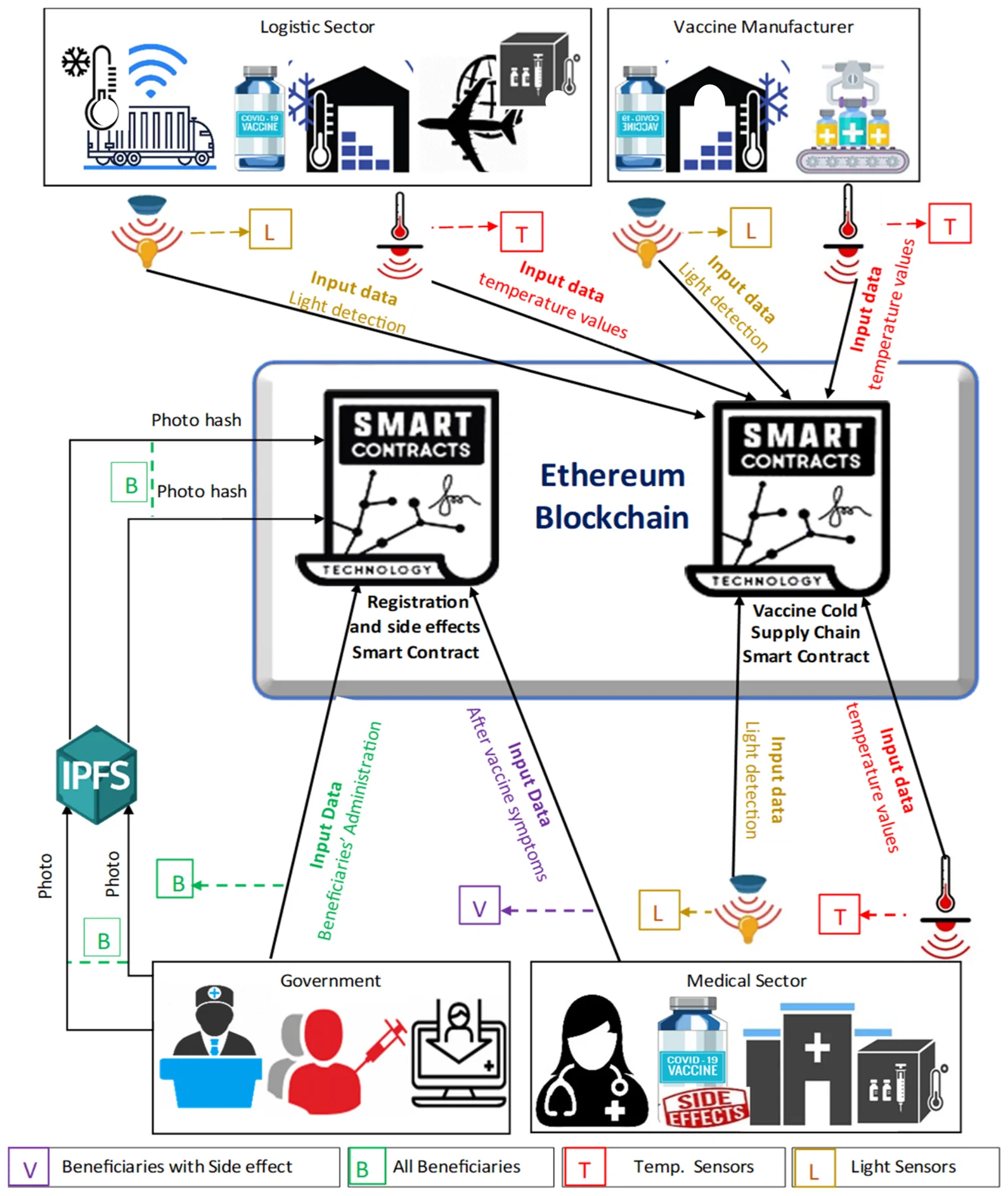
Blockchain-based vaccine system architecture.

Our research addresses important factors that are crucial to the success of vaccination programs in a manner that, to our knowledge, has never previously been addressed by other research. The design concept of our smart contract-based system, shown in Figure 3, supports the following:

1. Secured, validated, tamper-resistant, and transparent vaccine beneficiaries’ vaccination registration process.
2. Traceable, immutable, transparent, and reliable recording of probable vaccine beneficiaries’ side effects.
3. Comprehensive, traceable, transparent, secure, reliable, authenticated, and trusted vaccine cold supply chain monitoring and tracking, reinforced by dedicated oracle access control and sensor design measures, covering the entire network of manufacturing, storage, and distribution centers while adhering to safety and production limitations regarding temperature range and light exposure given by manufacturing companies or governments and ministries of health.
4. Linking all the above operations forms a comprehensive system of interconnected data, where any witnessed side effect is associated with the beneficiaries’ metadata and the vaccine lot supply chain journey information.

The importance of gathering vaccine side effect data in conjunction with beneficiaries’ registration records lies in its vital role in enhancing the vaccine production process and preparing the healthcare system to handle and treat vaccine recipients’ possible side effects. The developed system is initiated by the government, which first deploys the supply chain smart contract when it places a vaccine order from a specific vaccine manufacturer. At the deployment stage, the government adds its name and specifies the vaccine manufacturer and the upper and lower temperature limits for this vaccine type. The cold supply chain smart contract will capture the system initiation time and the EA of the government. It will also support complete traceability functionality for vaccine lots throughout their supply chain journey, as stated in Algorithm 1, where the government starts adding the chains of the vaccine cold supply chain to the system by inserting each chain’s functionality as specified by the smart contract code, as well as the chain’s EA and position number in the chain. The smart contract will automatically check the existence of the chain. If not, the smart contract will add the chain to *all chains* map and emit an event announcing the addition of the new chain to the system. The manufacturer then states the beginning of the vaccine production process through the smart contract, which specifies the location of the vaccine shipment at this stage of the cold supply chain to be at the manufacturer. The smart contract emits an event announcing that the shipment is now ready at the manufacturer’s storage premises. Additionally, the smart contract provides transparent traceability of the location of the vaccine shipment by supporting a vaccine location update functionality where each chain updates the location of the shipment whenever it receives it from the previous chain. The smart contract automatically checks that the shipment is moving according to the chain’s position number specified by the government earlier in the chain’s functionality flow. Also, the smart contract emits an event announcing any update in the vaccine shipment location.

Our cold supply chain smart contract also supports monitoring and tracking of temperature and light violations as described in Algorithms 2 and 3. It is known that each vaccine shipment consists of numerous vaccine lots, usually enclosed in smart containers, each of which is fitted with a temperature and a light sensor. This means that each vaccine lot is equipped with one temperature sensor and one light sensor. Each temperature sensor sends temperature readings and its sensor ID to the cold supply chain smart contract on a configurable periodic basis, with the submission interval determined by the deploying authority in accordance with applicable cold chain monitoring standards. WHO guidelines specify that electronic temperature loggers must record at intervals of 10 min or less during transit [104]. Each submitted reading constitutes one individual blockchain transaction. The smart contract automatically compares the received data to the coded rules about the upper and lower temperature limits that were specified by the government at the system deployment stage. If a temperature range breach occurs, the smart contract traces the location of the vaccine lot and emits an event announcing the occurrence of a violation and the value of the captured temperature. It also maps the sensor ID and the violation occurrence number to the temperature value, supply chain number, and time of violation, providing comprehensive, transparent, immutable, secure, traceable, trusted, and reliable data records about where and when the temperature violation occurred and the value of the recorded data. Data source integrity is enforced through smart contract-level oracle access control that restricts sensor data submission exclusively to the registered oracle address, as discussed in the system design features below.
**Algorithm 1:** Cold Supply Chain Traceability1:**State:**2:**Map** (enum *chainName*, address *chainAddress*) → *allChains*3:**Function:** 
*addChain*
4:**Input:** 
*Supply Chain Name, Supply Chain EA, Supply Chain No.*
5:**Modifier:** 
*onlyGov*
6:**Start**7:**Require** Chain EA ∉ stakeholdersList **Map**8:  **Mapping** Chain No., Chain Name, Chain EA → allChains **Map**9:  **Set** Chain EA10:  **Emit** NewChain (Chain Name, Chain EA, Chain No.)11:**End**12:**State:** Vaccine production order placed with manufacturer13:**Function** startProduction14:**Input:** -
15:**Modifier:** *onlyManufacturer*16:**Start**17:**Set** coldSupplyChain = manufacturer18:**Emit** VaccineLocation (“vaccine in production”, block.timestamp)19:**End**20:**Function** updateVaccineLocation21:**Input**: -22:**Modifier**: -23:**Start**24:**If** msg.sender = = transit_from_manufacturer_to_airport EA **THEN**25:  **Require** coldSupplyChain = = manufacturer26:  **Set** coldSupplyChain = transit_from_manufacturer_to_airport27:  **Emit** VaccineLocation (“vaccine in transit from manufacturer to airport”, block.timestamp)28:**Else If** msg.sender = = Aircraft_Airborne EA **THEN**29:  **Require** coldSupplyChain = = transit_from_manufacturer_to_airport30:  **Set** coldSupplyChain = Aircraft_Airborne31:  **Emit** VaccineLocation (“vaccine in aircraft”, block.timestamp)32:**Else If** msg.sender = = transit_from_ airport _to_ national storage EA **THEN**33:  **Require** coldSupplyChain = = Aircraft_Airborne34:  **Set** coldSupplyChain = transit_from_ airpor _to_nationalStorage35:  **Emit** VaccineLocation (“vaccine in transit from airport to national storage”, block.timestamp)36:**Else If** msg.sender = = National_Storage EA **THEN**37:  **Require** coldSupplyChain = = transit_from_ airport _to_nationalStorage38:  **Set** coldSupplyChain = National_Storage39:  **Emit** VaccineLocation (“vaccine in national storage”, block.timestamp)40:**Else If** msg.sender = = transit_from_national_to_ regional storage EA **THEN**41:  **Require** coldSupplyChain = = National_Storage42:  **Set** coldSupplyChain = transit_from_national_to_regional storage43: **Emit** VaccineLocation (“vaccine in transit from national to regionalstorage”, block.timestamp)44:**Else If** msg.sender = = Regional_ Storage EA **THEN**45:  **Require** coldSupplyChain = = transit_from_national_to_regional storage46:  **Set** coldSupplyChain = Regional_ Storage47:  **Emit** VaccineLocation (“vaccine in regional storage”, block.timestamp)48:**Else If** msg.sender = = transit_from_Regional_to _Medical center EA **THEN**49:  **Require** coldSupplyChain = = Regional_ Storage50:  **Set** coldSupplyChain = transit_from_Regional _to_Medical center51:  **Emit** VaccineLocation (“vaccine in transit from regional to medical center”, block.timestamp)52:**Else If** msg.sender = = Medical_Center EA **THEN**53:  **Require** coldSupplyChain = = transit_from_Regional_to_Medical center54:  **Set** coldSupplyChain = Medical_Center55:  **Emit** VaccineLocation (“vaccine in Medical_Center”, block.timestamp)56:**End**

Also, each light sensor sends a signal to the smart contract in case of any light exposure, providing the light sensor ID and stating the occurrence of a light exposure violation. The smart contract traces the location of the vaccine lot and emits an event announcing the occurrence of a light exposure violation. It also maps the sensor ID and the violation occurrence number to the supply chain number and the time of violation, providing complete, transparent, immutable, secure, traceable, trusted, and reliable information about where and when the light violation occurred. The integrity of the submitted sensor data is maintained through smart contract access control mechanisms that permit light sensor data submission exclusively from the designated oracle address, as elaborated in the system design discussion below. This collected information is extremely valuable for system stakeholders to benefit from in decision making, analysis and planning, manufacturing, supply chain management, and healthcare services improvement.

As Algorithms 2 and 3 show, only readings confirmed as violations by the smart contract are stored on-chain in the *ViolationsVaccineLotTemp* and *vaccineLotLightRecord* mappings, respectively; normal in-range temperature readings are not stored as records. Each sensor reading is submitted as an individual transaction without batching, preserving the precise timestamp of every submission, which is essential for determining the exact stage and time at which a violation occurred. The number of blockchain transactions per vaccine lot is therefore proportional to the configured submission frequency and the duration of the supply chain journey. While on-chain storage requirements are modest given that only violation records are stored permanently, the volume of sensor-originated transactions at high submission frequencies is a practical consideration for public blockchain deployments. For such deployments, Layer-2 rollup solutions could be considered, as they provide substantially reduced transaction costs while preserving blockchain security guarantees. For national government implementations, a permissioned blockchain architecture operated by authorized supply chain stakeholders could be considered, as it eliminates transaction cost constraints while maintaining the immutability and auditability that are central to the system traceability objectives.

Several design features of the proposed system address the reliability and integrity of IoT data entering the blockchain. Each vaccine lot is equipped with one dedicated temperature sensor and one light sensor, ensuring that conflicting concurrent readings from multiple sensors for the same lot cannot arise. The *tempData* and *lightExposure* functions in the smart contract include an explicit access control check, requiring that the calling Ethereum address is not a registered system stakeholder, implemented through the *stakeholdersList* mapping. This means that administrators, supply chain operators, and any other registered participant are automatically prevented from submitting sensor readings, ensuring that only the designated oracle address can call these functions. This design deliberately separates the roles of system administration and sensor data submission, preventing incorrect manual data entry through the smart contract interface. In the event of sensor malfunction or loss of connectivity between the sensor and the oracle, readings are not submitted to the smart contract during the affected period. This represents a safer failure mode from a data integrity perspective: no false violations are recorded, and no false accountability attributions are generated. However, the absence of readings during such a period means that temperature conditions cannot be verified from blockchain records for that interval, which is a consideration for production deployment addressed through operational protocols, such as redundant sensor configurations and mandatory sensor status verification, at each supply chain handover point. The smart contract independently evaluates each submitted reading against the configured temperature thresholds set by the government at deployment, ensuring that the violation determination is made by the tamper-resistant smart contract logic rather than delegated to the external oracle. With respect to the risk of hacker manipulation of sensor data or a breach of the oracle, the primary software-level mitigation is that only the registered oracle Ethereum address may call the sensor data submission functions, requiring an attacker to compromise the oracle’s cryptographic private key. Hardware-level oracle security, including trusted execution environments and hardware security modules, is identified as an important engineering direction for production deployments. The cold chain violation records produced by this system identify the personnel assigned to each supply chain stage during which a violation was recorded; they do not constitute automatic accountability determinations. Any accountability assessment requires human investigation, using the blockchain record as a verifiable, tamper-resistant data trail, consistent with the role of data-recording systems across other safety-critical domains.
**Algorithm 2:** Vaccine Temperature Monitoring Throughout Cold Supplychain1:**State:**2:**Map** (string *sensorID*, (string *violationsCount* → uint256 *time*, uint256 *temp*, enum *chain*)) → *VioltionsVaccineLotTemp*3:**Function:** *tempData*4:**Input:** *Temp reading*, *Sensor ID*5:**Start**6:**If** coldSupplyChain == manufacturer **THEN**7: **If** temperature >= upper threshold || temperature <= lower threshold **THEN**8: **Mapping** (sensor ID, (violationsCount → block.timestamp, temp, violations at manufacturer)) →  *ViolationsVaccineLotTemp*
**Map**9: **Emit** Temperature violation(“temperature is out of acceptable range”, temp)10:**Else If** coldSupplyChain = transit from manufacturer to airport **THEN**11:  **If** temperature >= upper threshold || temperature <= lower threshold **THEN**12:  **Mapping** (sensor ID, (violationsCount → block.timestamp, temp, violations at transit from   manufacturer to airport)) → *ViolationsVaccineLotTemp*
**Map**13:  **Emit** Temperature violation(“temperature is out of acceptable range”, temp) 14:**If** coldSupplyChain == Aircraft_Airborne **THEN**15:  **If** temperature >= upper threshold || temperature <= lower threshold **THEN**16:  **Mapping** (sensor ID, (violationsCount → block.timestamp, temp, violations a Aircraft_    Airborne)) → *ViolationsVaccineLotTemp*
**Map**17:  **Emit** Temperature violation(“temperature is out of acceptable range”, temp) 18:**If** coldSupplyChain == transit_from_ airport _to_national storage **THEN**19:  **If** temperature >= upper threshold || temperature <= lower threshold **THEN**20:  **Mapping** (sensor ID, (violationsCount → block.timestamp, temp, violations at transit _from     _ airport _to_national storage)) → *ViolationsVaccineLotTemp*
**Map**21:  **Emit** Temperature violation(“temperature is out of     acceptable range”, temp) 22:**If** coldSupplyChain == National_Storage **THEN**23:  **If** temperature >= upper threshold || temperature <= lower threshold **THEN**24:  **Mapping** (sensor ID, (violationsCount → block.timestamp, temp, violations at National    _Storage)) → *ViolationsVaccineLotTemp*
**Map**25:  **Emit** Temperature violation(“temperature is out of acceptable range”, temp) 26:**If** coldSupplyChain == transit_from_national_to_regional storage **THEN**27:  **If** temperature >= upper threshold || temperature <= lower threshold **THEN**28:  **Mapping** (sensor ID, (violationsCount → block.timestamp, temp, violations at transit_    from_national_to_regional storage)) → *ViolationsVaccineLotTemp*
**Map**29:  **Emit** Temperature violation(“temperature is out of acceptable range”, temp) 30:**If** coldSupplyChain == Regional_ Storage **THEN**31:  **If** temperature >= upper threshold || temperature <= lower threshold **THEN**32:  **Mapping** (sensor ID, (violationsCount → block.timestamp, temp, violations at Regional_    Storage)) → *ViolationsVaccineLotTemp*
**Map**33:  **Emit** Temperature violation(“temperature is out of acceptable range”, temp) 34:**If** coldSupplyChain == transit_from_Regional_to_ Medical centre **THEN**35:  **If** temperature >= upper threshold || temperature <= lower threshold **THEN**36:  **Mapping** (sensor ID, (violationsCount → block.timestamp, temp, violations transit_from    _Regional_to_Medical centre)) → *ViolationsVaccineLotTemp*
**Map**37:  **Emit** Temperature violation(“temperature is out of acceptable range”, temp) 38:**If** coldSupplyChain == Medical_Center **THEN**39:  **If** temperature >= upper threshold || temperature <= lower threshold **THEN**40:  **Mapping** (sensor ID, (violationsCount →block.timestamp, temp, violations at Medical _Center))    → *ViolationsVaccineLotTemp*
**Map**41:  **Emit** Temperature violation(“temperature is out of acceptable range”, temp) 42:**End**

**Algorithm 3:** Vaccine Light Exposure Monitoring Throughout Cold Supplychain1:
**State:**
2:**Map** (string *lightSensorID*, (string *violationsCount* → uint256 *time*, enum *chain*)) → *vaccineLotLightRecord*3:**Function:** 
*lightExposure*
4:**Input:** 
*lightViolationSignal, lightSensor ID*
5:
**Start**
6:**If** coldSupplyChain == manufacturer **THEN**7:  **Mapping** (sensor ID, (violationsCount → *true*, block.timestamp, violations at manufacturer)) →   *vaccineLotLightRecord*
**Map**8:  **Emit** Lightviolation(“Light Exposure Detected”)9:**Else If** coldSupplyChain = transit from manufacturer to airport **THEN**10: **Mapping** (sensor ID, (violationsCount → *true*, block.timestamp, violations at transit from   manufacturer to airport)) → *vaccineLotLightRecord*
**Map**11: **Emit** Lightviolation(“Light Exposure Detected”)12:**If** coldSupplyChain == Aircraft_Airborne **THEN**13: **Mapping** (sensor ID, (violationsCount → *true*, block.timestamp, violations at Aircraft_ Airborne))   → *vaccineLotLightRecord*
**Map**14: **Emit** Lightviolation(“Light Exposure Detected”)15:**If** coldSupplyChain == transit_from_ airport_to_national storage **THEN**16: **Mapping** (sensor ID, (violationsCount → *true*, block.timestamp, violations at transit_from _   airport _to_national storage)) → *vaccineLotLightRecord*
**Map**17: **Emit** Lightviolation(“Light Exposure Detected”) 18:**If** coldSupplyChain == National_Storage **THEN**19: **Mapping** (sensor ID, (violationsCount → *true*, block.timestamp, violations at National_Storage))   → *vaccineLotLightRecord*
**Map**20: **Emit** Lightviolation(“Light Exposure Detected”)21:**If** coldSupplyChain == transit_from _national_to_regional storage **THEN**22: **Mapping** (sensor ID, (violationsCount → *true*, block.timestamp, violations at   transit_from_national_to_regional storage)) → *vaccineLotLightRecord*
**Map**23: **Emit** Lightviolation(“Light Exposure Detected”)24:**If** coldSupplyChain == Regional_ Storage **THEN**25: **Mapping** (sensor ID, (violationsCount → *true*, block.timestamp, violations at Regional_Storage))   → *vaccineLotLightRecord*
**Map**26: **Emit** Lightviolation(“Light Exposure Detected”)27:**If** coldSupplyChain == transit_from_Regional _to _Medical centre **THEN**28: **Mapping** (sensor ID, (violationsCount → *true*, block.timestamp, violations at transit_from _   Regional_to_Medical centre)) → *vaccineLotLightRecord*
**Map**29: **Emit** Lightviolation(“Light Exposure Detected”)30:**If** coldSupplyChain == Medical_Center **THEN**31: **Mapping** (sensor ID, (violationsCount → *true*, block.timestamp, violations at Medical_ Center))   → *vaccineLotLightRecord*
**Map**32:  **Emit** Lightviolation(“Light Exposure Detected”)33:
**End**


Once the vaccine lots arrive at medical centers for beneficiaries’ inoculation, the government deploys the second smart contract dedicated to beneficiaries’ administration and side effects recording. At the deployment stage, the smart contract captures the deployment date and the name and EA of the initiating government. It also adds the government to the administrators’ list as the first administrator. The smart contract facilitates adding health administrators, who are responsible for registering the beneficiaries at the time of vaccine administration and for recording any possible side effects witnessed by vaccine recipients. The function of adding new health administrators is performed strictly by an existing administrator. The government account can also revoke any administrator’s access at any time using the *revokeAdmin* function, which immediately removes the administrator’s address from the *adminList* mapping, preventing any further write operations by that address while preserving all past transactions as an immutable accountability record. Algorithm 4 describes two functions responsible for beneficiary registration and multi-dose vaccination recording. Function 1 registers a new beneficiary profile and is called once per beneficiary. Function 2 records a vaccination event and is called once per dose, supporting primary series and booster doses.

- Function 1: *registerBeneficiaryProfile*; the administrator inputs the following beneficiary profile data (captured once):
  - Name;
  - ID Number;
  - Mobile Number;
  - Picture hash;
  - Date of birth;
  - Gender;
  - Marital Status.
- Function: *recordVaccinationEvent*; the administrator inputs the following vaccination event data (captured once per dose):
  - ID Number;
  - Dose number;
  - Vaccine type;
  - Vaccine lot number.

**Algorithm 4:** Beneficiaries Registration and Multi-Dose Vaccination Recording 
**Function 1:** *registerBeneficiaryProfile* (Called once per beneficiary) **State:** Struct *BeneficiaryProfile (string name, uint256 idNumber, uint256 mobileNumber, string picHash, uint256 DOB, enum gender, enum maritalStatus)* **Map:** (string *idNumber* → *BeneficiaryProfile*) → *beneficiaryProfiles* **Map:** (string *idNumber* → *bool*) → *profileExists*
**Function:** *registerBeneficiaryProfile*
**Input:**
*Beneficiary’s name, ID no., mobile no., photo hash, DOB, gender, marital status* **Modifier:**
*onlyAdmin* **Start**1:**Require** beneficiary’s ID no. ∉ profileExists **Map**2:**Mapping**: name,_idNumber, _mobileNumber,_picHash,_DOB,_gender, _maritalStatus → *beneficiaryProfiles* [idNumber] **Map**3:Set *profileExists* [idNumber] = true4:
**End**

**Function 2:** *recordVaccinationEvent* (Called once per dose)
**State**: Struct *VaccinationEvent* (uint256 *vaccinationTime*, string *vaccineLotNumber*, uint256 *doseNumber*, bool *sideEffectReported*) **Map**: (string *idNumber* → (uint256 *doseNumber* → *VaccinationEvent*)) → *vaccinationEvents*
**Function**: *recordVaccinationEvent* **Input**: *idNumber, doseNumber, vaccineType, vaccineLotNumber* **Modifier**: *onlyAdmin* **Start**1:**Require** idNumber ∈ profileExists Map2:**Require** *vaccinationEvents*[idNumber][doseNumber] does not exist3:**Mapping** block.timestamp, vaccineLotNumber, doseNumber, false → vaccinationEvents[idNumber][doseNumber]4:
**End**


For Function 1, the smart contract automatically captures the registration timestamp and stores all profile data in the *beneficiaryProfiles* mapping keyed on the beneficiary national ID number. For Function 2, the smart contract automatically captures the administration timestamp and the address of the administering health administrator, and stores the vaccination event data in the *vaccinationEvents* mapping keyed on the beneficiary national ID and dose number. The vaccine lot number stored in each vaccination event record enables direct linkage between any reported side effect and the specific lot administered, supporting full traceability through the cold supply chain records. A very important part of our proposed system is the functionality of recording any witnessed vaccine side effects shown in Algorithm 5. The smart contract’s register side effects function is strictly accessible by health administrators. When an administrator observes a side effect in any patient and, through diagnosis records, determines that it is potentially associated with vaccination, he/she can add the required data to the smart contract to form a record of the witnessed side effects, which are as follows:

- ID Number;
- Dose Number;
- Side Effect Name;
- Chronic Disease;
- Special Need.

In cases where an incorrectly recorded side effect needs to be corrected, the *amendSideEffect* function allows an authorized administrator to add a correction record referencing the original, preserving the original record on-chain while adding the amendment as a new traceable entry with its own timestamp.
**Algorithm 5:** Beneficiary’s side effects
**State:** **Map**: (uint256 *sideEffectCaseCounter* → string *idNumber*, uint256 *doseNumber*, string *vaccineLotNumber*, string *sideEffectName*, string *chronicalDisease*, string *specialNeed*, uint256 *sideEffectsTime*) → *sideEffectCases* **Map:** (string *idNumber* → (uint256 *doseNumber* → string *idNumber*, uint256 *doseNumber*, string *vaccineLotNumber*, string *sideEffectName*, string *chronicalDisease*, string *specialNeed*, uint256 *sideEffectsTime*)) → *sideEffectByBenAndDose*
**Function:** *registerSideEffects* **Input:**
*Beneficiary’s ID no., dose number, observed side effect, chronic diseases, special needs* **Modifier:**
*onlyAdmin* **Start**1: **Require** beneficiary’s ID no. ∈ *profileExists* **Map**2:**Require** *vaccinationEvents*[idNumber][doseNumber] exists Retrieve vaccineLotNumber from *vaccinationEvents*[idNumber][doseNumber] Set *vaccinationEvents*[idNumber][doseNumber].sideEffectReported = true3:**Mapping** sideEffectCaseCounter, idNumber, doseNumber, vaccineLotNumber, sideEffectName, chronicalDisease, specialNeed, block.timestamp → *sideEffectCases* **Map**
4:**Mapping** idNumber, doseNumber → VaccineEffects(idNumber, doseNumber, vaccineLotNumber, sideEffectName, chronicalDisease, specialNeed, block.timestamp) → *sideEffectByBenAndDose* **Map**5:**End**

The smart contract automatically captures the time of witnessing the side effects and retrieves the vaccine lot number directly from the *vaccinationEvents* mapping for the *specific dose recorded*, linking the side effect record to the exact vaccine lot administered. The *sideEffectCases* and *sideEffectByBenAndDose* mappings are updated with the complete side effect record including the dose number and vaccine lot number. The vaccine lot number is then used to trace the complete cold chain violation history for that lot using the *ViolationsVaccineLotTemp* and *vaccineLotLightRecord* maps in the supply chain smart contract.

## 5. The Developed Blockchain Beneficiaries’ Registration, Side Effects and Vaccine Cold Supply Chain System Implementation and Testing

**Figure 4:**
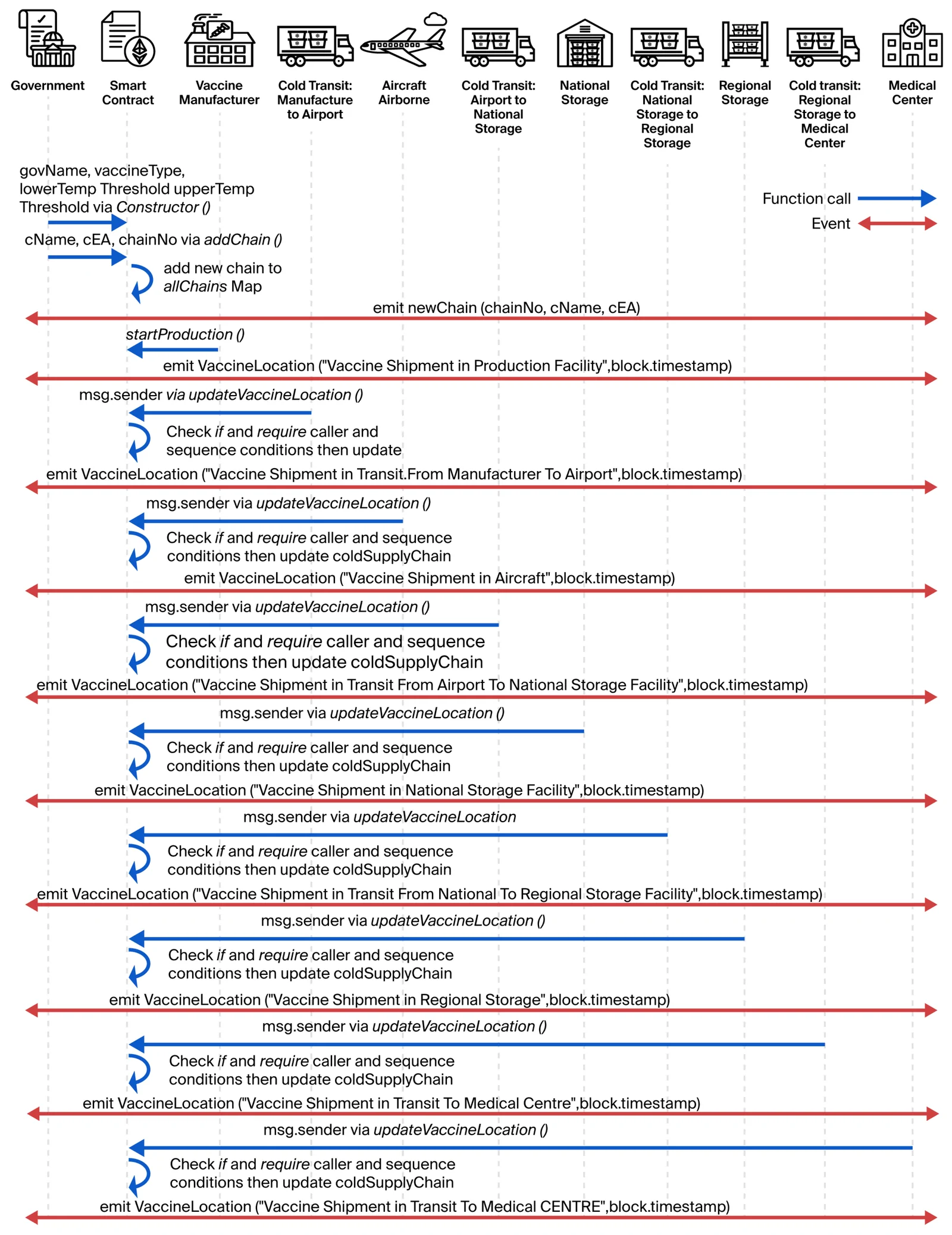
Message sequencing for the deployment and adding chains of the cold supply chain smart contract.

**Figure 5:**
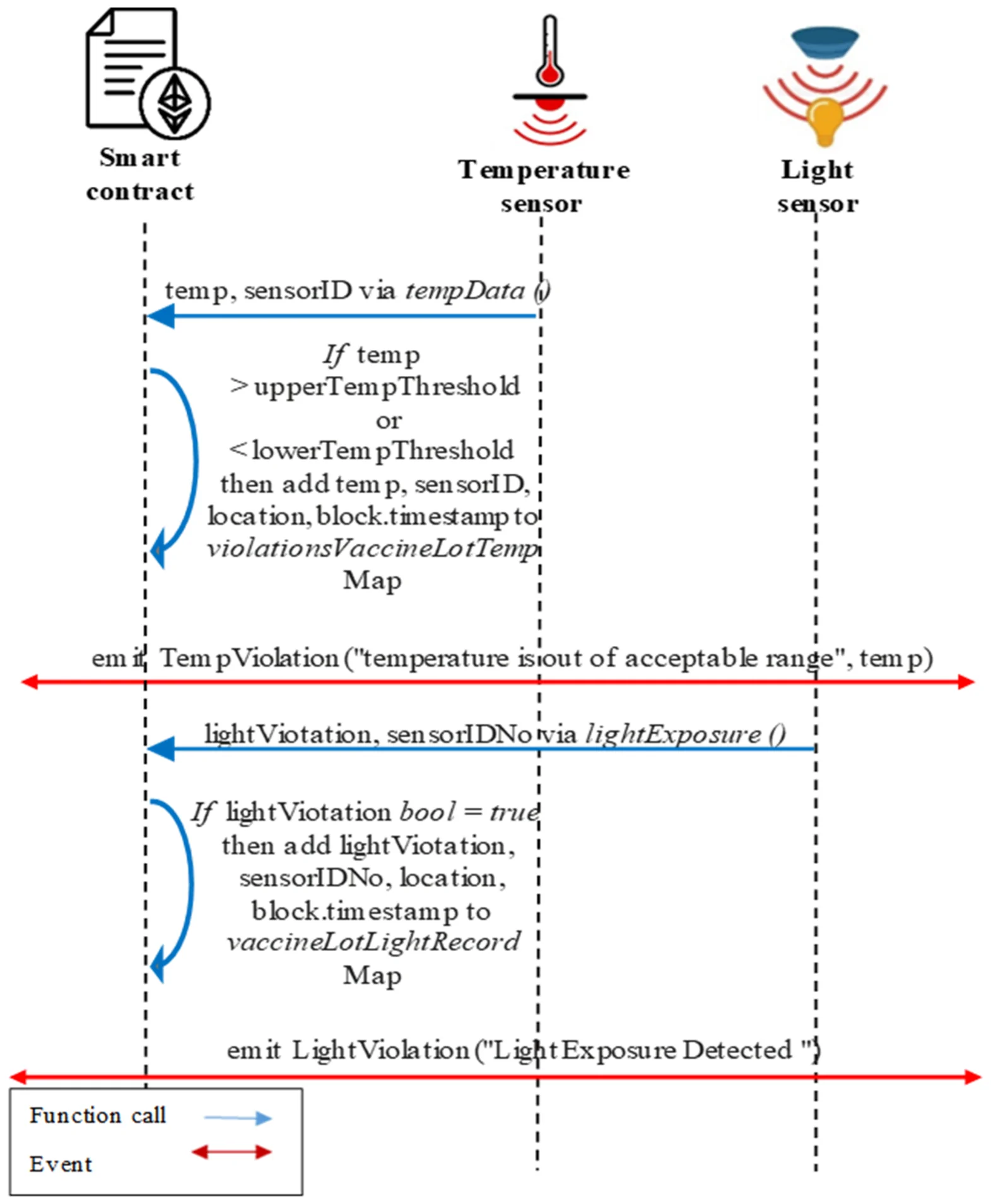
Message sequencing for temperature and light violation recordings of the cold supply chain smart contract.

The smart contract development and testing environment was the Remix IDE, which also supports debugging features. The testing process started with the cold supply chain smart contract. Figure 4 shows the message sequencing for deployment and adding chains, while Figure 5 shows the message sequencing of temperature and light sensor violation recording.

The smart contract was successfully deployed by the government, which was assumed to be the U.A.E., and given the EA of

“0x5B38Da6a701c568545dCfcB03FcB875f56beddC4”.

Other input parameters were assumed to have the following values and were inserted via the *constructor*: vaccineType = “Astrazeneca”, lowerTempThreshold = 2, upperTempThreshold = 8.

The government started adding the supply chains as previously described in Section 4 and coded in the smart contract as an *enums*. During the testing process, the government successfully used the function *addChain* to insert all supply chains into the system. The following parameters were inserted by the government for each chain, along with their EAs and position numbers in the supply chain:

*enum*: Chain Name;

*address*: Chain EA;

*uint256*: Chain No.

**Figure 6:**
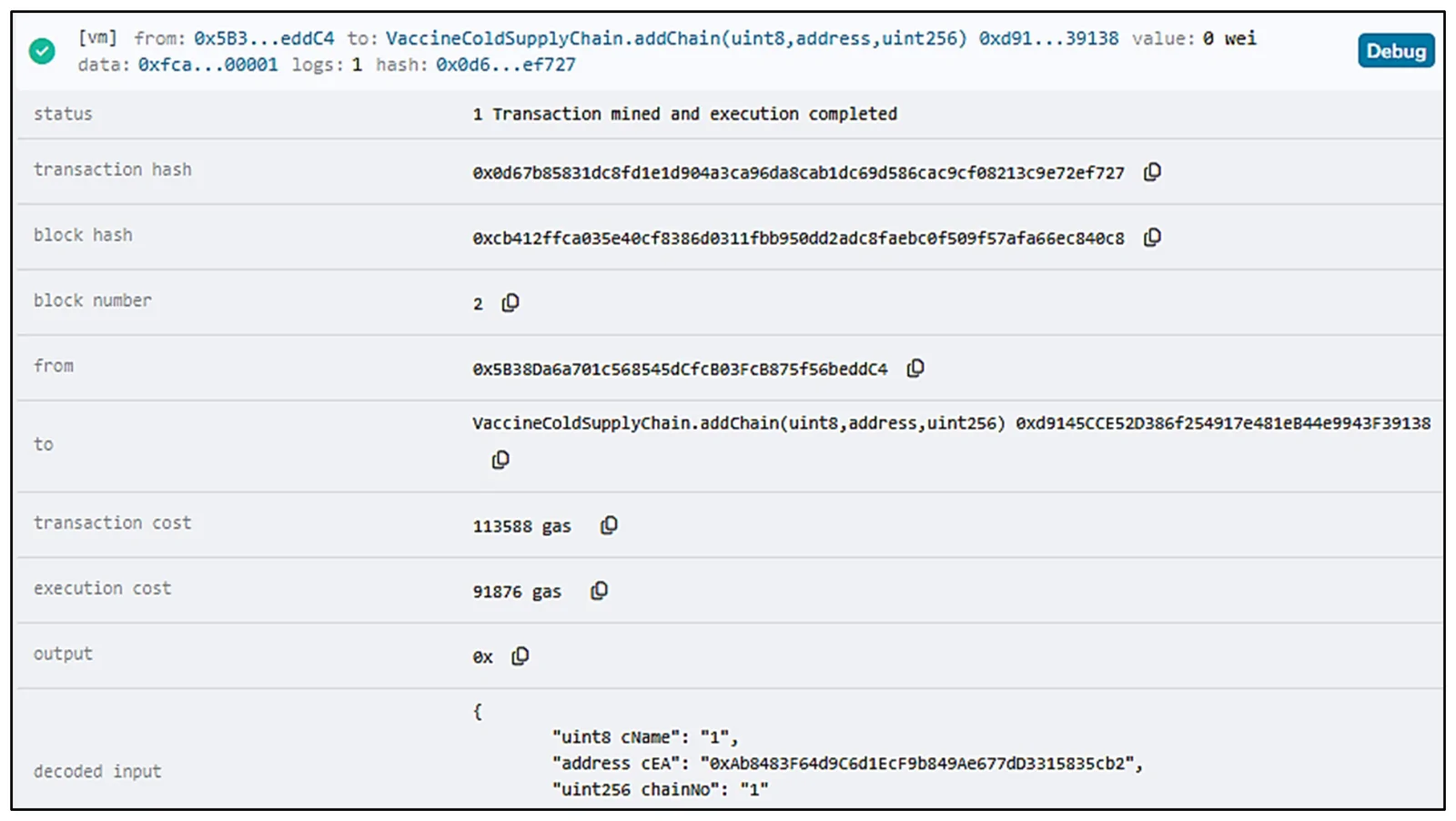
The successful addition of the first cold supply chain manufacturer to the system.

**Figure 7:**
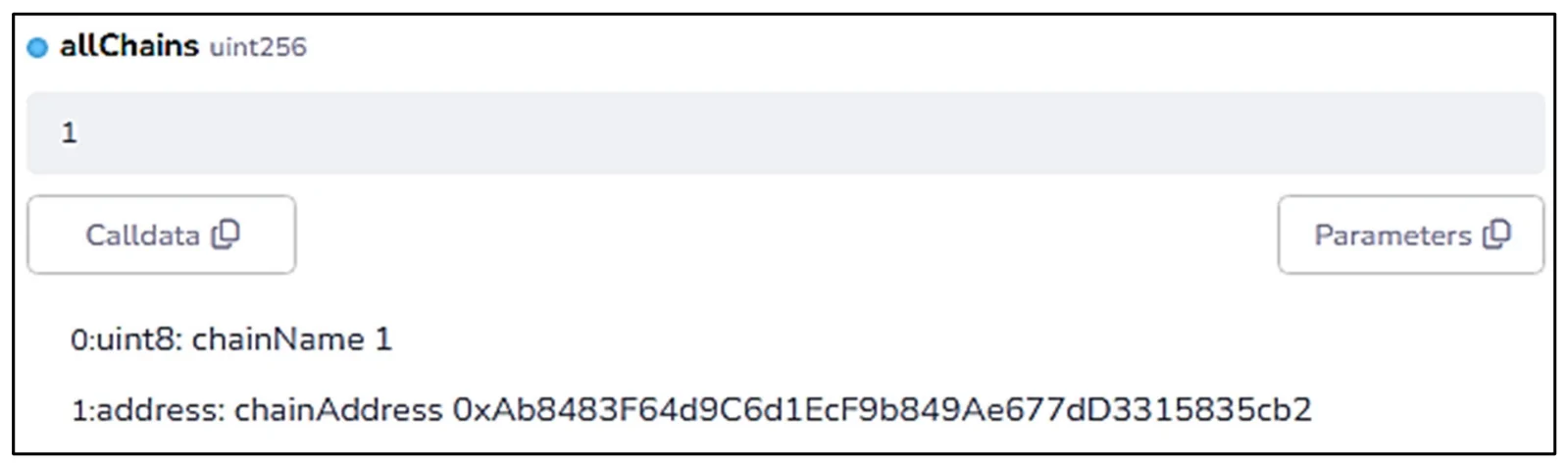
The coldsupplychain enum update to “1”.

**Figure 8:**
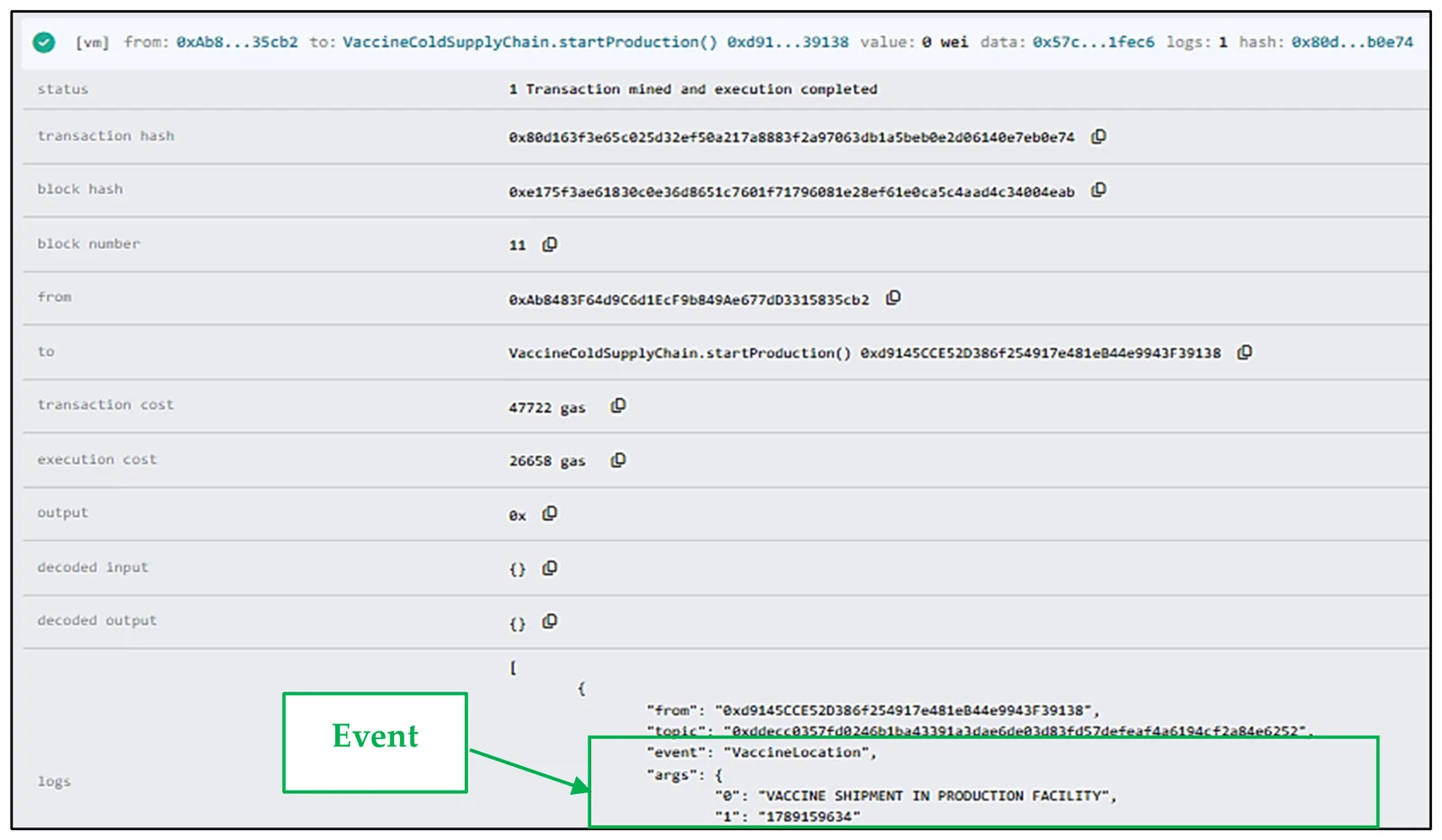
Successful smart contract execution of *startProduction* function by the manufacturer.

**Figure 9:**
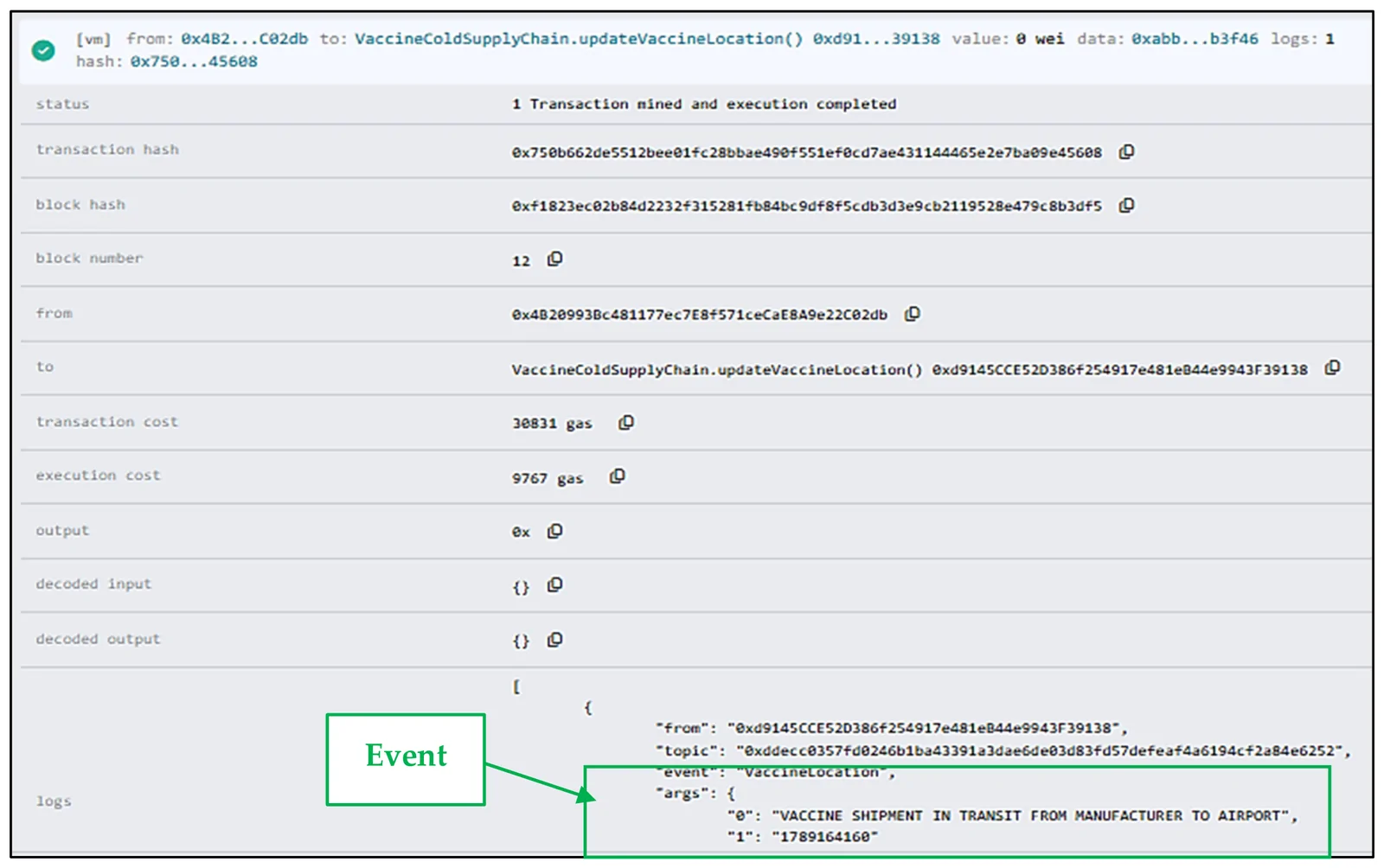
Successful smart contract execution of *updateVaccineLocation* function by the logistics provider.

Figure 6 demonstrates the successful addition of the first chain manufacturer. The rest of the chains were successfully added to the system and given their EAs and chain numbers in the supply chain, per Table 4. Continuing with the testing process, the manufacturing company used the function *startProduction* to announce the event “VACCINE SHIPMENT IN PRODUCTION FACILITY,” which declares the time using *block.timestamp*. The *coldsupplychain enum* was updated to “1”, as shown in Figure 7, stating the location of the vaccine shipment to be in the first chain. Also, the successful start of the supply chain journey is shown in Figure 8. Our cold supply chain smart contract supports the function *updateVaccineLocation*, which is used by each chain at the time of receiving the vaccine shipment. This function provides the required traceability in a secure manner. It also ensures the correct path of the vaccine shipment, as it requires each chain to receive the shipment from the previous chain in the correct order. Additionally, it supports transparency by updating the *coldsupplychain enum* and emitting the *VaccineLocation* event, declaring the new location of the vaccine shipment along with the time of location update. Figure 9 shows the successful execution of the *updateVaccineLocation* when the vaccine was received by the logistics provider to transfer it from manufacturing facilities to the airport. The *coldsupplychain enum* was updated to “2,” reflecting the vaccine shipment location.

In our testing scenario, the vaccine shipment continued to be transported to its destination and was moved to the aircraft. The smart contract emitted the event “VACCINE SHIPMENT IN AIRCRAFT” associated with the time of location change. It was assumed that at this stage of the cold supply chain, a vaccine lot with the number T321 suffered from temperature fluctuations, where the mounted sensor (which has the same ID no. of T321) recorded a temperature of 10 °C. In this case, the sensor uses the *tempData* function to insert the captured temperature value along with the sensor ID into the smart contract.

**Figure 10:**
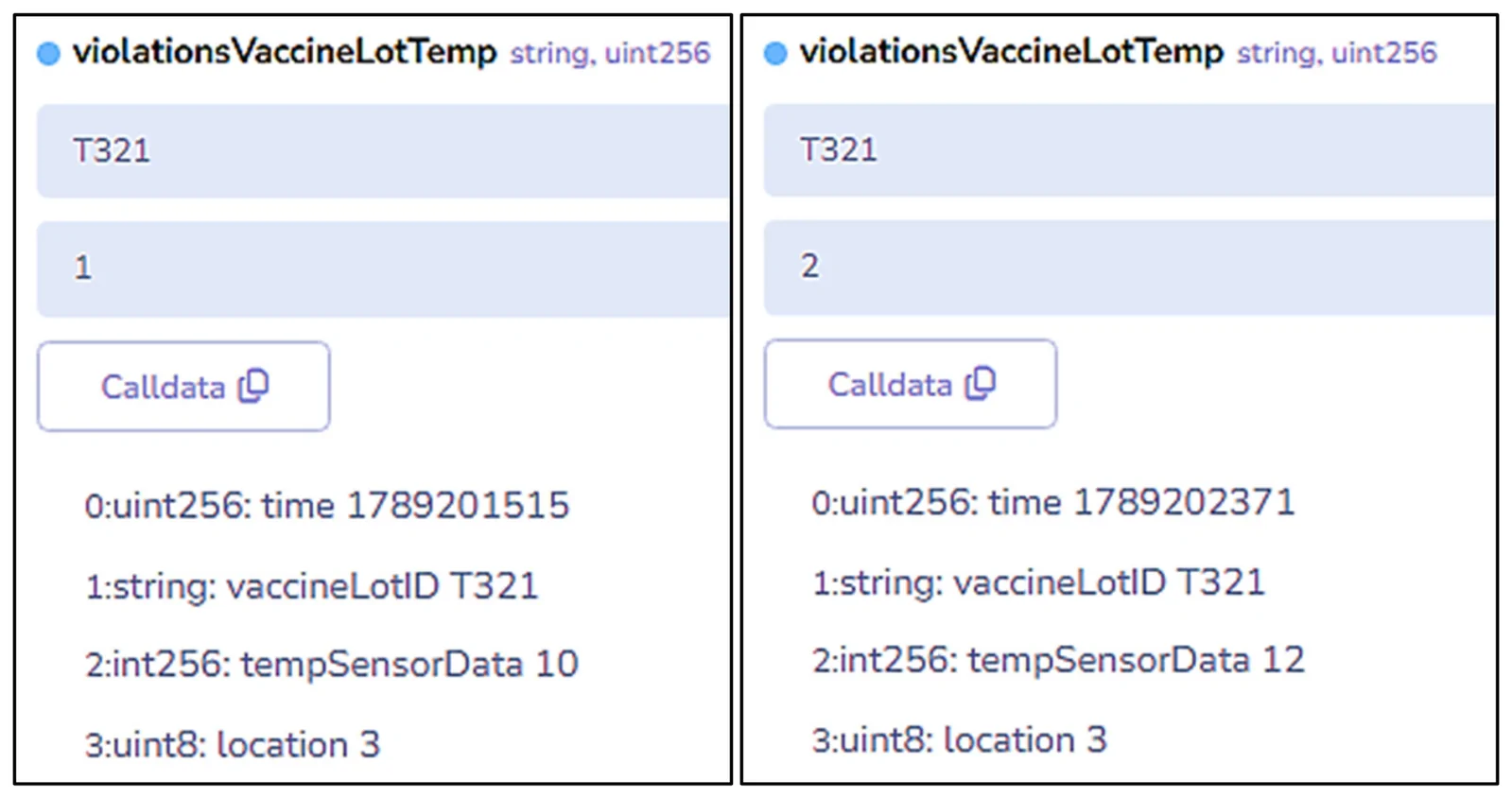
*violationsVaccineLotTemp*: Map of the first and second recorded temperature violations.

Also, the platform successfully executed the function *tempData* and invoked the event *TempViolation*, which emits the warning “*temperature is out of acceptable range*” along with the captured temperature value. For further testing, another temperature violation for the same vaccine lot was detected and inserted into the system. Figure 10 demonstrates the mapping of the sensor ID with the number of recorded violations and the time, location, and value of the two violations in the *violationsVaccineLotTemp Map*.

**Figure 11:**
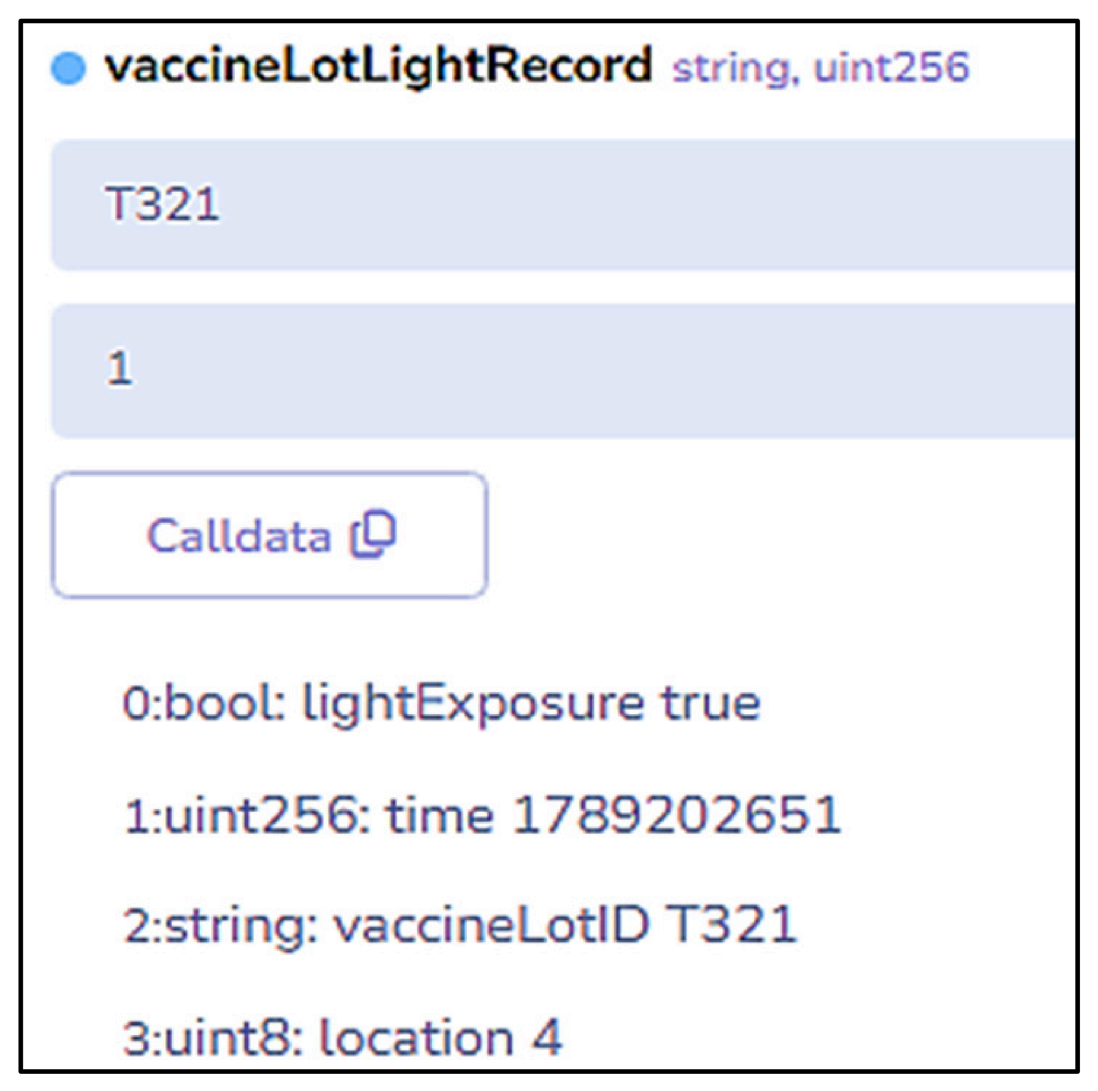
*ListViolateLightSensor:* Map of the recorded light violation.

The light-detection functionality was also tested. It was assumed that the same vaccine lot was exposed to light while it was in transit from the airport to the national storage facility. The light sensor inserted the *boolen* expression *true* into the smart contract along with the sensor ID using the function *lightExposure* and the invocation of the event *LightViolation*, which emits the warning “*Light Exposure Detected*”. Figure 11 shows the mapping of the sensor ID with the number of recorded light violations and the time and location in the *ListViolateLightSensor Map*.

**Figure 12:**
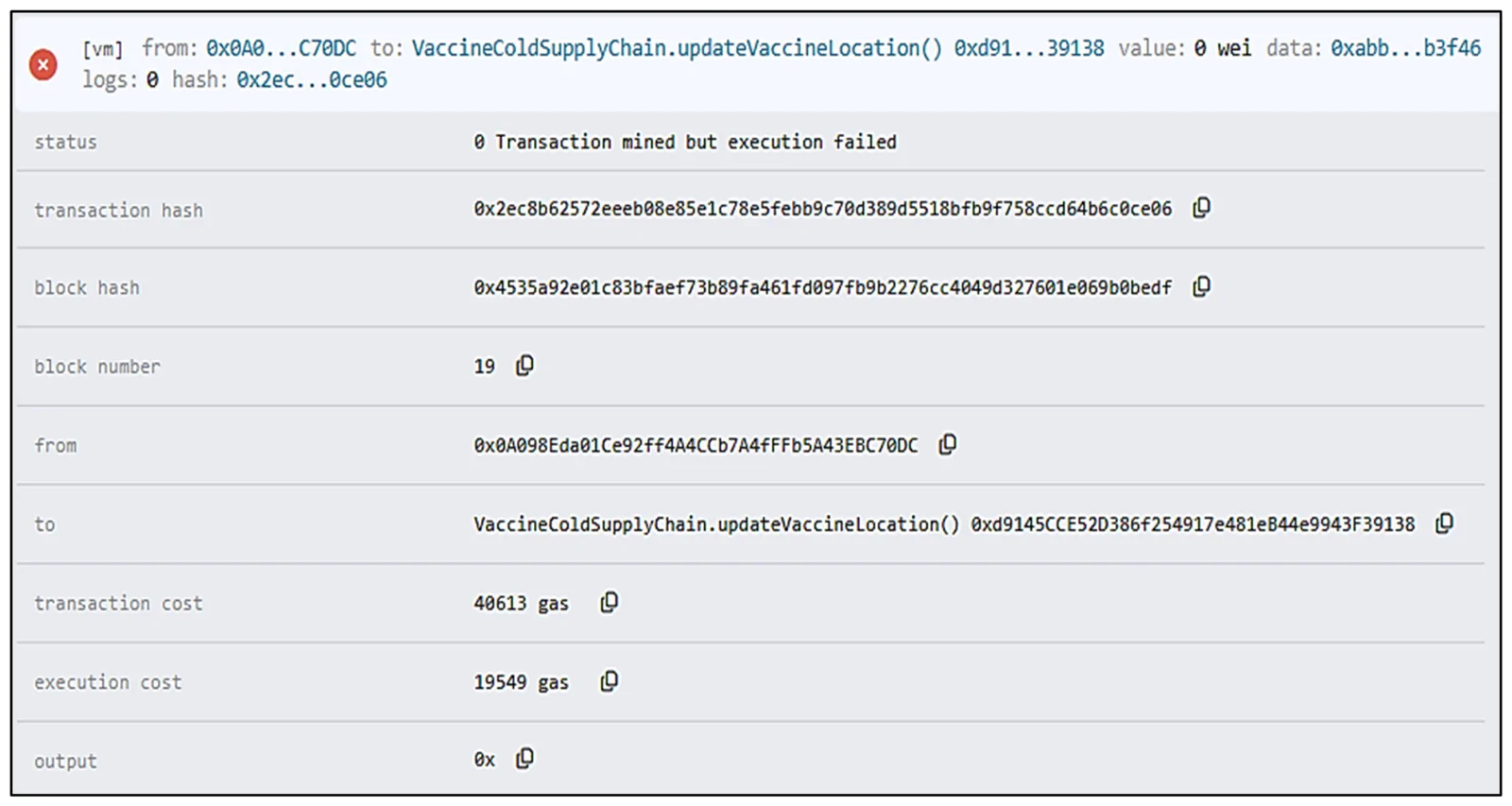
Smart contract didn’t execute the *updateVaccineLocation* function when called by the wrong chain.

The smart contract successfully traced the vaccine shipment throughout its cold supply chain journey to the ninth chain when it reached the medical center. Finally, the system was successfully tested against correct shipment handling, where it did not execute the *updateVaccineLocation* function unless the shipment was received from the correct preceding chain, as shown in Figure 12.

**Figure 13:**
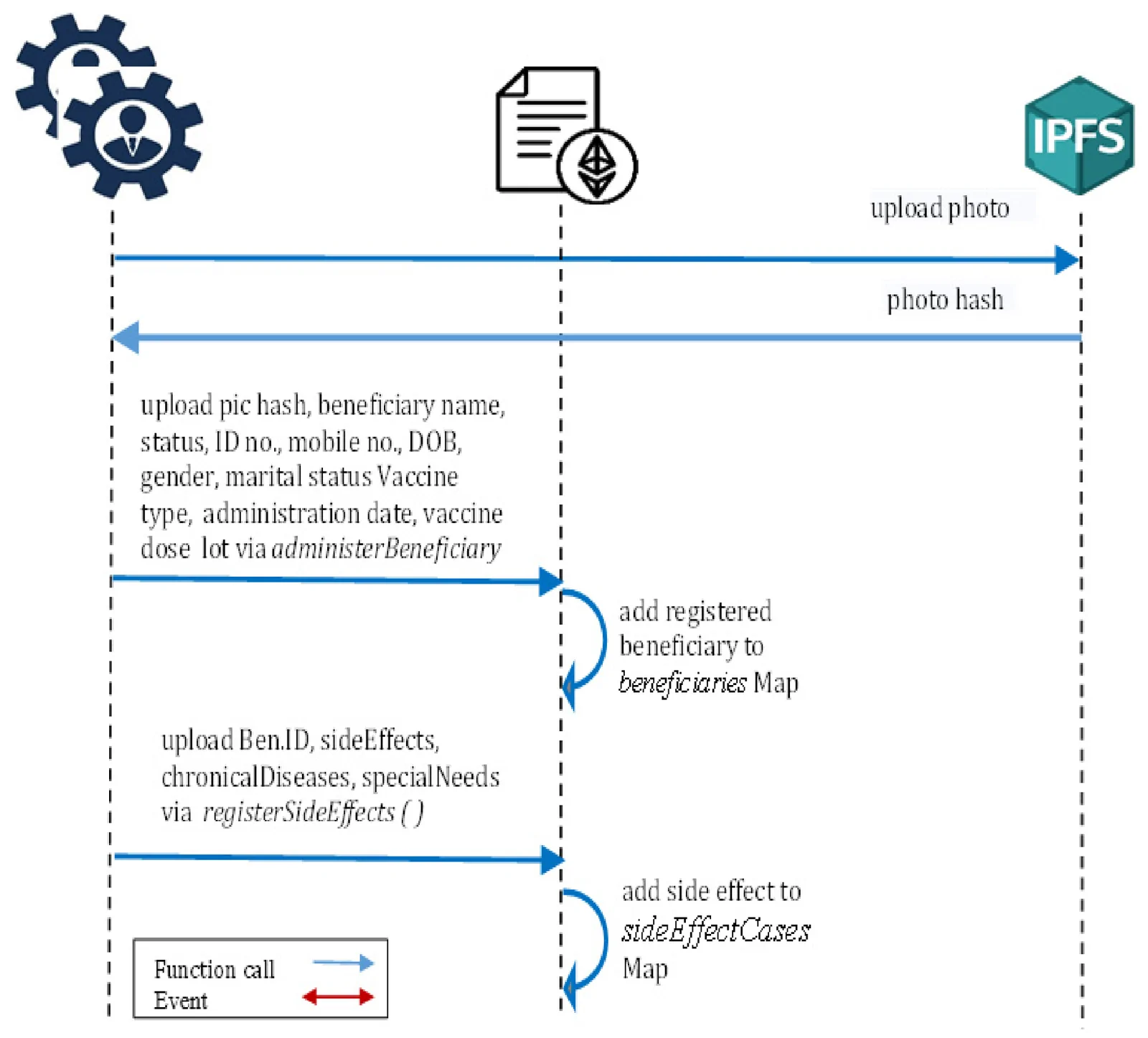
Message sequencing for beneficiaries’ metadata and side effects registration by the second smart contract.

The second smart contract was also tested. Figure 13 demonstrates the message sequencing for the beneficiaries’ metadata and side effects registration processes.

The registration and side effects smart contract was successfully deployed by the government, which was assumed to be the U.A.E. and given the EA of

“0x5B38Da6a701c568545dCfcB03FcB875f56beddC4”.

Also, the government successfully added the “Health_Admin_1” using the function *addAdmin*, where it was given the EA of

“0xCA35b7d915458EF540aDe6068dFe2F44E8fa733c”.

The administrator registered a beneficiary profile using the function *registerBeneficiaryProfile*. The following parameters were inserted:

*string*, name:Bob;

*string*, idNumber: B987654321;

*uint256*, mobileNumber: 102030;

*string*, picHash: aVmY7Frgw6rGwyUqE5uc71ThtuoAUM;

*uint256*, DOB: 1641990;

*enum*, gender: 0 (male);

*enum*, maritalStatus: 2 (single).

The smart contract verified that the ID did not already exist in the *profileExists* mapping and successfully created the beneficiary profile. The administrator then recorded Dose 1 using the function *recordVaccinationEvent* with the following parameters:

*string*, idNumber: B987654321;

*uint256*, doseNumber: 1;

*enum*, vaccineType: 1 (AstraZeneca);

*string*, vaccineDoseLot: T321.

The smart contract verified that the profile existed and that Dose 1 had not yet been recorded, then stored the vaccination event. To demonstrate multi-dose support, a second dose was recorded using *recordVaccinationEvent* with doseNumber 2 and vaccineLotNumber: T322. The smart contract accepted this call and stored Dose 2 as a separate vaccination event linked to the same beneficiary profile. This demonstrates that the system correctly supports multiple doses for the same beneficiary.

**Figure 14:**
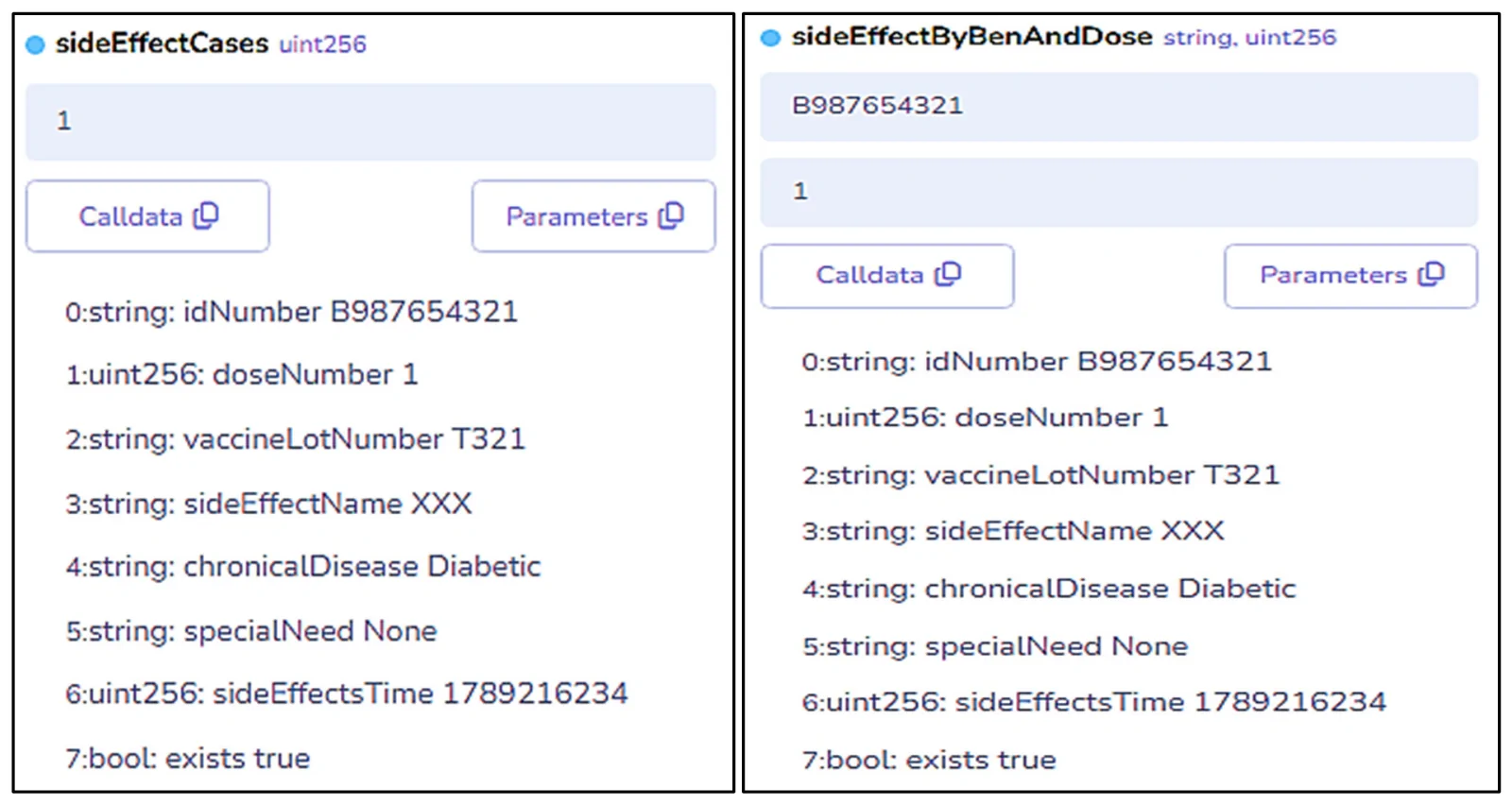
Beneficieries Map, sideEffectCases and sideEffectBen: Maps for vaccine probable side effects.

It was assumed that this beneficiary suffered from symptom “xxx”. The “Health_Admin_1” used the function *registerSideEffects* and successfully inserted the witnessed side effect along with other information, such as the patient’s ID number, the dose number, the existence of chronic disease or special needs, and the time of observing the symptom. The inserted parameters were added to *sideEffectCases* and *sideEffectByBenAndDose* Maps, as shown in Figure 14. The *vaccineLotNumber* was automatically retrieved from the *vaccinationEvents* mapping for the corresponding dose, linking the side effect record directly to the vaccine lot administered.

The *vaccinationEvents* Map provides the vaccine lot number the beneficiary received (assumed to be T321). This information was used in the cold supply chain smart contract to trace the vaccine lot’s history and check whether any violations occurred during its journey.

## 6. The Proposed Blockchain-Based Cold Supply Chain Personnel Certification and Employment System

**Figure 15:**
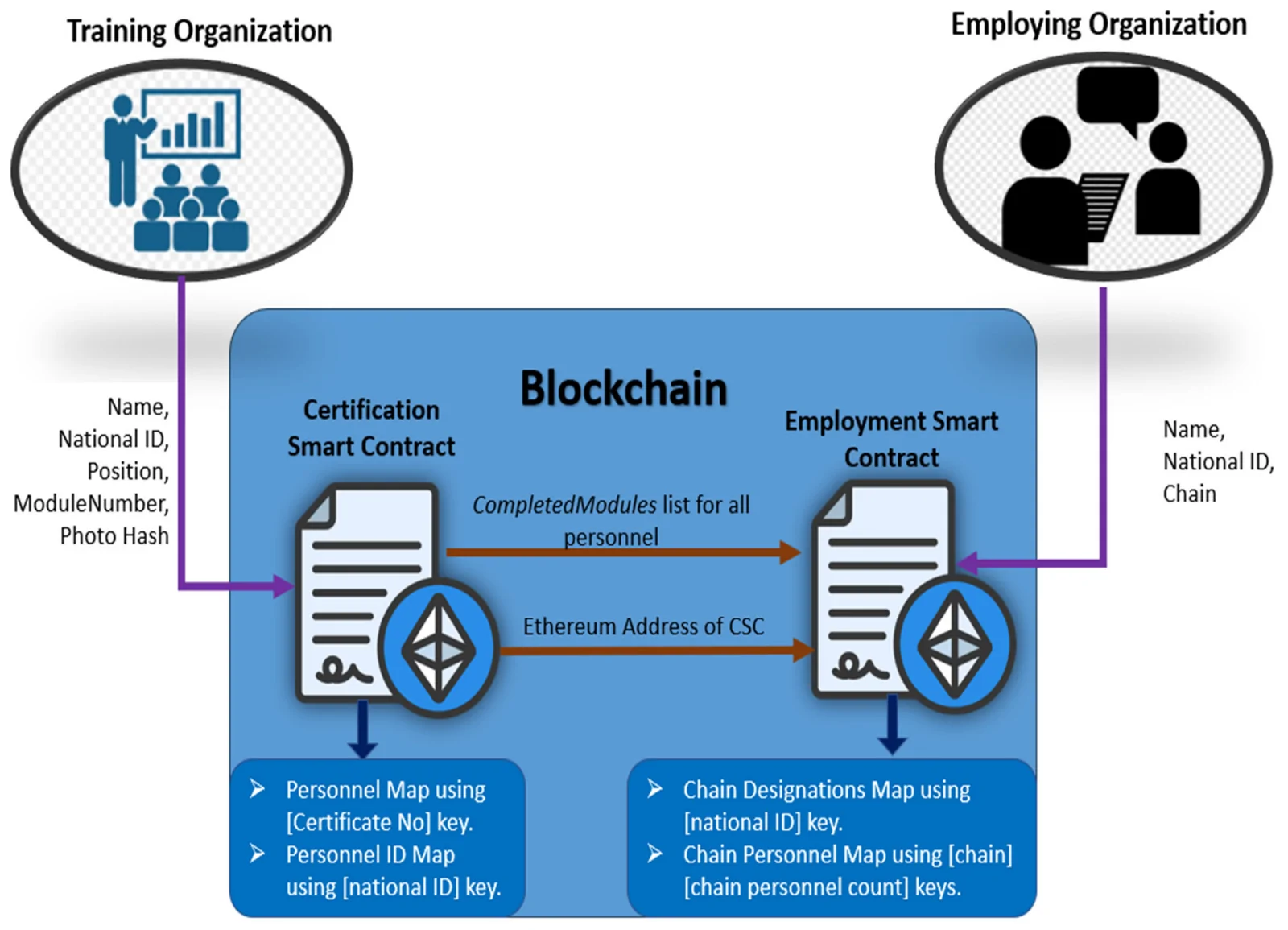
Blockchain system design architecture.

Our proposed blockchain-based system, shown in Figure 15, fills the gap in the literature and industry by proposing a framework and smart contract system for the certification and employment of cold supply chain personnel. Our system was developed on top of the Ethereum blockchain and utilized the InterPlanetary File System (IPFS), which is a decentralized storage system used for large-scale data storage [102]. The system consists of two smart contracts coded using the Ethereum blockchain language “Solidity”.

### 6.1. Certification Smart Contract

The first of the two smart contracts in the system is the certification smart contract (CSC), which includes the training modules specified and delivered by a training entity as described in Table 5. This research adopts the training delivered by the World Health Organization on handling, storing, and transporting the Pfizer-BioNTech COVID-19 mRNA vaccine, which incorporates nine modules with different content [105].

Personnel involved with the vaccine cold supply chain must complete predetermined modules according to their designations, which entitle them to perform certain roles and execute specific tasks associated with each chain within the cold supply chain as shown in Table 6. The targeted audience and their allowed training models are shown in Table 6 [105].

Smart contract administrators are responsible for certifying personnel using the developed system’s CSC at the time of completing any training module. A certificate number is then generated for each completed module by the CSCS, and the person is added to the corresponding designation list.

It is important to note that certification is issued independently for each completed module, and personnel are not required to complete all permitted modules before being certified or employed. Eligibility for assignment to a specific supply chain role is determined by whether the person holds a valid certificate in at least one of the modules required for that role designation, as detailed in Table 6.

### 6.2. Employment Smart Contract

The second contract in the system is the employment smart contract (ESC). This is deployed by the entity looking to hire qualified individuals, such as governments or healthcare organizations. As the address of the CSC is inserted into the ESC at deployment, the two smart contracts are connected. This supports authenticating personnel certification and verifying whether he/she is qualified to be assigned any job in the cold supply chain. If an attempt is made to employ an uncertified person at any chain or even employ a certified person at an inappropriate chain, the smart contract will generate an error. We refer to Section 3 to link the targeted personnel designations to the appropriate chain within the cold supply chain as shown in Table 7.

**Figure 16:**
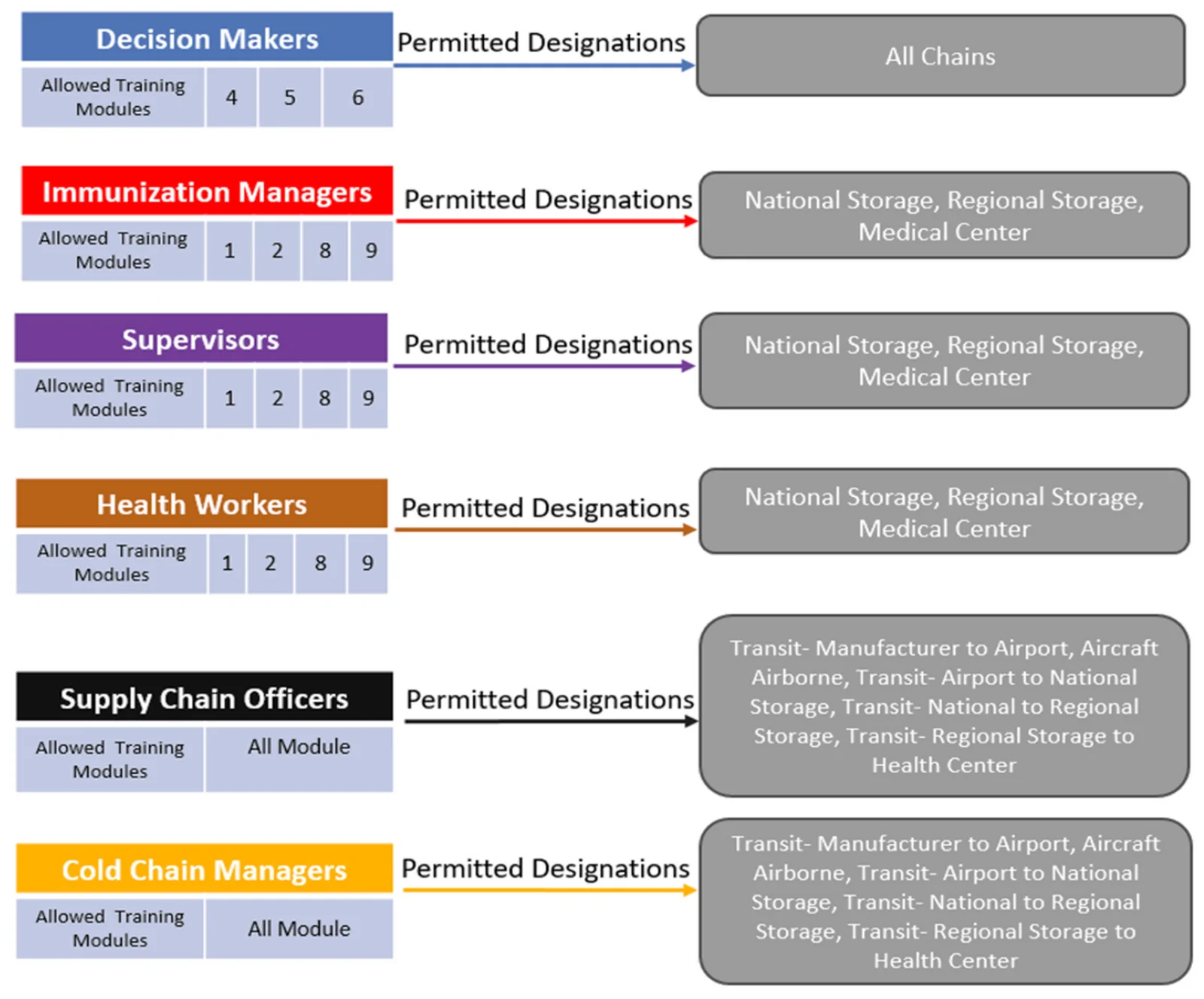
The relation between personnel, their entitled training modules and designated chains.

Figure 16 explains the relation between personnel, their entitled training modules, and designated chains. If an attempt is made to employ any personnel at a stage that is not compatible with their position as outlined above, it will be met with an error by the smart contract. Specifically, the smart contract cross-references the submitted personnel national ID against the certified personnel records in the certification smart contract, verifies that the completed module is among those required for the requested chain designation as defined in Table 6 and Table 7, and rejects the assignment transaction if the certification requirement is not met.

### 6.3. Certification and Employment System Participants and Roles

Our smart contract-based system participants and their roles are as follows:

1. Administrator(s): This is the entity that provides training and is responsible for the deployment of the certification smart contract, making the system operational and stakeholders able to interact with it using any available interface. The system administrator possesses a unique Ethereum Address (EA), which gives it privileges over the certification smart contract.
2. Trainees: Individuals seeking certification in a particular module for a specific role. They will need administrative oversight to input their data into the smart contract after successfully completing a module. Their name, national ID, position, and module number are sent to the training organization for certification using our developed system. Afterward, when they apply for a position at the hiring organization, the certificate number for their completed module is compared with the required modules for their designation.
3. Employing entity(s): The organization looking to employ qualified individuals for their supply chain. They will be responsible for the deployment of the employment smart contract, connecting their system to that of the administrator to access their database of personnel. The employing entity will also have a unique EA that will give them administrator privileges over the employment smart contract.

### 6.4. Process Flow, Algorithms, and Message Sequencing

The above smart contracts operate together to support the following processes:

- Verifiable and immutable tracking of module completion by personnel.
- Reliable and traceable employment of certified personnel at each stage of the supply chain.
- A comprehensive and cohesive system that operates in harmony to gather rosters of certified individuals. These records are stored securely and subsequently made accessible to prospective employing entities seeking to engage these qualified individuals.
- Ensuring traceability, transparency, security, reliability, authentication, and trustworthiness across the supply chain system participants and roles.

Algorithm 6 shows the logic for the certification smart contract. The algorithm includes the *certifyPersonnel* function, which will be run by administrators to certify individuals in positions corresponding to the modules they have completed. The person’s name, *nationalID*, position, *moduleNumber*, and *photoHash* will be used to generate a unique *certificateNo*. Once certified, they will be added to the mapping of qualified personnel for easy access by the employing organization.
**Algorithm 6:** Certification1:**State:**2:**Map** (uint256 *Person*) → *personnel*3:**Map** 
(*uint256 completedModules*) → *personnelID*
4:**Function:** 
*certifyPersonnel*
5:**Input:** 
*personName, nationalID, personnelPosition, moduleNumber, photoHash*
6:**If** *nationalID* ∉ in *personnel* **Map**7:**Then** add person to *personnel* **Map**8: count = 19:**Else** person count++10:**If** moduleNumber = 4 || 5 || 611: **If** *personnelPosition* = decision_Makers || supply_Chain.Officers || cold_Chain_Managers12: Generate *certificateNo* = uint256(***keccak256***(abi.encodePacked(msg.sender, nationalID,    personnelPosition, moduleNo, photoHash, regTime))) >> 19213:**Mapping** (*personName*, *nationalID*, *personnelPosition*, *moduleNo*, photoHash → *personnelID*
**Map**14:**Else revert** error15:**Else if** moduleNumber = 1 || 2 || 8 || 916:**If** *personnelPosition* = immunization_Managers ||supervisors ||health_Workers ||supply_Chain_Officers ||cold_Chain_Managers17:  Generate *certificateNo* = uint256(***keccak256***(abi.encodePacked(msg.sender, nationalID,    personnelPosition, moduleNo, photoHash, regTime))) >> 19218:**Mapping** (personName, nationalID, personnelPosition, moduleNo, photoHash → *personnelID*
**Map**19:**Else revert** error20:**Else if** *moduleNumber* = 3 || 721:**If** *personnelPosition* = supply.Chain_Officers || cold.Chain_Managers22:  Generate *certificateNo* = uint256(***keccak256***(abi.encodePacked(msg.sender, nationalID,    personnelPosition, moduleNo, photoHash, regTime))) >> 19223:**Mapping** (personName, nationalID, personnelPosition, moduleNo, photoHash → *personnelID*
**Map**24:**Else revert** error25:**Else revert** error

Algorithm 7 demonstrates the logic for the employment smart contract. This algorithm includes the *assignPersonnel* function, which will be run by administrators to assign individuals to their desired chain, provided they are qualified. This is performed only after providing the smart contract with the address of the certification smart contract so that its certificate records can be accessed. Successfully employed individuals will be added to the *chainsDesignations* mapping along with their name, *nationalID*, and chain.
**Algorithm 7:** Assigning Personnel1:**State:**2:**Map** (uint256 *PersonChain*) → *chainDesignations*3:**Map** ((uint256 *countChain* (uint256 *PersonChain*)) → *chainPersonnel*4:**Function**: *assignPersonnel*5:**Input**: *personName*, _*nationalID*, _*chain*, *addressOfCertifyContract*6:Capture the address of *PersonnelCertificate* contract7:**If** _*chain* = Manufacturer8:**If** _*nationalID* ∈ *personnel*
**Map**9:**Then** ∀ (_*nationalID* position) **If** _*nationalID* position = decision_Makers10:**Then** add _*nationalID* to *chainDesignations* **Map**11:*hasRequiredModules***= true**12:**Else revert** error13:**Else if** _*chain* = National_Stg || Regional_Stg || Medical_Center 14:**If** _*nationalID* ∈ *personnel*
**Map**
15:**Then** ∀ (_*nationalID* position) **If** _*nationalID* position=decision_Makers||immunization_Managers||supervisors ||health_Workers16:**Then** add _*nationalID* to *chainDesignations* **Map**17:*hasRequiredModules*** = true**18:**Else revert** error19:**Else if** _*chain* = Transit_Mfr_Apt||Aircraft_Airborne||Transit_Apt_National||Transit_National_Regional||Transit_Regional_Med20:**If** _*nationalID* ∈ *personnel*
**Map**
21:**Then** ∀ (_*nationalID* position) **If** _*nationalID* position = decision_Makers || supply_Chain_Officers || cold_Chain_Managers22:**Then** add _*nationalID* to *chainDesignations* **Map**23:*hasRequiredModules*** = true**24:**Else revert** error25:**Else revert** error

### 6.5. Message Sequencing

As per the system architecture, the process begins with the training entity inserting the personnel details into the CSC. An administrator in the system calls the *certifyPersonnel* function, entering the person’s name, *nationalID*, position, *moduleNo*, and *photoHash*. The CSC will automatically check the coded rules and conditions, which include matching the compatibility of the person’s position and the *moduleNo*. If all conditions are fulfilled, the CSC generates a unique, tamper-resistant and secure cryptographic number indicating the certification of that person. The *CertificateNo* is created by the smart contract using the *keccak256* cryptographic function applied to the administrator’s Ethereum address, the person’s *nationalID, personnelPosition, moduleNo, photoHash*, and the timestamp of registration, with a 192-bit right shift applied to produce a compact and unique identifier. Also, the CSC maps the person’s details into the *personnelID* map using the certified person’s *nationalID* as the input key. In addition, the CSC maps the person’s details into the *Personnel* map using their unique *CertificateNo* as the input key. Having two separate maps allows employing entities to later perform the following two actions:

- Input a person’s *nationalID* to access all their certificates and all modules they have completed.
- Input *CertificateNo* to check the validity of the certificate, who it belongs to, and what module it certifies.

**Figure 17:**
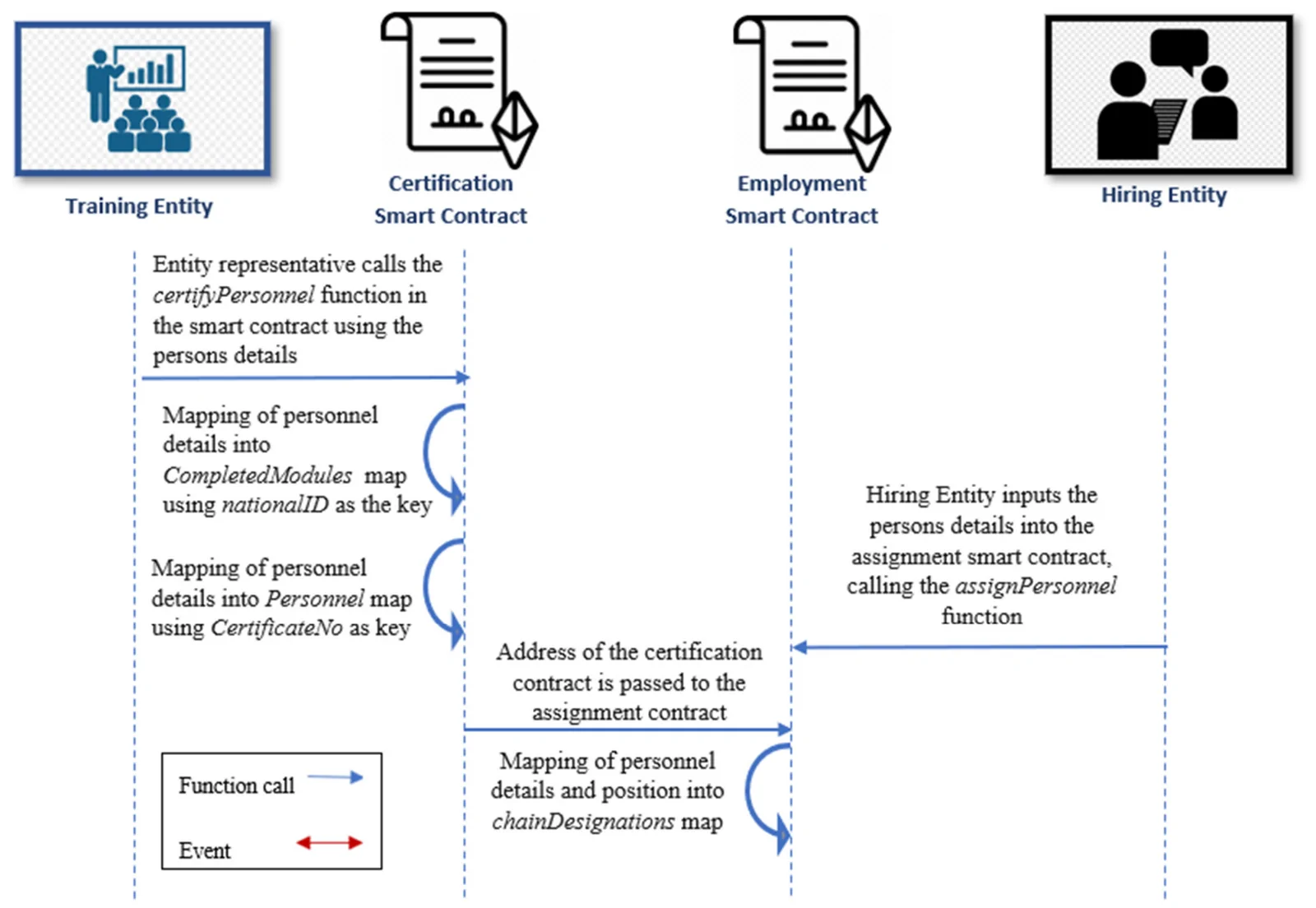
The Certify-Assign system message sequencing.

The next step occurs when an employment entity is looking to hire qualified individuals for their supply chain. It is important to note that, while there is generally only one CSC to record all certified individuals, there will be several ESCs interacting with it, as each employment entity must deploy its own ESC for its supply chain. Figure 17 shows the system message sequencing for one such entity.

The hiring entity first deploys its ESC, which will interact with the original certification smart contract. The address of the CSC is inserted into the ESC, connecting the two. Administrators are given access to add personnel to the system. When hiring begins for a new position, an administrator calls the *assignPersonnel* function in the ESC, inputting the person’s name, *nationalID*, and desired chain designation. The *assignPersonnel* function automatically checks the *CompletedModules* array list in the CSC and searches for the person’s details. If the person is found to be certified in one of the modules needed for the desired chain designation, they are accepted, and subsequently added to *chainDesignations* mapping with their name, *nationalID*, completed module number, and chain designation. Otherwise, if the person is not found to be certified in any of the required modules, they are not added to the mapping. Instead, the ESC displays an error stating the person is not qualified for the desired chain. This process is repeated for each individual before they are hired and added to the chain.

In this manner, we can ensure that all personnel employed within the supply chain receive comprehensive training in the proper handling of the vaccine. Furthermore, this information, including certification records, will be securely stored on the blockchain. This meticulous record-keeping approach not only facilitates the reinforcement of accountability but also enables precise tracking of errors and their attributions.

## 7. The Blockchain Certification and Employment System Deployment and Testing

Our smart contracts were developed and tested using Remix IDE, which is a powerful integrated development environment designed specifically for testing and deploying smart contracts on various blockchain platforms, such as Ethereum. Remix IDE offers a web-based interface, making it easily accessible from any modern web browser.

The first of the two smart contracts in the system, the certification smart contract, was successfully deployed by the training entity, which is assumed to be Pfizer and was given the EA: 0x5B38Da6a701c568545dCfcB03FcB875f56beddC4.

To test the *addAdmin* function, a new administrator called “Admin1” was added to the system with the EA: 0xd9145CCE52D386f254917e481eB44e9943F39138”. The new administrators could be another training entity or any stakeholder of interest to the system. For testing purposes, a user designated as a decision maker (*personnelPosition* Enum 1) was assumed to have completed training Module 4, thus, the administrator used the *certifyPersonnel* function to certify him by inserting the following parameters:

*string*, personName: Trainee1;

*uint256*, nationalID: 123;

*enum*, personnelPosition: 1 (*decision_Maker*);

*enum*, moduleNo: 4;

*string*, photoHash: rni2n45in5b.

**Figure 18:**
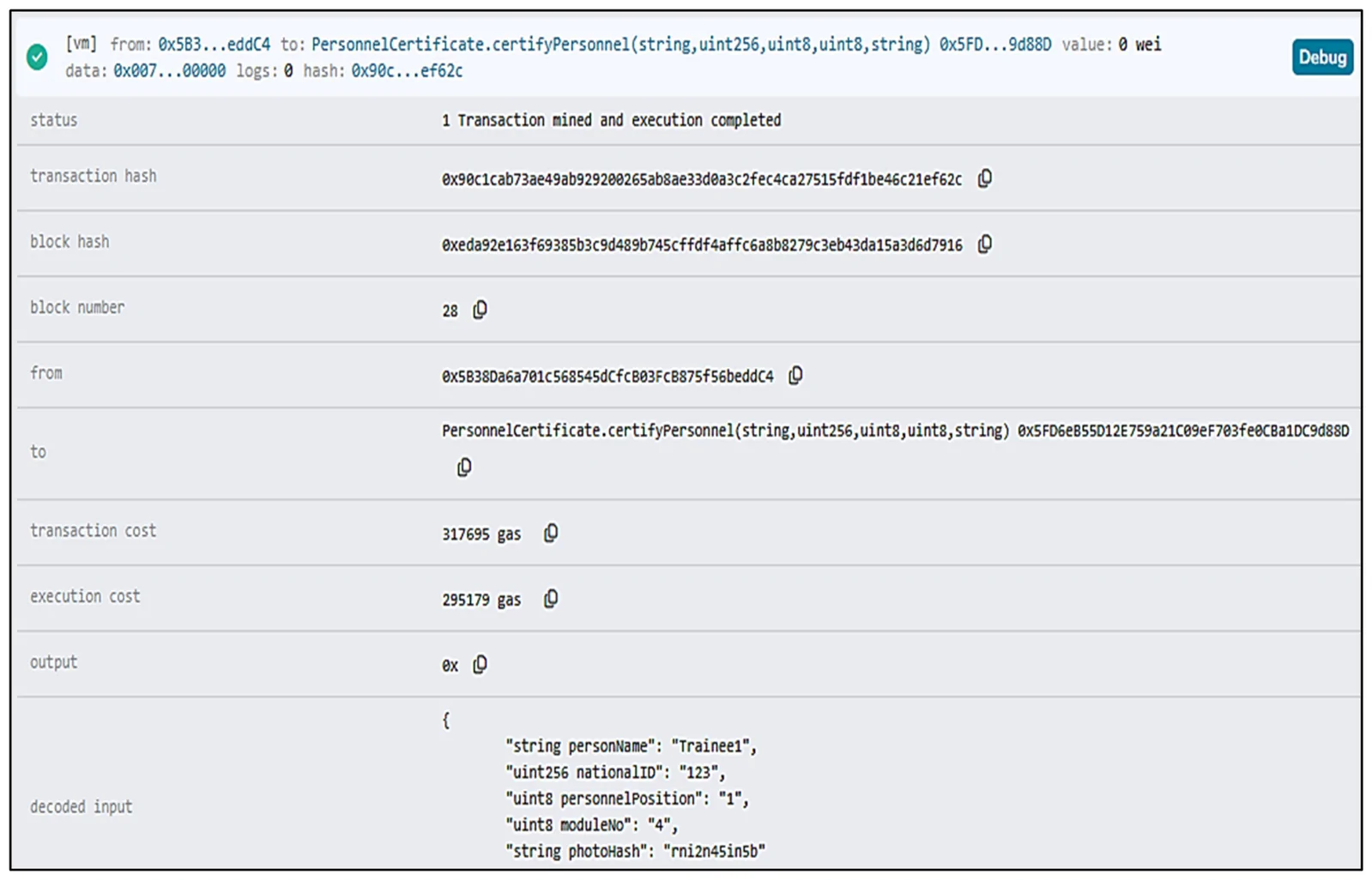
Successful completion of Module 4 by a decision maker.

**Figure 19:**
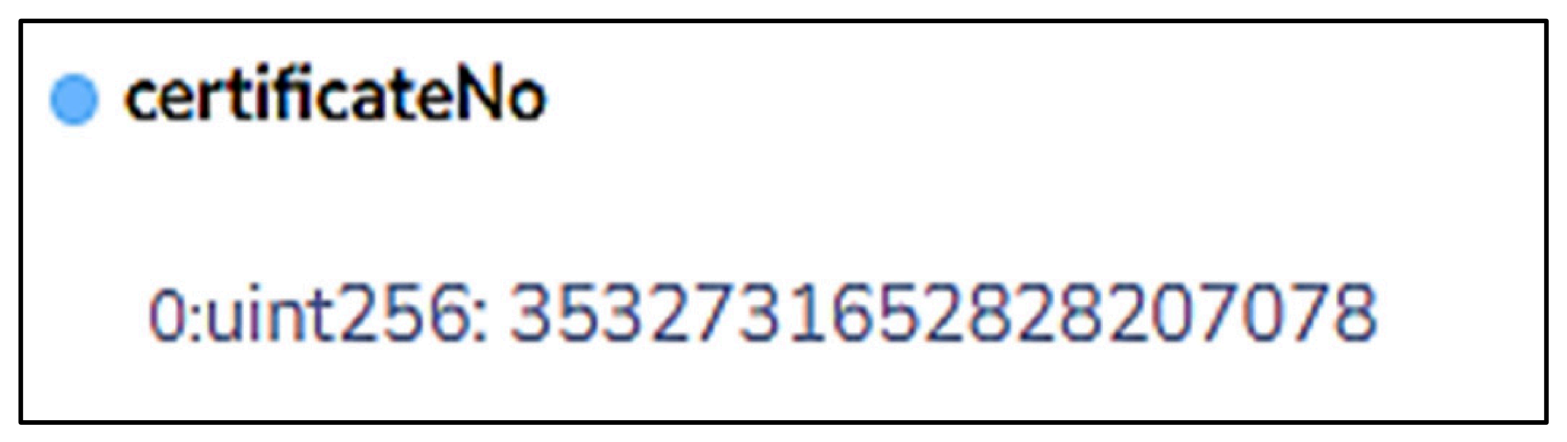
Generated *CertificateNo*.

The smart contract executed the function, as shown in Figure 18, since Module 4 is among the compatible modules for decision makers. This result is consistent with the intended qualification rule—certification is issued per completed module, and a decision maker who has completed Module 4 receives a valid certificate for that module. The system does not require simultaneous completion of all permitted modules (4, 5, and 6) before any certification is issued. Additional certificates are generated independently as further modules are completed, enabling progressive accumulation of qualifications over time. The contract generated a unique certificate number as shown in Figure 19.

For further testing, it was assumed that there was an attempt to certify the same user for Module 2 using the *certifyPersonnel* function with the following input parameters:

*string*, personName: Trainee1;

*uint256*, nationalID: 123;

*enum*, personnelPosition: 1 (decision_Maker);

*enum*, moduleNo: 2;

*string*, photoHash: rni2n45in5b.

**Figure 20:**
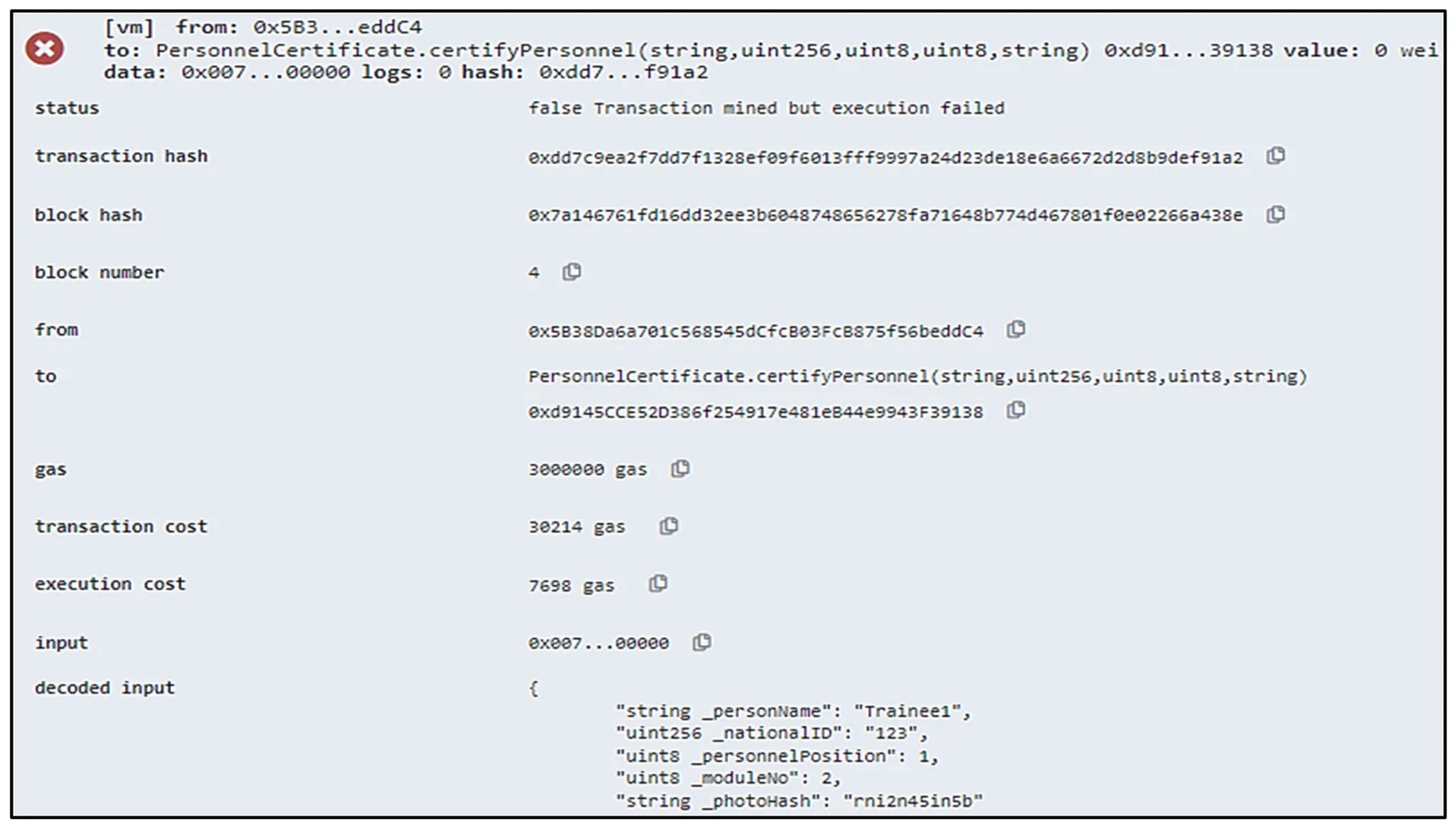
Error when attempting to certify a decision maker for an incompatible module.

As shown in Figure 20, the smart contract did not execute the function and reverted to an error, as decision makers are only eligible to take training Modules 4, 5, and 6.

**Figure 21:**
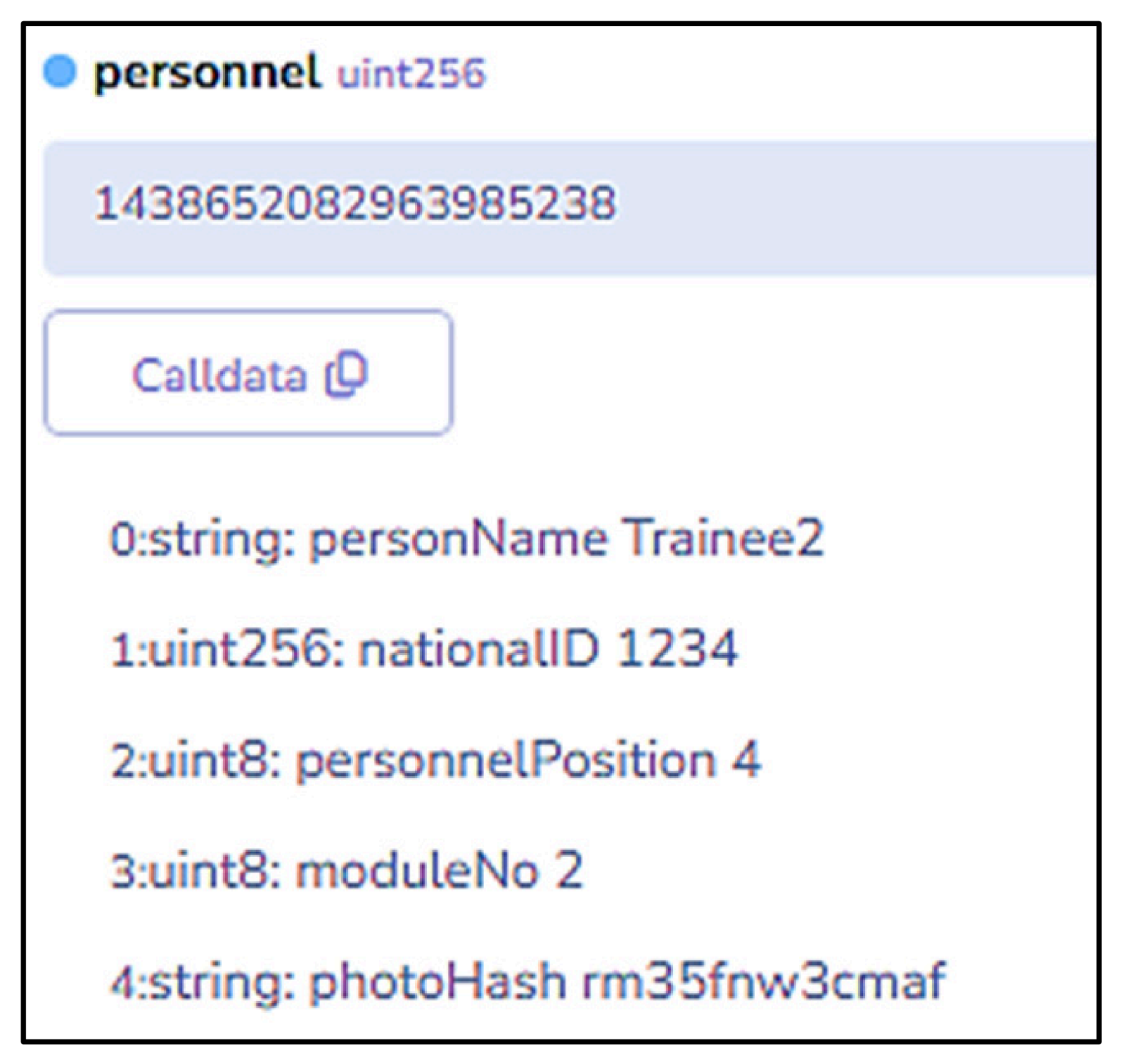
*Personnel* and *chainDesignations* map using *CertificateNo* and *nationalIDs* keys.

Also, a new user, designated as a health worker (*personnelPosition* Enum 4), was assumed to have completed training Module 2. The smart contract generated a certificateNo for this health worker once the administrator called the *certifyPersonnel* function, as health workers are eligible to take Modules 1, 2, 8, and 9. The contract generates a *CertificateNo* for each module completed by a user, using the keccak256 function to create a unique ID using the *EA*, *nationalID*, *personnelPosition*, *moduleNo*, *photoHash*, and *regTime* of the person. As seen in Figure 21, this *CertificateNo* can be input into the *Personnel* function to reveal the details of the certification.

The second smart contract in the system is the employment smart contract (ESC), which is used to hire qualified personnel to their respective chains within the cold supply chain. The contract was successfully deployed with a selected EA of “0xDA0bab807633f07f013f94DD0E6A4F96F8742B53”. This smart contract is deployed by the organization, government, health authority, supply chain, or logistics company intending to hire qualified individuals. After deployment, the address of the *PersonnelCertificate* smart contract was passed to the ESC to grant it access and enable checking for certified personnel. To test this contract, it was attempted to employ Trainee1, who is certified as a decision maker, to the manufacturer chain (chain 1) by inserting the following input parameters to the *assignPersonnel* function:

*string*, personName: Trainee1;

*uint256*, nationalID: 123;

*enum*, chain: 1 (*manufacturer*).

**Figure 22:**
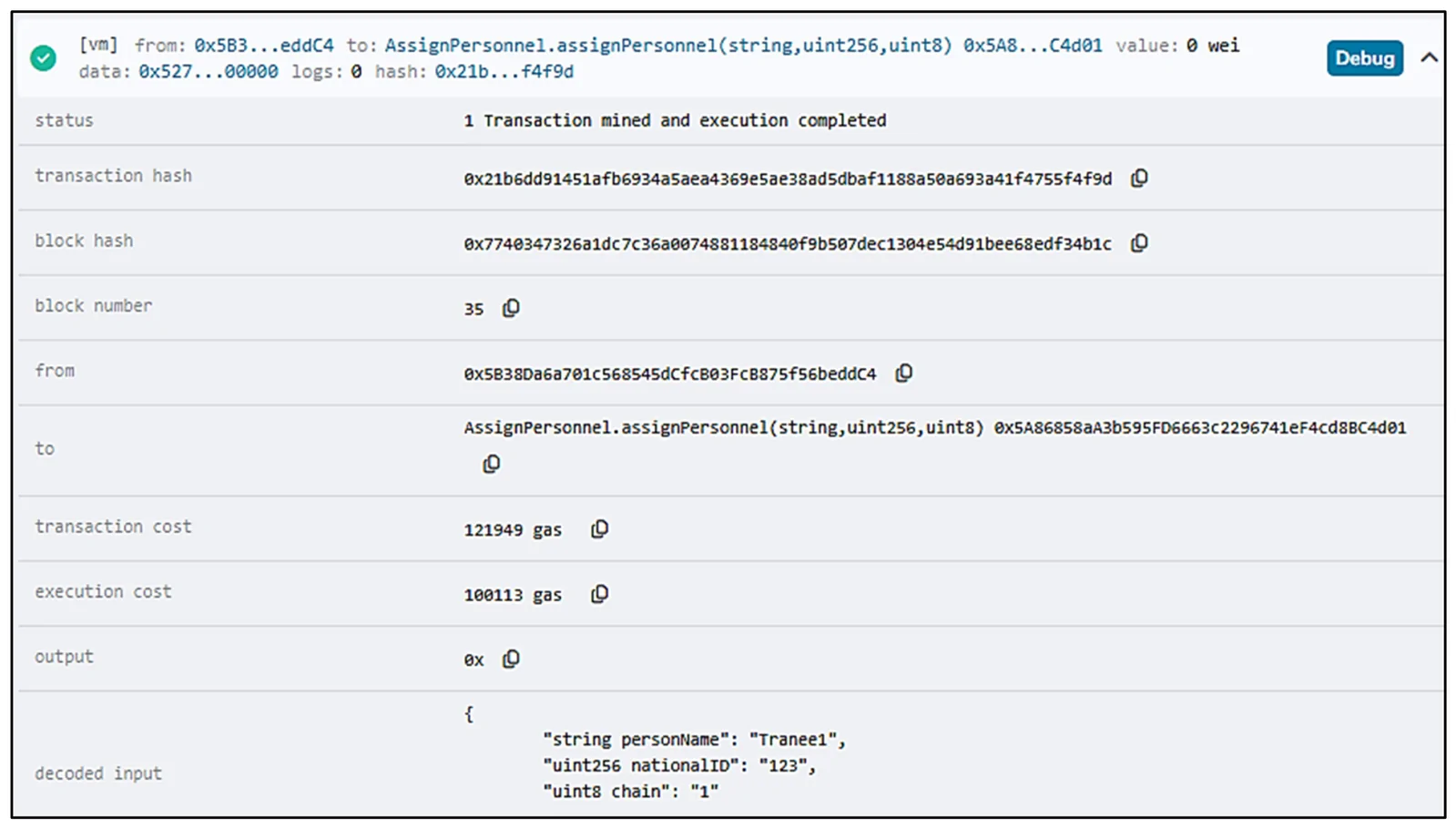
Successfully employing a decision maker in the manufacturer chain.

Since decision makers are needed in all stages of the supply chain, they can be employed in all chains. The employment smart contract executed the function as shown in Figure 22, and Trainee1 was successfully assigned to the *manufacturer* chain by adding them to the *chainDesignations* map with chain number 1 (*manufacturer*).

For testing purposes, it was assumed that there was an attempt to assign Trainee2, who is only certified as a health worker, to the *manufacturer* chain using the *certifyPersonnel* function with the following input parameters:

*string*, personName: Trainee2;

*uint256*, nationalID: 1234;

*enum*, chain: 1 (*manufacturer*).

**Figure 23:**
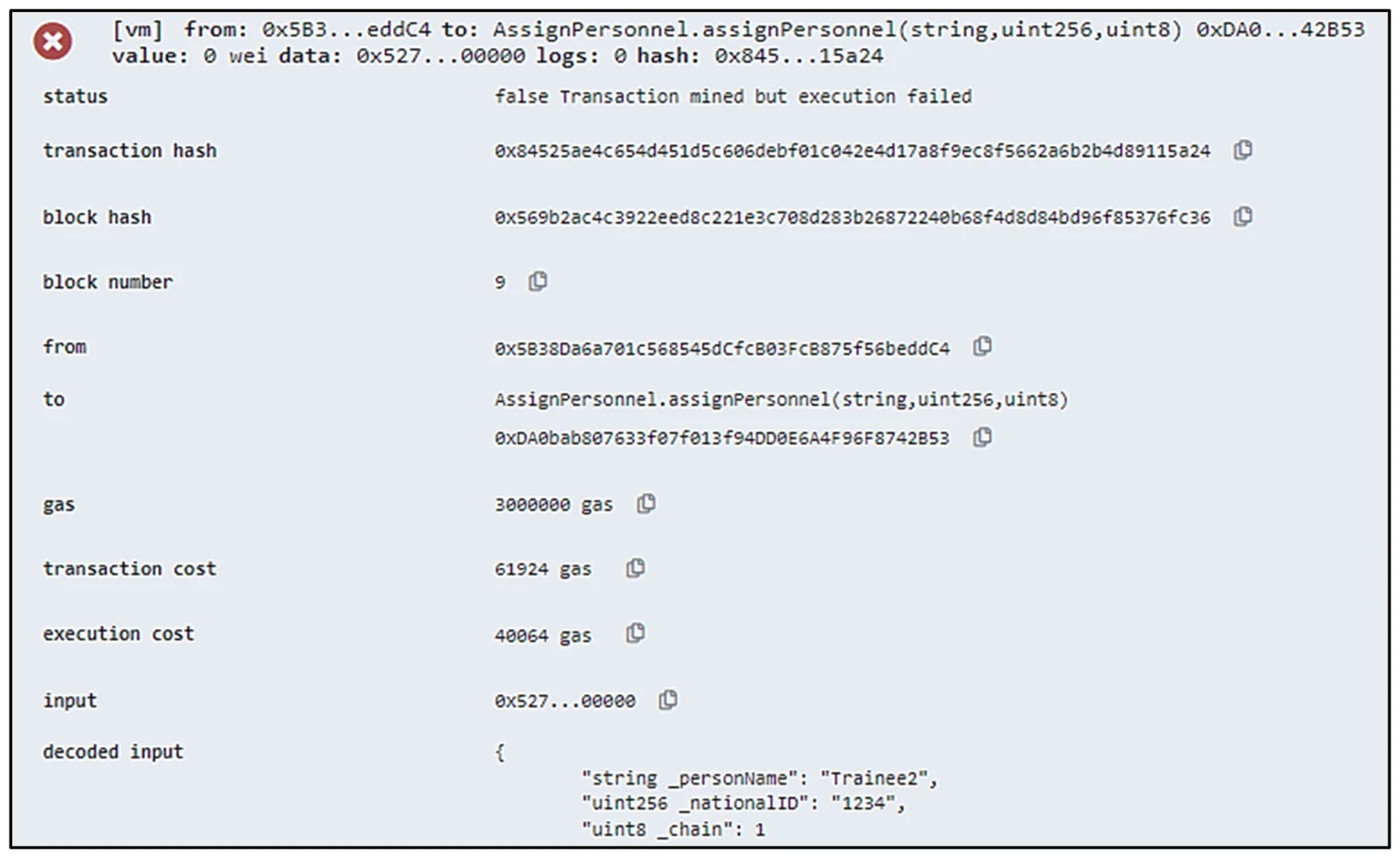
Error when attempting to assign a health worker to an incompatible chain.

As shown in Figure 23, the smart contract did not execute the assignment function and returned an error, as health workers can only be employed at *National_Storage, Regional_Storage*, and *Medical_Center* chains within the cold supply chain.

This smart contract provides two useful maps: the *chainDesignations* map, which takes the person’s national ID as input and demonstrates his/her name and the chain of employment; and the *chainPersonnel* map, which takes the chain number and chain employers count as inputs and demonstrates information about each employed personnel in that particular chain. The *chainPersonnel* map is vital in the event of a violation detected by the cold supply chain smart contract, especially when associated with side effects detected by the side effects smart contract, enabling investigators to identify the personnel assigned to the affected stage and supporting the accountability investigation process. The data connectivity provided by the platform represents a key contribution of the proposed system, enabling traceability across beneficiary, cold chain, and personnel records within a single integrated platform.

Beyond this, further scenarios were tested to ensure the desired outcome in all cases. To verify the efficacy and integrity of the system, rigorous testing protocols were implemented. Different personnel were thoroughly certified in all nine modules and associated positions, ensuring a diverse pool of qualified individuals. Subsequently, the system underwent rigorous examination to validate its capability of accurately assigning personnel to roles within the appropriate chains. This examination aimed to guarantee that only individuals with compatible skill sets and expertise were assigned to their designated roles, thereby maximizing the overall efficiency and performance of the system.

## 8. The Developed Blockchain Platform Analysis

The effective and reliable operation of our designed smart contracts is demonstrated by the correct and successful implementation of all features of the testing scenarios. However, an in-depth assessment requires consideration of the system’s cost and security aspects.

### 8.1. Economic Feasibility and Cost Analysis of the Blockchain-Based System

When considering smart contract costs, it is well known that certain “Gas” charges are necessary to deploy and perform functions [106]. The Remix IDE console shows the gas fees of each function, and an ETH gas fees calculator is used to calculate it [107]. In contrast to fiat money, the price of gas fluctuates. As such, our system’s transaction costs were calculated at the price of Ether = $2541.60, which was recorded at the time of conducting this research. Table 8 demonstrates the transaction cost of the cold supply chain smart contract and the vaccine side effects smart contract functions in Ether and in (USD).

According to the above table, the government incurs the highest expense when the vaccine system is deployed. This is because using a constructor during deployment necessitates writing and saving numerous variables for the first time on-chain. The average deployment transaction cost paid to execute this transaction is roughly $9.56 for the cold supply chain smart contract and $10.98 for the registration and side effects smart contract, based on the price of ether. This results in a total system deployment cost of almost $20.54 each time a vaccine shipment is ordered, which is regarded as a fairly affordable and acceptable price.

When examining the other system functions, it is noticed that the *registerSideEffects, tempData, lightExposure*, and *registerBeneficiaryProfile* functions are the most expensive to perform, with transaction costs between $0.967 and $2.19. This is to be expected as these are the most complex functions and have the largest on-chain storage requirements. These functions also demand that a variety of requirements be met and that mapping variables be adjusted. For the remaining functions, their transaction cost is less than $0.8.

As per the Certification and Employing smart contracts, Table 9 shows the cost of executing their functions.

Clearly, the hiring organization looking for qualified individuals bears the highest expense of deploying the employment smart contract for their organization. This mainly comes from the cost of the constructor, which, on average, is $8.05. The cost of the *setAddress* is a negligible $0.238. The administrators in this contract will bear the expense of the *assignPersonnel* function, which is around $0.631. The certifying entity bears the expense of $5.52 for deploying the certification smart contract. In addition, the certifying entity is responsible for adding administrators to the smart contract. This costs around $0.5. The administrators in this smart contract will bear the cost of running the *certifyPersonnel* function, which is $1.63 per transaction. The demonstrated fees are relatively reasonable costs that need to be covered every time a significant procedure, such as registering beneficiaries and potential side effects, adding administrators, expanding supply chains, updating the location of vaccination shipments, recording temperature and light violations, certifying and employing an individual, is carried out.

The transaction costs presented in Table 8 and Table 9 reflect individual operation costs at the Ethereum price recorded at the time of testing. Ethereum gas prices are subject to variability, fluctuating with network congestion, ETH market conditions, and protocol developments [106]. The costs reported therefore represent an indicative snapshot at a specific point in time, and the following workload-based analysis uses these costs consistently to provide a transparent and comparable economic assessment.

To assess economic feasibility at a realistic operational scale, Table 10 presents a synthetic workload-based cost scenario. Rather than mapping to a single historical campaign, this model combines verified operational parameters from distinct regional datasets to construct a robust testing benchmark. The baseline population and delivery volume are scaled based on the documented UAE COVID-19 national vaccination campaign. The UAE Ministry of Health and Prevention (MoHAP) reported that a total of 16,444,844 [108] doses were administered across approximately 7,684,876 first-dose recipients [109]. To complement this scale with realistic IoT environmental stress metrics, the system’s edge case recording constraints are evaluated using empirical cold chain failure rates from documented field evaluations in India, which established temperature violations ranging from 1.3% to 13% across varying logistical tiers [110].

The resulting composite workload scenario in Table 10 covers 1,000,000 beneficiaries receiving two doses each, 100 vaccine batches each traversing nine supply chain stages, and 500 certified cold chain personnel, representative of the healthcare workforce mobilized at a regional level within a campaign of this scale. Table 10 shows the workload-based economic feasibility estimates based on a composite global vaccination campaign scale and transaction costs from Table 8 and Table 9.

The total estimated campaign cost of approximately $2.46 million (when accounting for a worst-case 13% temperature violation rate for 1,000,000 beneficiaries [110]) corresponds to approximately $2.46 per beneficiary for the complete blockchain-recorded lifecycle.

The side effect recording component is calculated at the WHO-reported AEFI rate of 130.1 per 100,000 doses [111], which represents only reported events. Published evidence demonstrates that passive reporting systems capture only a fraction of actual adverse events, a cross-sectional study of 2993 COVID-19 vaccine recipients found spontaneous reporting rates of only 11.6% after the first dose and 12.7% after the second dose [112]. Even if the system captures ten times the currently reported rate, the additional cost amounts to approximately $56,984 for 2,000,000 doses, increasing the per-beneficiary cost from $2.46 to approximately $2.52, which is economically negligible relative to the clinical value of the additional surveillance data captured.

The economic justification for the proposed system extends beyond transaction cost to the consequence of failing to detect and trace cold chain failures and adverse events. According to the WHO, nearly 50% of vaccines are wasted annually due to cold chain failures [113], with the global annual cost of pharmaceutical cold chain failures estimated at $35 billion [114]. In England, the National Audit Office reported that approximately 4.6 million COVID-19 vaccine doses were wasted by October 2021 [115]. In the United States, 82.1 million COVID-19 vaccine doses were discarded between December 2020 and May 2022, representing approximately $1.1 billion in losses at prevailing vaccine prices [116]. A single cold chain failure can cost between tens of thousands and several million dollars when product destruction, regulatory penalties, recall logistics, and reputational damage are included [117,118]. The prevention of even one major cold chain failure through early detection and corrective action would recover the full estimated system cost of $2.46 million many times over.

The costs in Table 10 reflect deployment on the public Ethereum blockchain as used in this system. For national government implementations, permissioned blockchain architectures, such as Ethereum Besu or Hyperledger Fabric, could be considered, operating within a government-authorized consortium of supply chain stakeholders and eliminating gas costs entirely while preserving immutability and auditability. For public Ethereum deployments, Layer-2 rollup solutions could be considered, offering transaction cost reductions of 10 to 50 times relative to the base layer [119] while inheriting its security guarantees. The per-transaction costs in Table 8 and Table 9, therefore, represent a conservative upper bound on operational costs under the current deployment architecture.

### 8.2. Security and Privacy Analysis of the Blockchain-Based System

The developed system presents the following important features inherited from its backbone technology of blockchain:

- Man In The Middle (MITM) Attack: In a blockchain network, this attack is substantially mitigated since all transactions must be signed by the sender’s private key; therefore, any attempt to alter the transaction’s content would require the intrusive party to sign the transaction using the sender’s private key, which is practically infeasible and cryptographically intractable. Digital signatures provide transaction authenticity and integrity, ensuring that transactions originate from the registered sender and have not been altered in transit, which significantly contributes to ensuring that information is supplied by authorized system participants, an element that is crucial for the vaccination system’s effectiveness. In addition to transaction-level security, the proposed system enforces data source integrity by restricting sensor data submission exclusively to the registered oracle address, as described in Section 4.2.
- Availability and Accessibility: The Ethereum blockchain has a distributed, decentralized structure. As a result, all of the participating nodes will keep records of smart contract logs and transactions. This characteristic enables the blockchain network to continue operating and being active, even in the event of a node failure. In a dispersed network, the only scenario in which the network would go down is if all nodes failed, which is exceedingly rare.
  Accountability and Authorization: The developed system uses Ethereum smart contract Modifiers to assign the various participants to specific roles and responsibilities. Consequently, all system participants are held responsible for their actions, especially since transactions are recorded on an immutable ledger.
  The system additionally addresses the following security considerations:
- Reentrancy and Access Control: Reentrancy vulnerabilities are prevented through the Checks–Effects–Interactions pattern applied throughout the smart contract functions. Access control violations are prevented through role-specific modifiers, *onlyAdmin* restricting administrative functions to registered administrators, and *onlyGov* restricting supply chain governance functions to the government deployer, which restrict all write operations exclusively to authorized Ethereum addresses.
- Integer Arithmetic: Integer overflow and underflow vulnerabilities are mitigated by the built-in arithmetic protection of Solidity version 0.8.x, which automatically reverts transactions where arithmetic boundaries are exceeded.
- Replay Attacks: Replay attacks are prevented at the Ethereum protocol level through transaction nonces, which ensure each transaction can only be executed once.
- Timestamp Dependency: The timestamp dependency finding identified by Slither in the *amendSideEffect* function is a false positive. The timestamp in that function is used exclusively to record the time of the amendment event and is not used in any conditional logic that could be manipulated.
- Malicious and Compromised Administrators: The risk of malicious insiders and compromised administrators is addressed through the *revokeAdmin* function included in the revised smart contract, which immediately removes write access from any administrator address. All past transactions by revoked administrators remain on-chain as an immutable accountability record.
- Private Key Compromise: The risk of private key compromise is an operational security consideration addressed through organizational key management protocols at the deployment level, including secure key storage practices and access controls implemented by the deploying health authority.
- Wallet Integrity and Front-end Application Security: Administrators interact with the smart contracts through wallet software and front-end interfaces. The security of these components, including wallet integrity and front-end application security, is maintained through standard secure software development and deployment practices at the operational level, including the use of trusted wallet applications, encrypted communications, and validated front-end interfaces.
- Contract Immutability: The proposed system deploys smart contracts as immutable code on the Ethereum blockchain. Once deployed, the contract logic cannot be altered or replaced, ensuring that no party can substitute the original contract with a modified or malicious version after deployment. This immutability is a deliberate security property of the system design.

**Figure 24:**
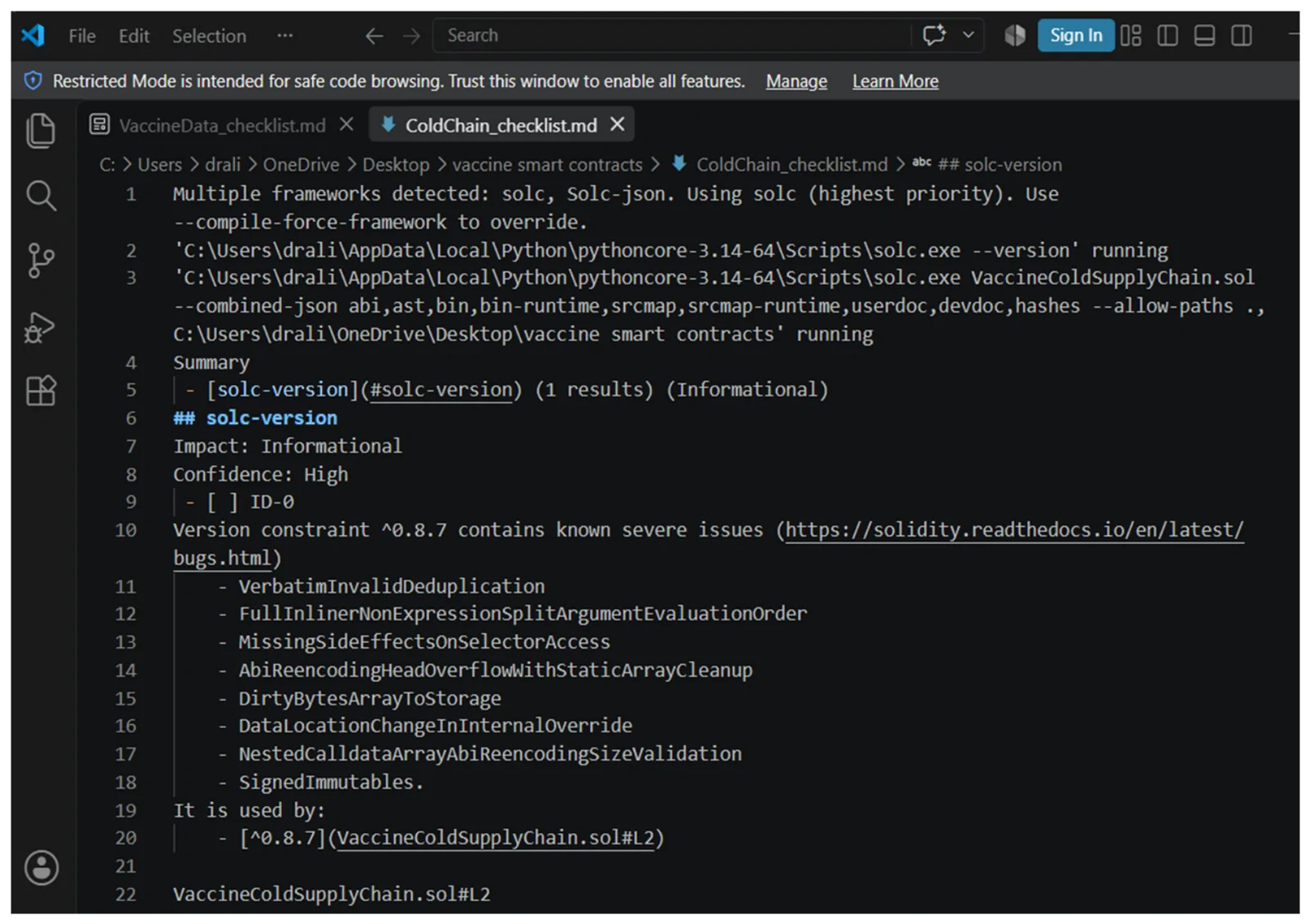
Slither report for the VaccineColdSupplyChain smart contract.

**Figure 25:**
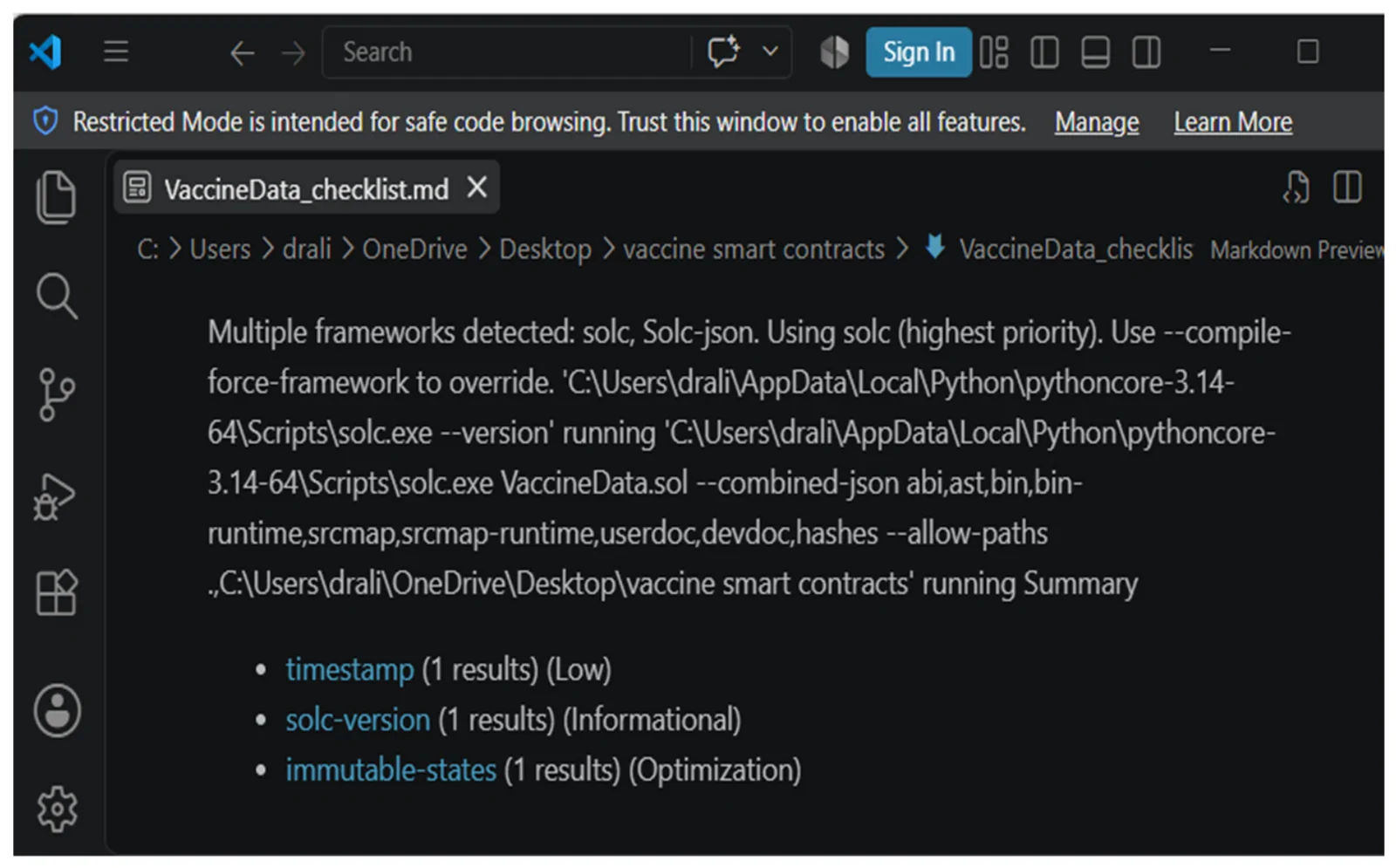
The Slither report for the VaccineData smart contract.

**Figure 26:**
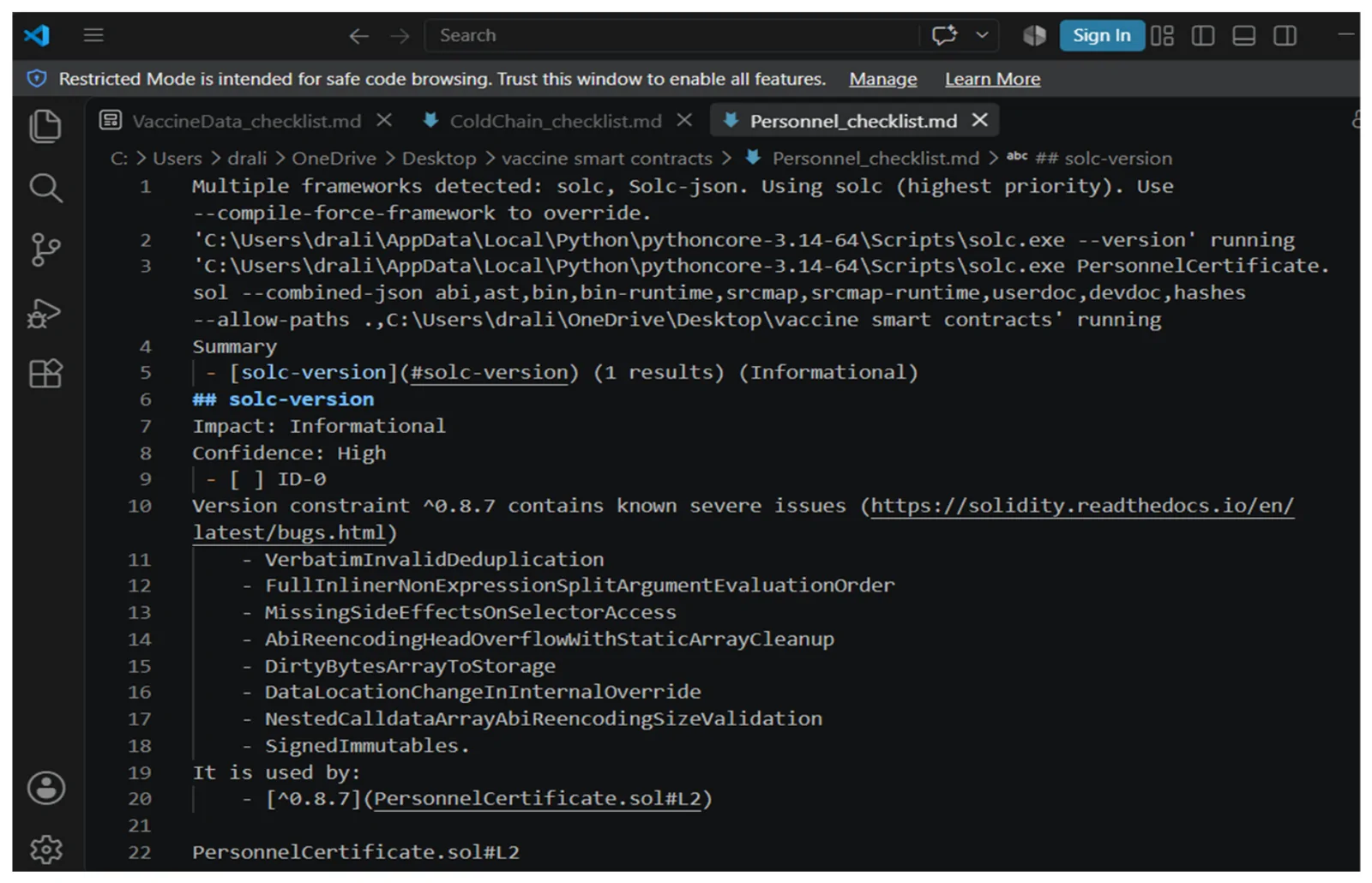
Slither report for the PersonnelCertificate smart contract.

**Figure 27:**
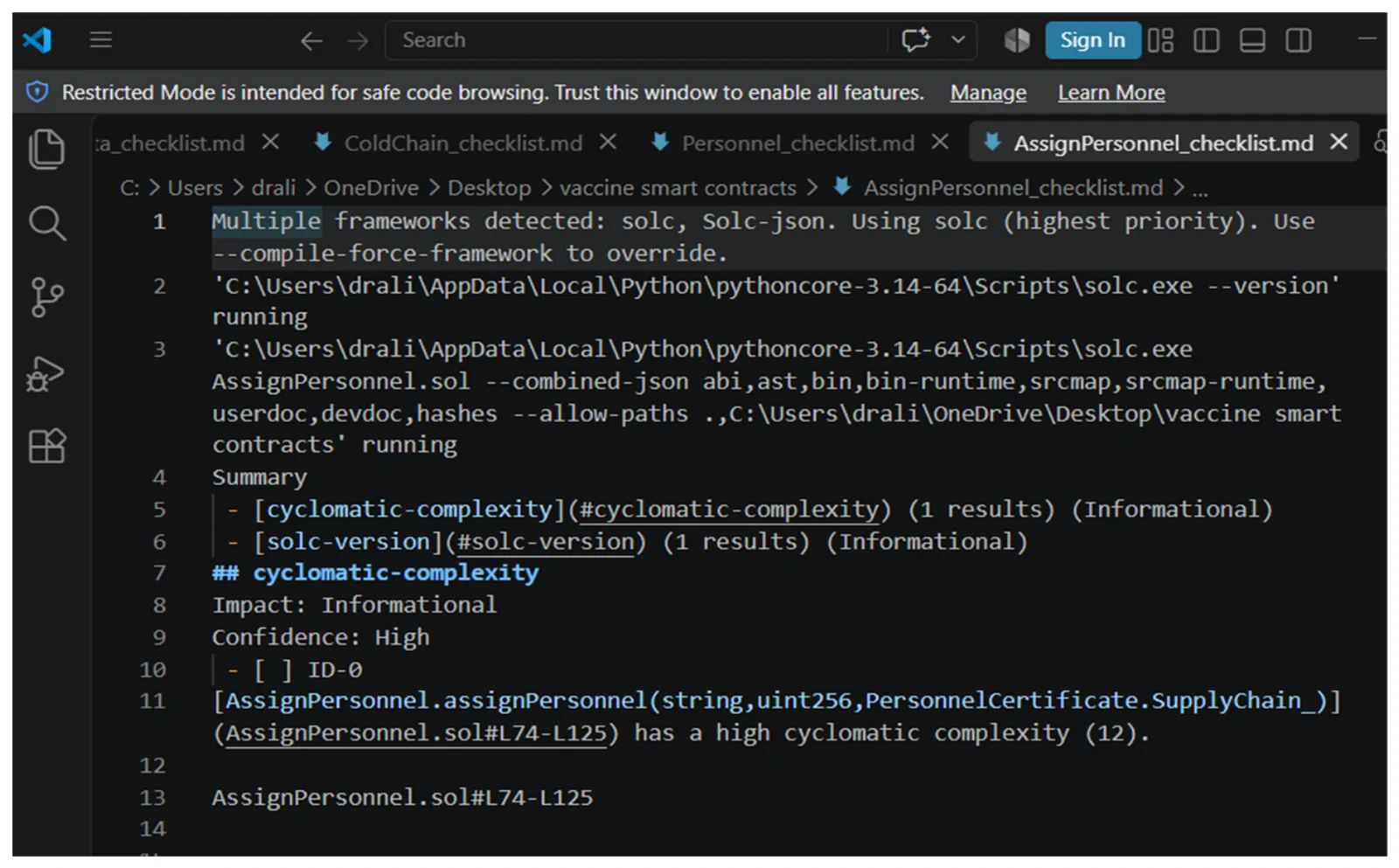
Slither report for the AssignPersonnel smart contract.

The system additionally addresses a comprehensive set of privacy considerations relevant to the storage and management of sensitive health and identity data:

- Data Storage Architecture: In the current prototype implementation on the public Ethereum blockchain, beneficiary personal profiles, vaccination event records, adverse event reports, and cold chain violation histories are stored directly on-chain. For production deployments, encryption of sensitive personal fields or migration to a permissioned blockchain architecture would be required to meet applicable data protection requirements.
- Beneficiary photographs are stored off-chain on IPFS, configured as a private node operated by the health authority and accessible only to authorized system participants. The content hash of each photograph is stored on-chain to ensure integrity verification while preserving confidentiality through the private node configuration, as the content hash alone does not grant access to the photograph without the private node.
- Encryption and Key Management: For production deployments, encryption of sensitive personal fields could be applied using established cryptographic and healthcare information security standards, with key management protocols designed in accordance with recognized healthcare information security frameworks and managed by the deploying health authority.
- Access Control and Revocation: The smart contract includes a revokeAdmin function and an *adminList* mapping that together control and restrict all write access to authorized administrators. Any administrator address can be immediately revoked by the government account, addressing the risk of malicious insiders and compromised endpoints. Transactions previously executed by a revoked administrator remain on-chain as an immutable and tamper-resistant accountability record, but no further write operations can be performed by that address following revocation.
- Consent Management: Consent for the collection and storage of personal and medical data is managed off-chain at the point of first dose administration, where the health administrator obtains the beneficiary’s informed written consent before any on-chain registration proceeds. The signed consent form is retained in the health facility’s records management system, consistent with established healthcare data governance practice.
- Data Minimization: All data fields stored by the system are clinically justified for the purposes of vaccine safety surveillance and adverse event analysis. Demographic information, including gender, marital status, date of birth, chronic disease history, and special needs, is essential for identifying demographic patterns in adverse event reporting, such as determining whether specific side effects are associated with particular age groups, genders, or individuals with pre-existing conditions. Such demographic-clinical linkage is a core requirement of post-marketing vaccine pharmacovigilance and is mandated by international vaccine safety monitoring frameworks.
- Data Retention: Vaccination records, adverse event reports, and cold chain violation histories are safety-critical public health data that national health governance frameworks and international vaccine safety reporting obligations require to be retained permanently. Unlike commercial personal data, medical and vaccination records are in many jurisdictions required to be retained for extended periods for epidemiological analysis and legal accountability, though specific retention obligations vary by applicable national and regional regulatory frameworks. The immutability of blockchain records therefore aligns with this legal requirement rather than conflicting with it, making blockchain a particularly suitable architecture for vaccination program data management.
- Right to Erasure: For jurisdictions where applicable data protection frameworks extend erasure rights to health records, an off-chain storage approach could be adopted at the deployment level, where sensitive personal identifiers are stored in a secure database managed by the health authority and only their cryptographic hashes are stored on-chain. Deletion of the off-chain personal data record renders the on-chain hash unresolvable, approximating erasure while preserving the integrity of the immutable audit trail of vaccination events. This approach requires no changes to the smart contract code.
- Linkability and Network Architecture: The proposed system is implemented and tested on the public Ethereum blockchain, which was selected for its transparency, decentralization, and trustless execution properties, ensuring that no single entity can alter or suppress records. RPC node security and DNS integrity are addressed through standard secure infrastructure practices at the deployment level. For implementations where data access restriction is prioritized over transparency, deployment on a permissioned blockchain architecture operated by authorized health authority stakeholders remains a viable option that preserves the system’s core smart contract logic without modification. The risk of linkability attacks is substantially mitigated in a permissioned deployment where network participation is restricted to authorized consortium members, eliminating external linkability attack vectors entirely.
- Record Amendment: In cases where a recorded side effect contains an error, the system supports the addition of amendment records rather than deletion or overwriting of the original. The *amendSideEffect* function, added in the revised smart contract, allows an authorized administrator to add a correction record referencing the original. The original record is preserved consistent with blockchain immutability and medical record-keeping best practice, while the amendment is permanently and transparently recorded alongside it. This approach is demonstrably stronger than centralized systems where records can be silently modified, as it provides a complete and auditable history of all data entries and corrections.
- Static Code Analysis and Vulnerability Assessment: The smart contracts were compiled using Remix IDE, which verifies compilation and run-time correctness. Compilation success confirms the absence of syntax and run-time errors. Static security analysis was conducted using Slither version 0.11.6. No high or medium severity vulnerabilities were identified across any of the four smart contracts. All findings were informational notices and optimization suggestions. Figure 24 shows the Slither report for the *VaccineColdSupplyChain* smart contract. Figure 25 shows the Slither report for the VaccineData smart contract. Figure 26 shows the Slither report for the *PersonnelCertificate* smart contract. Figure 27 shows the Slither report for the *AssignPersonnel* smart contract.

### 8.3. Performance and Scalability Analysis

Performance and scalability evaluation was conducted across two complementary environments. The four smart contracts were deployed and verified on the Ethereum Sepolia public testnet, confirming real-world deployment feasibility. Scale experiments were subsequently conducted on a controlled local Ethereum simulation environment using Hardhat Network v2.22.0 at transaction volumes of 100, 1000, 10,000, and 100,000, consistent with standard blockchain systems research methodology [120]. A fundamental property of Ethereum smart contracts governs the relationship between the tested scale and national deployment scale: each function executes independently and deterministically, with gas consumption fixed per transaction regardless of the total number of transactions previously processed by the contract. The gas costs reported in Table 9 and Table 10 therefore directly represent the per-event cost at the tested scales, and are expected to remain consistent at larger deployment scales given the deterministic nature of EVM execution.

**Figure 28:**
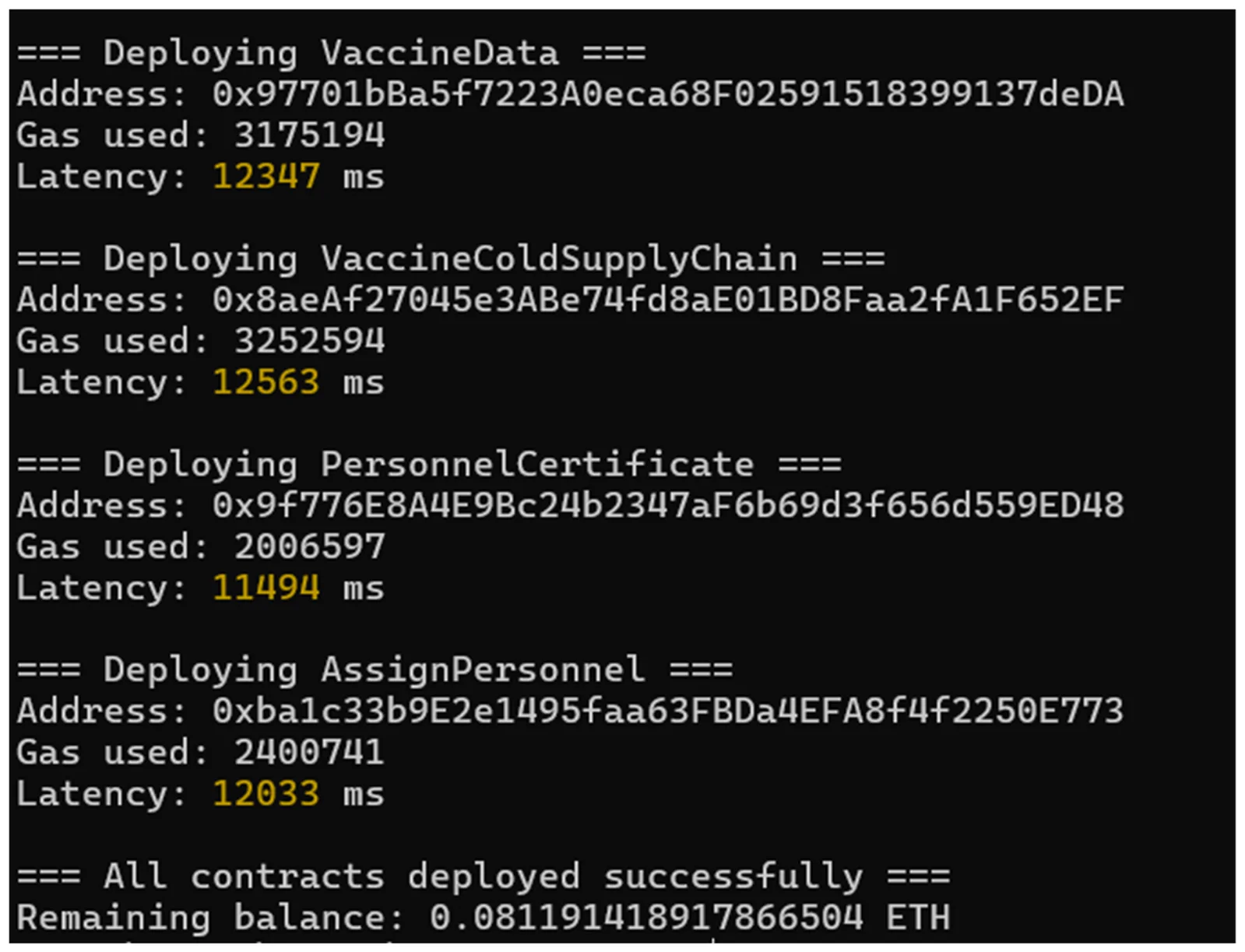
PowerShell output of the four smart contract deployments on Ethereum Sepolia testnet.

Table 11 presents the deployment results for all four smart contracts on the Ethereum Sepolia public testnet. The deployment latency of approximately 5 to 13 s per contract reflects Ethereum’s Proof-of-Stake consensus mechanism with an average block time of approximately 12 s. Figure 28 shows the PowerShell output of the four smart contract deployments on the Ethereum Sepolia public testnet, with contract addresses, gas consumption, bytecode size, and network confirmation latency.

Latency values represent the average of three independent deployment runs on the Ethereum Sepolia public testnet. Gas consumption and bytecode size are deterministic and identical across all runs.

**Figure 29:**
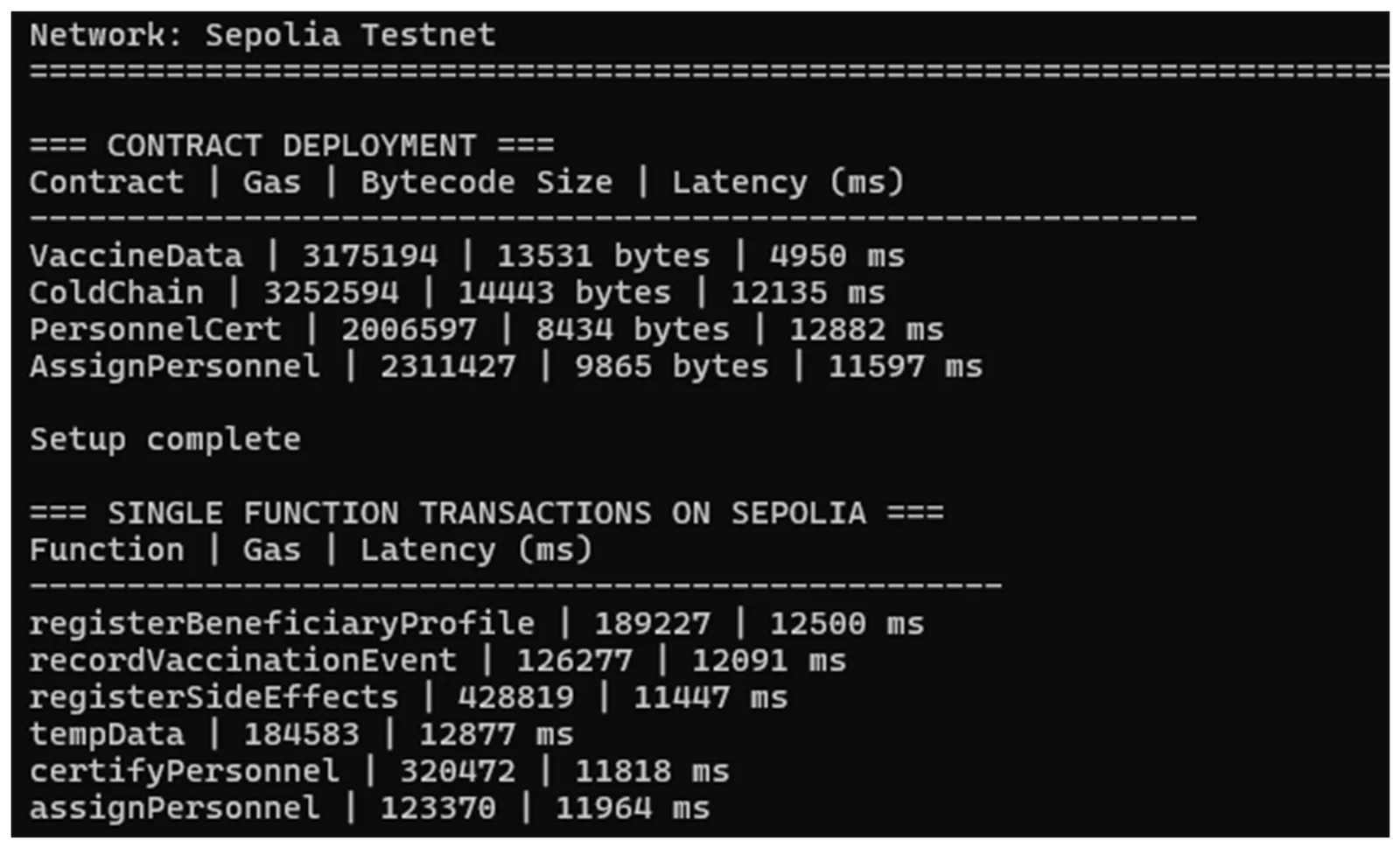
PowerShell output of single-function transactions on the Ethereum Sepolia testnet.

Table 12 presents the gas consumption and real network confirmation latency for a single execution of each key system function on Sepolia. The latency values average approximately 12 s per transaction, consistent with Ethereum’s Proof-of-Stake block time of approximately 12 s. Figure 29 presents the PowerShell output of single-function transactions on the Ethereum Sepolia public testnet, showing gas consumption and real network confirmation latency for all six key system functions.

Gas consumption values reported across Table 8, Table 9, Table 12 and Table 13 reflect measurements conducted in different environments for different purposes. Table 8 and Table 9 report gas values measured in Remix IDE for economic cost analysis at the ETH price recorded at the time of testing. Table 12 reports Sepolia testnet measurements confirming real network deployment feasibility and Table 13 reports Hardhat local simulation measurements for scale performance evaluation. Minor variations in gas values across environments are normal and expected, attributable to differences in EVM configuration, storage slot initialization state, and network-level access cost rules under EIP-2929, and do not affect the validity of the reported results for their respective purposes.

**Figure 30:**
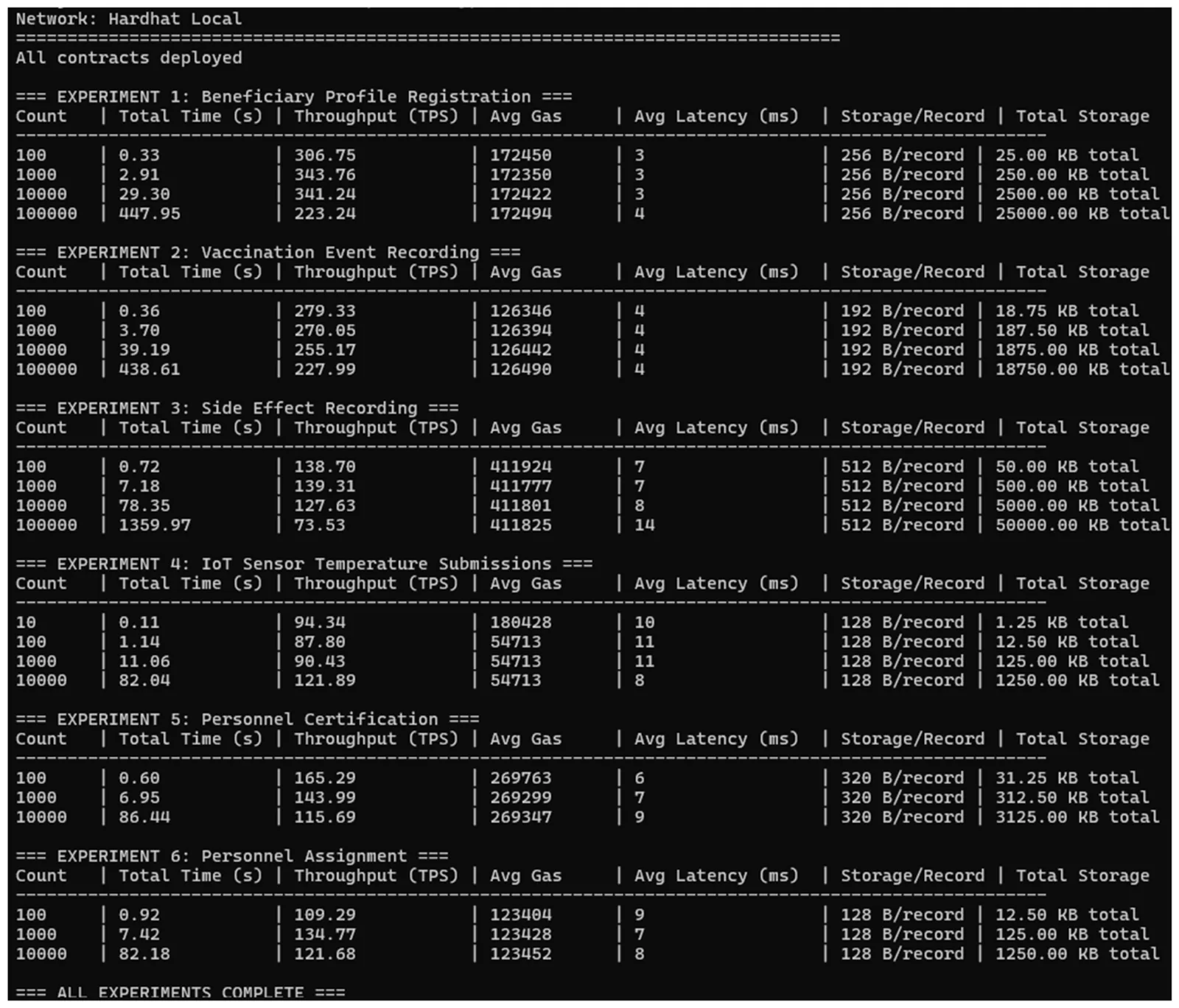
PowerShell of the scale performance experiments conducted on the Hardhat environment.

Table 13 presents the scale experiment results for all six system functions. Gas consumption remained stable and deterministic across all scales for all functions, confirming that per-transaction costs are independent of system load and accumulated data volume. Storage growth is linear and predictable, as each transaction writes a fixed number of storage slots determined by the smart contract data structures. At 100,000 beneficiaries receiving two doses each, total on-chain storage for profiles and vaccination events would be approximately 25 MB and 18.8 MB, respectively. The first IoT sensor submission in each experiment incurred higher gas (180,428) than subsequent submissions (54,713) due to the EIP-2929 cold storage access cost for initializing new storage slots; subsequent submissions benefit from the lower warm access cost, and in operational deployment this cold access cost is incurred only once per contract deployment. It is also noted that the sensor sampling frequency and the blockchain commit frequency are distinct operational parameters. The system is designed to commit only confirmed violation events to the blockchain rather than raw continuous sensor readings, as described in Section 4.2, which ensures that the transaction volume remains bounded and proportional to the violation rate rather than the sampling rate. Cold chain violation storage is therefore bounded by the actual violation rate rather than total sensor submissions. Figure 30 presents the PowerShell output of the scale performance experiments conducted on the Hardhat local Ethereum simulation environment, showing throughput, latency, gas consumption, and storage growth across all six system functions at transaction volumes of 100, 1000, 10,000, and 100,000.

The Ethereum base layer supports a throughput of approximately 13 to 25 transactions per second [120]. All transactions are atomic: if a transaction fails for any reason, the Ethereum Virtual Machine guarantees a complete revert with no partial writes, ensuring on-chain state consistency regardless of failure. The network operates across thousands of globally distributed nodes providing inherent fault tolerance without any application-level recovery mechanism. If the hardware oracle becomes temporarily unavailable, no on-chain data is corrupted and oracle operation resumes without any recovery procedure. With respect to concurrent user behavior, multiple participants submitting transactions simultaneously do not cause state inconsistencies, as Ethereum processes all transactions in a globally ordered sequence; transactions are queued in the mempool and processed sequentially within each block, with access control modifiers in the smart contract functions preventing state conflicts from unauthorized concurrent access. For deployments requiring higher throughput or lower latency, Layer-2 rollup solutions and permissioned blockchain architectures are discussed in Section 8.1.

## 9. Conclusions, Limitations and Future Work

Throughout history, diseases and pandemics have plagued the world; in many cases they are lethal, with detrimental social and economic impacts. The majority of the time, medical researchers fight disease by creating vaccinations. This occurred when COVID-19 expanded globally, killing people and impairing the healthcare, financial, and social systems. Worldwide vaccination campaigns have been launched. However, as vaccinations took an unprecedentedly short amount of time to produce, concerns have been raised regarding any potential side effects that may or may not exist and how severe they might be if they exist. Additionally, the cold supply chain needs to be regularly controlled and monitored because vaccines are delicate medicinal products. Therefore, it is essential to investigate the source of any observed side effect and whether it may be linked to a manufacturing issue or supply chain mishandling. The proposed system enables healthcare authorities and clinical investigators to conduct such investigations through a complete, tamper- resistant, and traceable data record. The establishment of this link also requires a successful beneficiary registration procedure. Moreover, vaccines’ sensitivity to light and temperature increases the requirement for expert management of their intricate supply chain. Well-trained staff members are a critical component of good cold supply chain management, as the cold supply chain of vaccines has a significant impact on their efficacy. Thus, an effective system is required to certify and validate the credentials of individuals assuming positions involving the cold supply chain and handling temperature-sensitive vaccinations. In order to address this, our study created a comprehensive system that combines cold supply chain management for vaccines with beneficiaries’ registration records, vaccine adverse-effects follow-up, and supply chain personnel certification and employment. Blockchain was chosen to develop this system as it has proven to be exceptionally effective and efficient in diversified applications. This research developed a comprehensive vaccine management and safety platform consisting of four interlinked smart contracts. The vaccine cold supply chain management and the beneficiaries’ side effects smart contracts are launched by the government as the major stakeholder in the vaccine campaign, which is responsible for its success and safety. The supply chain management smart contract provides transparent and traceable functionality for vaccine shipment. It also captures any violations and associates them with the lot number, time and location of occurrence. The vaccine side effects smart contract provides a complete database for administered beneficiaries, their metadata and any witnessed side effects. This smart contract provides the link to the supply chain management smart contract as it registers the vaccine lot number of the inoculation administered for each beneficiary. The cold supply chain personnel certification issuance smart contract is the third smart contract that gets deployed by a training entity to generate and authenticate personnel certification. The personnel employment smart contract is launched by the hiring entity, which is mostly the government importing the vaccine, while adding other stakeholders to the system who are interested in employing experts in different chains within the vaccine cold supply chain. This fourth smart contract verifies whether a person is qualified to be assigned any job in the cold supply chain. If an attempt is made to employ an uncertified person at any chain or even employ a certified person at an inappropriate chain, the system will throw an error. The platform introduced five architectural contributions that, to our knowledge and based on the comparative analysis of the surveyed literature, have not been simultaneously addressed in any existing system: forensic investigation support through bidirectional traceability that provides the interconnected data record needed to investigate whether an observed adverse effect may be linked to cold chain handling conditions or production factors; a class of demographic–clinical–supply chain analysis that structurally cannot exist without data integration; an automated smart contract compliance gate for personnel assignment; a tamper-resistant accountability record that provides the verifiable data trail required for accountability investigation; and a side effect amendment model that reconciles blockchain immutability with medical record governance requirements. The created smart contracts were successfully tested and simulated, producing the intended outcomes across all defined functional scenarios. The results demonstrated a functionally consistent and effective system design within the tested scenarios, supported by functional testing across all defined scenarios and performance and scalability evaluation conducted on both the Ethereum Sepolia public testnet and a controlled local simulation environment. Large-scale empirical validation under live operational conditions is identified as a future direction. This study has significant importance in multiple dimensions. It provides a structured and tamper- resistant data platform for vaccine aftermath information, representing a valuable resource for health authorities, governments, vaccine manufacturers, and international organizations, subject to real-world validation and regulatory compliance in the deployment context. The collected data could be further used in big data analysis studies to extract valuable information about the vaccine that shall be used to improve its production process. Also, in the absence of observed side effects, it may contribute to reassuring the public and the health authorities regarding the safety of the vaccines and may assist in informing the development and approval procedures, subject to real-world validation. Moreover, our study provides a supply chain tracking system that could potentially be adapted for other types of vaccines or medical or pharmaceutical products that require continuous monitoring and a secure source of information, subject to appropriate validation in those contexts. It could also be applied in the food industry. Additionally, the platform provides a much-needed solution in the field of supply chain management practices that reduces the risk of human error when transferring vaccine medicines.

Several limitations are acknowledged in the context of this work. The system was evaluated using representative simulated scenarios, consistent with the standard methodology for prototype research, with validation using real vaccine and healthcare data and evaluation with healthcare stakeholders identified as essential future directions. Sensor calibration and oracle authentication are hardware operational considerations addressed at the deployment level, with device-level authentication protocols identified as a future implementation direction. Privacy governance is addressed through stakeholder-only access controls, with a permissioned blockchain architecture recommended for production deployment to strengthen regulatory compliance. The system provides the data infrastructure for causal investigation. The determination of whether a specific adverse event is linked to a cold chain or production condition remains the domain of clinical investigators and epidemiologists.

Future work will advance this research through three principal directions: participatory validation with healthcare administrators, supply chain operators, and government health authorities to assess operational feasibility and refine the system to real-world requirements; investigation of permissioned blockchain and Layer-2 deployment architectures to optimize privacy governance, regulatory compliance, and transaction cost management at national vaccination campaign scale; and implementation of secure communication channel protocols between physical sensors and the oracle, including hardware-level oracle authentication and tamper-evident sensor communication, to strengthen the IoT data integrity layer for production deployments.

## Figures and Tables

**Table 1:** COVID-19 vaccine cold storage requirements [6,7].

Vaccine	Type	Recommended Freezer Storage Temperature
Pfizer-BioNTech	mRNA	−60 °C to −80 °C (−76 to −112 °F)
Moderna	mRNA	−15 °C to −25 °C (5 °F to −13 °F)
Johnson & Johnson	Viral vector	2 °C to 8 °C (36 °F to 46 °F)
Astrazeneca	Viral vector	2 °C to 8 °C (36 °F to 46 °F)
Sinopharm	Inactivated virus	2 °C to 8 °C (36 °F to 46 °F)

**Table 2:** Literature review comparison.

Related Work	Blockchain Platform	IoT Monitoring	Beneficiary Registration	Side Effects Recording	Cold-Chain Traceability	Personnel Certification	Employment Verification	Implementation	Security Analysis	Performance/Scalability
[22]	Hybrid Private and Public Distributed Ledger	◑ Commuter-sourced geolocation monitoring only, no temperature sensors	✗	✗	◑ Physical distribution shipment tracking only, no cold chain temperature management	✗	✗	✓	✗	✗
[25]	Unspecified	✓	✗	✗	✓	✗	✗	◑ Platform description only, no technical implementation details	✗	✗
[27]	Cross-chain PoA Blockchain	✓	✗	✗	✓	✗	✗	✓	✗	✓
[28]	Consortium Blockchain	✓	✗	✗	✓	✗	✗	✓	✗	◑ Gas consumption analysis only
[29]	Ethereum	✓	✗	✗	✓	✗	✗	✓	✗	✓
[30]	unspecified (conceptual framework)	✓	✗	✗	✓	✗	✗	✓	✗	◑ ML demand forecasting evaluated, blockchain performance not evaluated
[31]	Hyperledger Fabric 2.4	✓	✗	✗	✓	✗	✗	◑ BIM and MCDA hybrid evaluation	✓	✗
[32]	Hyperledger Fabric	✓	✗	✗	✓	✗	✗	✓	✗	✓
[59]	Ethereum	✗	✓	✗	✗	✗	✗	✓	◑ Basic security discussion	✗
[60]	Ethereum	✗	◑ COVID test/vaccine certificate issuance, not full beneficiary profile	✗	✗	✗	✗	✓	◑ GDPR/KYC compliance discussion	✗
[61]	Ethereum	✓	◑ Self-registration by beneficiary using Merkle proof. No admin-verified profile registration	◑ Self-reported, not clinically verified by administrator	✓	✗	✗	✓	✗	✓
[62]	Ethereum	◑ Sensors monitor state of waste bags during shipment only.	✗	✗	✓	✗	✗	✓	✓	✗
[63]	Hyperledger Besu	✗	✗	✗	◑ Vaccine distribution tracking only	✗	✗	✓	✗	◑ LSTM demand forecasting evaluated, blockchain performance not evaluated
[64]	Ethereum	◑ IoT monitoring mentioned but not central contribution	◑ Privacy-preserving identity management only	✗	✓	✗	✗	✓	✗	◑ Scalability challenges identified
[65]	Conceptual	◑ data collection tool within model	✗	✗	◑ Transparency maximization objective, no smart contract	✗	✗	◑ Mathematical model validated	✗	◑ Optimization model sensitivity analysis
[66]	Hyperledger Fabric	✓	✗	✗	✓	✗	✗	✓	✗	✓
[82]	Hyperledger Fabric	✗	✗	✗	✗	◑ Credential verification discussed as one of five HR areas	◑ Employment onboarding modelled theoretically	✗	✗	✗
[84]	Ethereum	◑ IoT healthcare context only	✗	✗	✗	✓	✗	✓	◑ Privacy-focused discussion	✗
[85]	Hyperledger Fabric	✓	✗	✗	✗	◑ Training data sharing only, no certificate generation	✗	✓	✗	✗
[86]	Ethereum	✗	✗	✗	✗	✓ Healthcare training certificate management	✗	✓	✗	✗
[87]	✗	✗	✗	✗	✗	◑ Physician credentialing proposed conceptually, no smart contract	◑ Work history verification across employers discussed, no implementation	✗	✗	✗
Proposed System	Ethereum	✓	✓	✓	✓	✓	✓	✓	✓	✓

✓ = Fully addressed, ◑ = Partially addressed, ✗ = Not addressed.

**Table 3:** Comparison of deployment platforms.

Criterion	Conventional/Authenticated Database	Permissioned Blockchain (e.g., Hyperledger Fabric)	Proposed Architecture (Blockchain-Based, Evaluated on Ethereum)
Write/record authority	Single administrator (e.g., government IT) holds unilateral write access	Distributed across endorsing peers from multiple organizations before commitment	Distributed across network validators; no single participant can unilaterally commit a record
Tamper-evidence after commit	Not inherent; administrator can alter records without independent detection	Inherent; records are cryptographically hash-chained across blocks	Inherent; records are cryptographically hash-chained across blocks
Cross-organizational auditability	Requires trust in the database owner’s audit logs, which are themselves editable	Each organization validates from its own peer node, independent of a shared administrator	Each participant can independently verify the ledger without relying on a shared administrator
Confidentiality between stakeholders	Managed via access control lists set by the administrator	Managed via channels and private data collections, restricting visibility to authorized organizations	Managed via permissioning at the smart contract/access-control layer
Automated, consistent rule enforcement	Business logic enforced by application code controlled by the database owner	Enforced via chaincode (smart contracts), executed identically across endorsing peers	Enforced via smart contracts, executed identically across network participants
Underlying trust assumption	Full trust required in the single administering entity	Trust distributed across participating organizations’ endorsement policies	Trust distributed across network validators

**Table 4:** The inserted vaccine supply chains.

Chain Name	Chain EA	Chain No.
Manufacturer	0xAb8483F64d9C6d1EcF9b849Ae677dD3315835cb2	1
Transit_Mfr_Apt	0x4B20993Bc481177ec7E8f571ceCaE8A9e22C02db	2
Aircraft_Airborne	0x78731D3Ca6b7E34aC0F824c42a7cC18A495cabaB	3
vTransit_Apt_National	0x617F2E2fD72FD9D5503197092aC168c91465E7f2	4
National_Stg	0x17F6AD8Ef982297579C203069C1DbfFE4348c372	5
Transit_National_Regional	0x5c6B0f7Bf3E7ce046039Bd8FABdfD3f9F5021678	6
Regional_Stg	0x03C6FcED478cBbC9a4FAB34eF9f40767739D1Ff7	7
Transit_Regional_Med	0x1aE0EA34a72D944a8C7603FfB3eC30a6669E454C	8
Medical_Center	0x0A098Eda01Ce92ff4A4CCb7A4fFFb5A43EBC70DC	9

**Table 5:** Training modules description.

Module	Description
Module 1	Vaccine presentation, storage requirement and shelf life
Module 2	Vaccine management and administration considerations.
Module 3	Procedures for shipping and handling thermal shipping containers.
Module 4	Cold chain equipment requirements and vaccine deployment policies.
Module 5	UCC equipment and temperature monitoring device (TMD) selection for UCC hub.
Module 6	UCC system readiness
Module 7	Transport and storage of vaccines at lower service points
Module 7.1	Transportation of ULT frozen vaccines to subnational stores and service points
Module 7.2	Equipment options for transporting and storing vaccines at lower distribution points
Module 8	Management of vaccine storage and transport at service point
Module 9	Variant containing vaccine formulations exclusively for booster dose administration.

**Table 6:** Targeted audience and permitted training modules.

Audience	Permitted Modules (Eligible for Certification)
Decision Makers	4-5-6
Supervisors	1-2-8-9
Immunization Workers	1-2-8-9
Health Care Workers	1-2-8-9
Cold Chain Managers	All Modules
Supply Chain Officers	All Modules

**Table 7:** Personnel and designated chains.

Personnel	Chain Designation
Decision Makers	All Chains
Supervisors	National Storage, Regional Storage & Medical Center
Immunization Managers	National Storage, Regional Storage & Medical Center
Health Care Workers	National Storage, Regional Storage & Medical Center
Supply Chain Officers	Transit from manufacturer to airport, Aircraft Airborne, Transit from airport to national storage, transit from national to regional storage, transit from regional storage to medical center.
Cold Chain Managers	Transit from manufacturer to airport, Aircraft Airborne, Transit from airport to national storage, transit from national to regional storage, transit from regional storage to medical center.

**Table 8:** Cost of executing functions by the cold supply chain smart contract.

Function	Caller	Tx Cost “Gas”	Transaction Fee
			USD
Supply chain smart contract constructor	System Creator “Government”	1,859,158	9.56
Registration and side effects smart contract constructor	System Creator “Government”	2,134,011	10.98
addChain	System Creator “Government”	113,588	0.584
startProduction	Manufacturer	47,722	0.246
updateVaccineLocation	Supply Chains	30,831	0.159
tempData	Temp. Sensors	275,547	1.42
lightExposure	Light Sensor	272,802	1.40
addAdmin	Governement and Admin	127,161	0.657
registerBeneficiaryProfile	Admin	187,179	0.967
recordVaccinationEvent	Admin	143,925	0.741
registerSideEffects	Admin	425,642	2.19

**Table 9:** Cost of executing functions by the certification smart contract.

Function	Caller	Tx Cost “Gas”	Transaction Fee USD
Certification Smart contract deployment	Trainer (Pfizer)	1,077,618	5.52
Employment Smart contract deployment	Employer	1,566,085	8.05
Add admin	Trainer, employer & admin	97,389	0.5
Certify personnel	Admin	317,695	1.63
Set address	Employer	46,196	0.238
Assign personnel	Admin	121,949	0.631

**Table 10:** Synthetic workload-based cost scenario.

Operation	Cost per Transaction (USD)	Estimated Volume	Estimated Total Cost (USD)
System deployment (both contracts)	$20.54	Once per campaign	$20.54
Beneficiary profile registration	$0.967	1,000,000 beneficiaries	$967,000
Vaccination event recording (2 doses)	$0.741	2,000,000 dose events	$1,482,000
Side effect recording	$2.19	~2602 records (130.1 per 100,000 doses [111] × 2M doses)	$5,698.38
Cold chain deployment (100 batches)	$9.56 per batch	100 batches	$956.00
Supply chain setup (addChain ×9 per batch)	$5.256 per batch	100 batches	$525.60
Location updates (×8 per batch)	$1.272 per batch	100 batches	$127.20
Temperature violation recording	$1.42 per violation	~117 violations (13% worst-case alert rate across 900 batch stages) [110]	$166.14
Personnel certification (500 personnel)	$1.63	500 personnel	$815
Personnel assignment (500)	$0.631	500 assignments	$315.50
Total Campaign Estimate		1,000,000 beneficiaries, 100 batches, 500 personnel	~$2,457,624 (Incl. 13% worst-case sensor alert overhead)

**Table 11:** Smart contract deployment on Ethereum Sepolia public testnet.

Smart Contract	Gas Used	Bytecode Size (Bytes)	Latency (ms)
VaccineData	3,175,194	13,531	11,044
VaccineColdSupplyChain	3,252,594	14,443	11,894
PersonnelCertificate	2,006,597	8434	12,475
AssignPersonnel	2,311,427	9865	11,308

**Table 12:** Single function transaction results on Ethereum Sepolia public testnet.

Function	Contract	Gas Used	Latency (ms)
registerBeneficiaryProfile	VaccineData	189,227	12,500
recordVaccinationEvent	VaccineData	126,277	12,091
registerSideEffects	VaccineData	428,819	11,447
tempData	VaccineColdSupplyChain	184,583	12,877
certifyPersonnel	PersonnelCertificate	320,472	11,818
assignPersonnel	AssignPersonnel	123,370	11,964

**Table 13:** Scale performance experiments on Hardhat local simulation environment.

registerBeneficiaryProfile	100	0.32	309.60	172,450	3	256	25.00 KB
	1000	2.71	369.69	172,350	3	256	250.00 KB
	10,000	29.31	341.19	172,422	3	256	2500.00 KB
	100,000	406.51	246.00	172,494	4	256	25,000.00 KB
recordVaccinationEvent	100	0.92	109.17	126,346	9	192	18.75 KB
	1000	10.75	92.99	126,394	11	192	187.50 KB
	10,000	48.06	208.09	126,442	5	192	1875.00 KB
	100,000	596.49	167.65	126,490	6	192	18,750.00 KB
registerSideEffects	100	0.98	102.15	411,924	10	512	50.00 KB
	1000	10.49	95.30	411,777	10	512	500.00 KB
	10,000	106.06	94.29	411,801	11	512	5000.00 KB
	100,000	1496.50	66.82	411,825	15	512	50,000.00 KB
tempData (IoT)	10	0.34	28.99	180,428	34	128	1.25 KB
	100	2.48	40.34	54,713	25	128	12.50 KB
	1000	18.65	53.63	54,713	18	128	125.00 KB
	10,000	281.48	35.53	54,713	28	128	1250.00 KB
certifyPersonnel	100	1.47	67.89	269,763	15	320	31.25 KB
	1000	14.35	69.68	269,299	14	320	312.50 KB
	10,000	140.06	71.40	269,347	14	320	3125.00 KB
assignPersonnel	100	1.35	73.80	123,404	13	128	12.50 KB
	1000	12.88	77.63	123,428	13	128	125.00 KB
	10,000	106.83	93.61	123,452	11	128	1250.00 KB

## Data Availability

No new data were created or analyzed in this study. Data sharing is not applicable to this article.
